# *Bombyx Mori* Silk Fibroin as a Sustainable Organocatalyst for Diastereoselective Michael Additions

**DOI:** 10.1002/cssc.202500584

**Published:** 2025-07-14

**Authors:** Carola Ricciardelli, Giorgio Rizzo, Pietro Cotugno, Antonio Salomone, Daniela Trisciuzzi, Orazio Nicolotti, Erica Colaprico, Cosimo D. Altomare, Gianluca M. Farinola

**Affiliations:** ^1^ Department of Chemistry University of Bari Aldo Moro Via E. Orabona 4 Bari 70125 Italy; ^2^ Department of Pharmacy‐Pharmaceutical Sciences University of Bari Aldo Moro Via E. Orabona 4 Bari 70125 Italy

**Keywords:** bio‐polymeric organocatalyst, biopolymer, heterogenous catalysis, Michael additions, silk fibroin

## Abstract

Powdered silk fibroin (PSF) extracted from *Bombyx mori* cocoons is reported as a heterogeneous organocatalyst in the selective Michael 1,4 addition of nitromethane to *α,β*‐unsaturated carbonyl compounds, affording Michael adducts in almost quantitative yields, with complete antidiastereoselectivity and in mild conditions. PSF proved to be reusable for more than 50 recycles without any loss of catalytic activity. In silico studies suggest the presence of an enzyme‐like pocket as the active catalytic site, pointing out fibroin fibers as a heterogeneous biological organocatalyst.

## Graphical abstract

Silk fibroin, natural biopolymer, has been successfully used as green catalyst for Michael addition reactions under mild conditions. Its pseudoenzymatic pocket enables enzyme‐like catalytic behavior, driving stereoselectivity, and highlighting a sustainable, nature‐inspired catalytic approach.

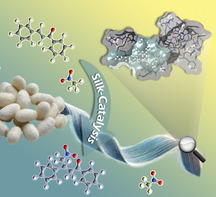

## 1 Introduction

Michael‐type Csp^3^—Csp^3^ bond forming reactions are well‐known base‐catalyzed processes that occur via conjugate addition of a variety of nucleophiles to activated *α,β*‐unsaturated carbonyl compounds.^[^ ^1^ ^]^ These reactions are profitable routes to plenty of products with applications ranging from biomedicine and pharmaceutics to optoelectronics and, more in general, materials science.

Michael‐type adducts result from the nucleophilic attack to the *β* position of the substrate conjugated system, according to a thermodynamic controlled 1,4‐addition mechanism. However, the nucleophilic 1,2‐addition to the carbonyl can also occur under kinetic control, in the presence of highly polarized carbonyl groups.

Common carbon nucleophiles used in Michael reactions result from the deprotonation in the *α* position (pK_a_ ≈ 19) of *β*‐ketoesters by strong bases, activated by the presence of the keto group.^[^ ^2^ ^]^ Deprotonation of nitroalkanes in the *α* position (pK_a_ ≈ 10) is even easier and requires weak bases, since the —NO_2_ group features a stronger electron withdrawing effect compared to the carbonyl group. For this reason, *α*‐nitro carbanions are very convenient nucleophiles for enone substrates, leading to *β*‐nitroalkyl Michael adducts, which are useful intermediates for many applications, including the synthesis of pharmaceutical products.^[^ ^3^ ^]^

Protocols that do not require the use of strong bases are desired to perform Michael reactions under mild experimental conditions and to minimize waste by‐products. Heterogeneous catalysts with basic reactive sites that promote in situ generation and activation of the nucleophile attract great interest. Among them, mineral clays,^[^ ^4^ ^]^ heavy metal nanoparticles,^[^ ^5^ ^]^ basic metal oxides^[^ ^6^ ^]^ have been reported in the literature to catalyze Michael‐type addition of nitroalkanes to enones, although these protocols often suffer from long reaction time, moderate or even low yields, high loading and low recyclability of the catalysts.

Heterogeneous catalysts supported on biopolymers such as DNA‐^[^ ^7^ ^]^ or lignin‐based^[^ ^8^ ^]^ metal coordinated complexes, and amine grafted polysaccharides,^[^ ^9^ ^]^ are promising for low costs, abundance of raw sources, and possibility to perform enantioselective Michael addition in water. However, poor recyclability was found for the most bioderived catalysts. For example, alginate gel catalytic supports suffer from loss of activity due to pore occlusion preventing their reuse,^[^ ^10^ ^]^ chitosan‐based catalysts’ activity is strictly dependent on the deacetylation degree of chitin precursors,^[^ ^11^ ^]^ and their recyclability is limited by high chitosan hygroscopicity,^[^ ^12^ ^]^ which makes necessary to stabilize chitosan by covalent grafting to synthetic polymeric supports.

A variety of amino acid‐ or peptide‐based catalysts have been investigated. For example, potassium salts of L‐leucine and L‐threonine were proven to catalyze the Michael addition of enolate anions from aldehydes to maleimide substrates, but the reaction requires the presence of strong base (KOH).^[^ ^13^ ^]^ Similarly, a strong base is required when using *α,β*‐dipeptides as chiral organocatalysts in the asymmetric addition of aldehydes to *N*‐aryl maleimides or nitro olefins.^[^ ^14^ ^]^

L‐phenylalanine adsorbed in situ on alumina or clay minerals was also investigated in enantioselective Michael additions, but its reuse was limited to three recycles.^[^ ^15^ ^]^

The use of tripeptide‐based catalysts in Michael additions is hindered by the difficult preparation and purification procedures.^[^ ^16^ ^]^

On this ground, our attention was attracted by silk fibroin (SF) as a potential biopolymeric organocatalyst. SF is a protein‐based biopolymer extracted from the silkworm *Bombyx mori* cocoons, the most abundant and easy to handle source of silk in terms of easy purification, availability, recyclability, and catalytic stereoselectivity.^[^ ^17^ ^]^

SF fibroin fibers are composed mainly of L‐glycine (Gly, 45%), L‐alanine (Ala, 30%), L‐serine (Ser, 12%), L‐tyrosine (Tyr, 5%), and L‐valine (Val, 1.8%) as the main amino acids, which are arranged in repetitive Ala‐Gly‐Ala‐Gly‐X‐Ala sequences, where X stands for Tyr or Ser.^[^ ^18^ ^]^ They constitute hydrophobic crystalline *β*‐sheet regions held together by intramolecular and intermolecular hydrogen bonds, Van der Waals forces and hydrophobic interactions.

Toughness and elasticity of SF result from the alternation of crystalline and amorphous regions due to the presence of crystalline domains embedded into hydrophilic disordered regions made of polar amino acids, such as glutamate, aspartate, threonine, tyrosine, and serine.^[^ ^19^ ^]^ The high content of antiparallel *β*‐sheet domains confers excellent mechanical properties superior to those of many other synthetic and natural polymers. Moreover, SF can be easily processed under mild aqueous conditions in different scaffolds, such as fibers, powders, sponges, films, tubes, and hydrogels.^[^ ^20^ ^]^

Due to its chemical structural and morphological features, SF has been recently investigated as a versatile and eco‐friendly support for covalent or noncovalent immobilization of either transition metal‐based catalysts or enzyme biocatalysts with retention of their biological activity.^[^ ^21^ ^]^ For example, Cu nanoparticles immobilized onto SF were recently reported to catalyze azide–alkyne cycloaddition reactions with good yield, regioselectivity, and catalyst reusability.^[^ ^22^ ^]^ SF supported Pd(0) catalyst was also proven to chemoselectively catalyze hydrogenation reactions of alkynyl, olefin, and azide functionalities in the presence of other unsaturated functional groups.^[^ ^23^ ^]^

In a recent work, our group demonstrated the outstanding efficiency and recyclability of a SF supported Pd(0) catalyst in Suzuki–Miyaura crosscoupling,^[^ ^24^ ^]^ Ullmann homocoupling,^[^ ^25^ ^]^ and Sonogashira coupling^[^ ^26^ ^]^ reactions of aryl halides, working without the use of phosphine ligands, in aqueous media, and at low temperatures yielding the products in short reaction times with high purity without the need of complex purification procedures.

To the best of our knowledge, literature reports only one example of use of *B. mori* SF as the organocatalyst in the Henry nitroaldol reaction of nitromethane with aromatic aldehydes activated by electron withdrawing substituents.^[^ ^27^ ^]^ The process is constrained not only by the requirement for electron‐withdrawing substituents on the aldehyde but also by limited SF recyclability and a lack of diastereoselectivity. The SF physical state was also reported to influence the reaction kinetics, with the best results observed for freeze‐dried SF processed from aqueous solution, whereas SF hydrogels were not successful.

We set up to demonstrate a general protocol where SF behaves as an eco‐friendly and efficient organocatalyst for diastereoselective Michael reactions of nitroalkane‐derived nucleophiles with enones or dienones, affording *β*‐nitroalkyl adducts under mild conditions with satisfactory reaction yields, full antidiastereoselectivity, and exceptionally high SF recyclability.

More interestingly, based on in silico investigations, we hypothesized that SF behaves as an enzyme‐like catalyst, due to the presence of a pseudoenzymatic pocket capable of promoting stereoselective reactions on substrates with suitable size, suggesting a direct cooperation of the polar amino acids of the amorphous regions of the protein.

## 2 Results and Discussion

### 2.1 Preparation and Characterization of the Catalyst

The catalyst was prepared shredding raw *B. mori* cocoons in ≈1 cm pieces and boiling them in Na_2_CO_3_ (0.02 M) aqueous solution to remove the external sericin coating and isolate the SF pure protein as shiny white fibers, that were air‐dried for 24 h. Powdered silk fibroin (PSF) was also obtained by a ball milling process reported in the experimental section.

**Figure 1:**
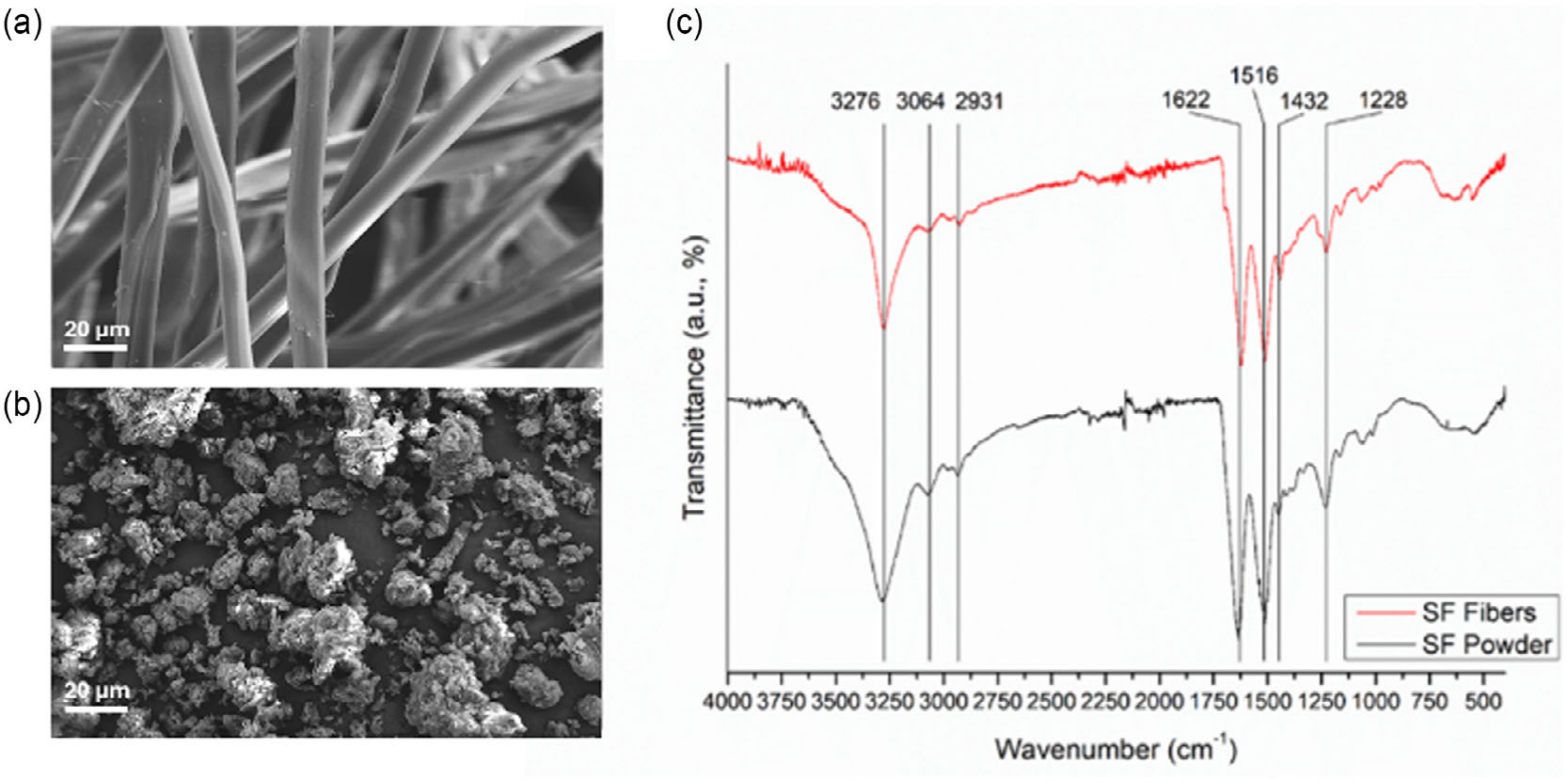
Characterization of the SF organocatalyst. a,b) SEM images of degummed and PSF, respectively. c) Comparison of ATR‐FTIR spectra of both samples, highlighting the typical protein bands that are not altered by the milling process.

**Figure** **1** shows scanning electron microscopy (SEM) images at two different magnifications and attenuated total reflectance‐Fourier transform infrared (ATR‐FTIR) spectra for both degummed SF and PSF samples. In our previous work, the smooth morphology of SF fibers was determined, with an average 9.9 ± 0.5 μm diameter. Fibers are arranged in single brins with typical hemicylindrical cross sections.^[^ ^28^ ^]^

Ball milling pulverization led to the complete destruction of the SF fibrous structure and to the formation of protein clusters with almost spherical morphology and heterogeneous distribution of average diameter ranging from 150 μm for big clusters to 10–20 μm for small clusters (SEM in Figure S2, Supporting Information).

FT‐IR spectra are relevant for elucidating SF structural features, since the amide bands are diagnostic of the polypeptide secondary structure. Both degummed SF and PSF show identical spectral features, suggesting that the milling procedure does not affect the polymeric hierarchical arrangement.^[^ ^28^, ^29^ ^]^ In particular, the amide A and amide B bands at 3276 cm^−1^ and 3064 cm^−1^, respectively, are the result of a Fermi resonance between the first overtone of the amide II band and the N—H stretching vibrations. The band corresponding to the stretching frequencies of C—H groups at 2931 cm^−1^ is also evident. The typical amide bands related to the presence of specific secondary structures such as *β*‐sheets or *α*‐helices fall at 1700–1500 cm^−1^. The amide I band is observed at 1622 cm^−1^, and it is mainly related to the C=O stretching vibrations and, to a less extent, to the N—H in‐plane bending, out‐of‐phase C—N stretching, and vibration of the amide system CNN. The amide II peak at 1516 cm^−1^ is due to the C—N stretching and N—H in‐plane bending modes. The amide III band at 1228 cm^−1^ arises from N—H in‐plane bending vibrations.

The FT‐IR spectra profiles of both SF and PSF are equivalent, indicating that the pulverization procedure has not affected the protein structure.^[^ ^30^ ^]^ To exclude the presence of possible catalytic contaminants or metallic impurities within the silk fibroin, energy dispersive X‐ray spectroscopy (EDS) analysis was performed. The results, reported in the Supporting Information, confirmed the absence of detectable heavy metals or other plausible catalytic species.

### 2.2 Silk Fibroin Catalyzed Michael Addition of Nitromethane to Chalcones

**Scheme 1:**
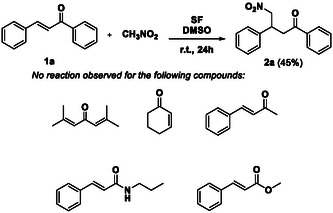
First investigation of the Michael‐type addition of nitromethane to a series of *α,β*‐unsaturated carbonyl compounds in the presence of the SF catalyst. Reaction conditions: Substrate (2.5 mmol), CH_3_NO_2_ (25 mmol), DMSO (2 mL), SF (50 mg). Yields were evaluated after column chromatography of the reaction mixture.

At the outset of our investigation, we focused on the suitability of SF as a catalyst in the Michael addition of nitromethane to a series of *α,β*‐unsaturated ketones reported in **Scheme** **1**. The reactions were performed according to the experimental conditions described by Kuhbeck et al. for nitroaldol Henry reactions, that is, room temperature for 24 h, in the presence of dimethyl sulfoxide (DMSO) as the solvent and degummed SF as the catalyst.^[^ ^27^ ^]^

In these conditions, the nucleophilic conjugated addition occurred only in the case of the (*E*)‐chalcone (**1a**), whereas no reaction was observed for the other substrates in Scheme 1.

Therefore, we selected (*E*)‐chalcone **1a** as the reference substrate to carry out a systematic study of the reaction conditions, varying the silk fibroin catalyst typology (SF or PSF) and loading, the solvent, the reaction time and temperature (**Table** **1**).

**Table 1:** Optimization of SF‐catalyzed model reaction between nitromethane and (*E*)‐chalcone 1a. Experimental conditions: Reaction performed with 1 mmol of 1a, 10 mmol of CH_3_NO_2_ and 2 mL of solvent at room temperature. Yields determined by H‐NMR.

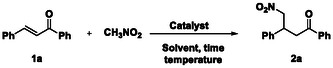				
Entry	Catalyst [mg]	Solvent	Time	**2a** (% Yield)
1	–	DMSO	12 h	traces
2	100	DMSO	12 h	70
3	100	DMSO	1 h	38
4	100	DMSO	30’	25
5	50	DMSO	1 h	35
6	25	DMSO	1 h	44
7	12	DMSO	1 h	45
8	5	DMSO	1 h	25
9a)	10	DMSO	1 h	52
10b)	10	DMSO	1 h	70
11b)	10	CH_3_NO_2_	1 h	0
12b)	10	H_2_O	1 h	16
13b)	10	H_2_O/DMSO 1:1 v/v	1 h	23
14b)	10	2‐Me‐THF	1 h	0
15b)	10	Diethyl carbonate	1 h	0
16b)	10	γ‐valerolactone	1 h	0
17b)	10	CH_3_CN	1 h	0
18b)	10c)	CH_3_CN	1 h	0
19b)	10c)	DMSO	1 h	99
20b)	10c)	DMSO	30’	80
21b)	5c)	DMSO	1 h	63

a) Reaction performed at 40 °C.

b) Reaction performed at 60 °C

c) Reaction performed with powdered silk fibroin (PSF).

The catalytic role of SF was confirmed by the lack of product formation in its absence (Table 1, entry 1), in line with the literature.^[^ ^31^ ^]^ Moreover, upon shortening the reaction time, a progressive drop of the product yield was observed, reaching 25% minimum yield for 30 min reaction time (Table 1, entries 2–4). One‐hour reaction time was set to investigate the catalyst loading. The minimal amount of catalyst needed to obtain maximum yield was assessed. The catalyst amount was gradually lowered with no significant yield loss in the 50–12 mg range, while suffering a significant loss when below that threshold. (Table 1, entries 4–8). Therefore, maximizing the catalyst turnover requires an optimized ratio of reactants referred to the total capability of the enzyme‐like catalytic pockets. We hypothesize that the catalytic domains are located in the hydrophilic region between the crystal domains of silk fibroin according to our previous studies on the Suzuki–Miyaura crosscoupling and Ullmann homocoupling reactions catalyzed by palladium supported SF.^[^ ^25^ ^]^

Regarding the temperature effect, the Michael addition of nitromethane to **1a** was performed at 40 °C and at 60 °C, observing that in both cases, the product is obtained in quantitative yield (99% in Table 1, entries 9 and 10).

The choice of the solvents largely affects the reaction outcome. Neat nitromethane, 2‐methyl tetrahydrofuran, water, diethyl carbonate, and *γ*‐valerolactone (Table 1, entries 11–16) led to insignificant amount of product, while yield slightly increased to 23% when a mixture of H_2_O and DMSO 1:1 vol/vol was used (Table 1, entry 13). Therefore, DMSO was found to be the best solvent among those investigated in our study (**Table** **2**, entry 10).

**Table 2:** Extension of the PSF catalyzed Michael addition to a series of (*E*)‐chalcone derivatives 1a–o.

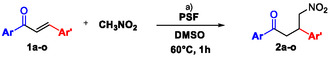			
Product	Yield	Product	Yield
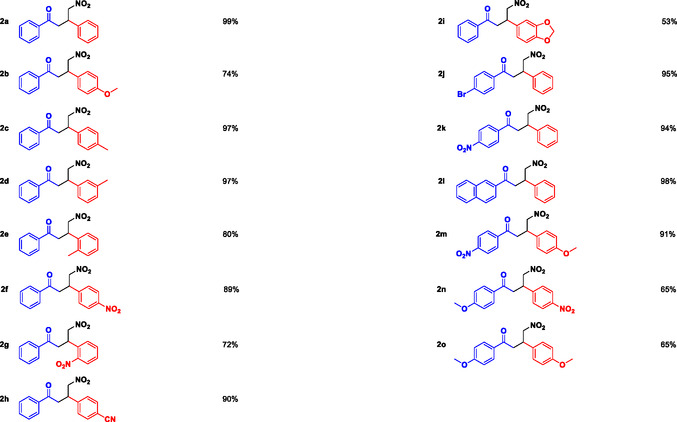			

a) Reactions performed with 2.5 mmol of **1a–o**, 25 mmol of CH_3_NO_2_, 2 mL of solvent and 25 mg PSF catalyst. Yields of pure products **2a–o** after column chromatography purification are reported.

We also explored the possibility to increase the catalyst available surface by milling the fibers with ZrO_2_ spheres in a mechanical oscillator at 36 Hz and evaluating the catalytic efficiency of the resulting powdered sample PSF versus its SF precursor. In fact, PSF was more effective than SF, leading to 99% yield (Table 1, entry 19) in one hour under the same experimental conditions (70% of the product when SF is used, Table 1, entry 10). To further explore the influence of SF type and solvent, the reaction was repeated using acetonitrile, a polar aprotic solvent analog. However, under these conditions, no product was obtained (Table 1, entry 18), this suggesting that DMSO likely plays a key role in the reaction mechanism.

The optimal reaction conditions were set at 60 °C, using DMSO as the solvent and PSF as the catalyst, with a reaction time of 1 h (Table 1, entry 19). This optimized protocol was then extended to different substrates bearing electron withdrawing or electron donating substituents on phenyl rings (Table 2). As shown in Table 2, good to excellent yields were observed for all the PSF catalyzed reactions without the need of complex purification procedures of the resulting adducts **2a–o**. Yields ranging from 80% to 97% were recorded for chalcone substrates with methyl substituents in different positions of the *β*‐phenyl ring (**2c–e**, Table 2). The reaction also occurred in fair to good yields in the presence of other electron‐donating groups such as the alkoxy substituents (**2i**: 53% and **2b**: 74% in Table 2) and in almost quantitative yields for substrates substituted with electron‐withdrawing —NO_2_ and —CN groups (**2f**: 89%, **2g**: 72%, and **2h**: 90%, Table 2).

Chalcones substituted on the ketone phenyl ring also showed excellent reactivity, as reported for products **2j**, **2k,** and **2l** whose yields were 95%, 94%, and 98%, respectively.

Moreover, multiple functionalization with —NO_2_ or —OCH_3_ groups on both phenyl rings was not detrimental to the PSF catalyzed process, leading to the resulting products in good yields (**2m**: 91%, **2n**: 65%, and **2o**: 65%): in particular, the presence of the electron‐withdrawing —NO_2_ and electron‐donating —OCH_3_ groups in the *para* positions of Ar and Ar’,respectively, enhance the reaction's conversion. This arrangement achieved higher efficiency compared to reversing the positions of the groups (91% for **2m** vs 70% for **2o** in Table 2).

### 2.3 PSF catalyzed Michael Addition of Nitromethane to Dibenzylideneacetones

With the aim to broaden the synthetic scope of the current PSF‐catalyzed reaction, we have also investigated the double Michael addition of nitromethane to a series of dibenzylideneacetone derivatives (**3p–w**). The results of the investigation on those substrates are summarized in **Table** **3**.

**Table 3:** PSF‐catalyzed Michael addition of nitromethane to dibenzylideneacetone derivatives 3p–w. Experimental conditions: Reaction performed with 2.5 mmol of 1, 50 mmol of CH_3_NO_2_, 4 mL of solvent and 25 mg of PSF catalyst. Yields after purification by column chromatography are reported in brackets.

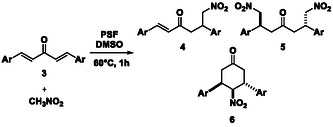						
Entry	**3** (Ar‐)		**4** (Yield%)	**5** (Yield%)	**6** (Yield%)	
1	**3p**	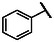	–	**5p** (41)	**6p** (27, 60)a)
2	**3q**	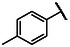	–	**5q** (40)	**6q** (57, 74)a)
3	**3r**	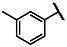	–	–	–
4	**3s**	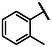	**4s** (80)	–	–
5	**3t**	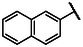	**4t** (49)	–	**6t** (22, 61)a)
6	**3u**	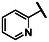	–	–	**6u** (25, 26)a)

a) Reactions performed with 1.0 equivalents of CH_3_NO_2_.

The reaction of dibenzylideneacetone **3p** with an excess of nitromethane provides the double Michael‐type adduct (**5p**, Table 3), with a significant (27%) amount of the product resulting from the intramolecular cyclization of the Michael monoadduct (**6p**, Table 3).

**Scheme 2:**
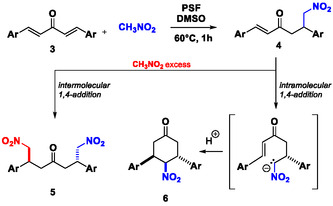
Products distribution depends on the amount of CH_3_NO_2_ used in the reaction medium.

Upon using one equivalent ratio of dibenzylideneacetone and nitromethane, the cyclization reaction is predominant, yielding **6p** as the sole product (60%). This output can be regarded as the effect of the relative orientation of the substrates and the Michael monoadduct intermediates into the protein catalytic sites. The use of a stoichiometric ratio of nitromethane rather than an excess is expected to favor an intramolecular cyclization of this intermediate assisted by the PSF catalyst, according to **Scheme** **2**. Indeed, the PSF site can deprotonate the *α* position to the —NO_2_ group in the monoadduct generating an *α‐*nitro alkyl carbanion acting as the nucleophile in the absence of the excess of nitromethane (Scheme 2).

Interestingly, both **5p** and **6p** derivatives were obtained with a high diastereoselectivity as anti‐isomers as confirmed by ^1^H‐NMR spectra. This evidence suggests the presence of steric constraints around active sites of catalyst.

Similar results were obtained using the substrate **3q** with *p‐*methyl substituted phenyl units: in this case, the cyclization product **6q** is predominant versus the double Michael adduct **5q** (57% vs 40%) even with an excess of nitromethane, and it is the sole product when the reagents were used in stoichiometric ratio (74% yield). Switching the ‐CH_3_ groups from the *‐para* to the *‐meta* position in **3r**, no reaction occurred, even with a large excess of nitromethane or prolonged reaction times, likely due to the bulkiness of the substrate. Moreover, in the case of the ‐*ortho* methyl substituted **3s**, the sole monoadduct **2s** bearing one double bond was obtained, likely due to the high steric hindrance of both the double and the cyclic adducts.

The catalyst selectivity seems to be affected also by other structural parameters, such as the rigidity and planarity of substrates: in fact, the substrate **3t** derived from naphthalene rings led to the mono adduct **4t**, as well as to the cyclic product **6t** in considerable amount.

Moreover, in the case of **3u** bearing two pyridyl rings instead of phenyl rings, only the cyclic product **6u** was obtained in low yield (25%) and no trace of other products was found even stopping the reaction at short times. This finding can be justified considering the electron‐withdrawing and weak basic effect of the nitrogen atoms of pyridyl rings that may inhibit the interaction of the substrate with the catalyst and may promote the intramolecular cyclization by a proximity effect to the —NO_2_ group of the monoadduct.

### 2.4 Enzyme‐like Catalysis by Silk Fibroin for the Michael reaction

The results discussed so far show that SF acts as an organocatalyst to promote Michael additions. This catalytic behavior can be explained based on its structural features. In fact, more than 70% SF consists of the alternation of 12 highly repetitive crystalline domains composed of the GAGAGS and GAGAGY hexapeptides, arranged in antiparallel‐ *β*‐sheet domains which further stack in crystal domains with its major axis parallel to the fiber elongation axis.^[^ ^32^ ^]^ The crystalline domains are linked together by eleven short amorphous and more polar regions. The strong packing of the *β*‐sheet domains renders inaccessible any possible site, thus the most favorable and plausible interaction site for SF is the hydrophilic linker.^[19a]^ Moreover, SF is rich in L‐glycine (46%) and L‐alanine (30%), two amino acids, whose sidechains, ‐H and —CH_3_ ,respectively, are unable to promote the Michael reaction. For this reason, we focused on the hydrophilic SF region. As observed by Patel et al.^[^ ^33^ ^]^ the amorphous and polar linker is a small oligopeptide composed by roughly 43 amino acids and, although not fully repeated in the whole protein structure, small and negligible variations in the primary sequence are encountered, without altering the structural features of SF. More specifically, the most abundant sequence is composed by the primary GAGAGAGAGAGTGSSGFGPYVAHGGYSGYEYAWSSESDFGTGS sequence, where the acid and basic amino acidic residues of the whole protein are located; in particular, the L‐glutamate (E) and L‐aspartate (D) residues can act as Brønsted bases (BB), being able to deprotonate the nitroalkane. The neutral polar L‐tyrosine (Y), L‐serine (S), L‐threonine (T) residues can act as Brønsted acids (BA), donating proton to the oxygen atom of the carbonyl group of the chalcone. All together these residues can cooperate to activate the substrates and promote the Michael reaction. Therefore, assuming that the hydrophilic region of SF can act as the catalytic portion, we refined the structural investigation of the hydrophilic linker used in a prior study by our group.^[^ ^25^ ^]^

In this work, an in silico investigation was carried out to suitably detail the molecular catalytic features of the SF protein. In this respect, the SiteMap algorithm was employed to detect putative binding sites based on the generation of molecular interaction fields (MIFs).^[^ ^34^ ^]^ Their type, strength, and direction are important to unveil the of specific interactions with molecular counterparts.

**Figure 2:**
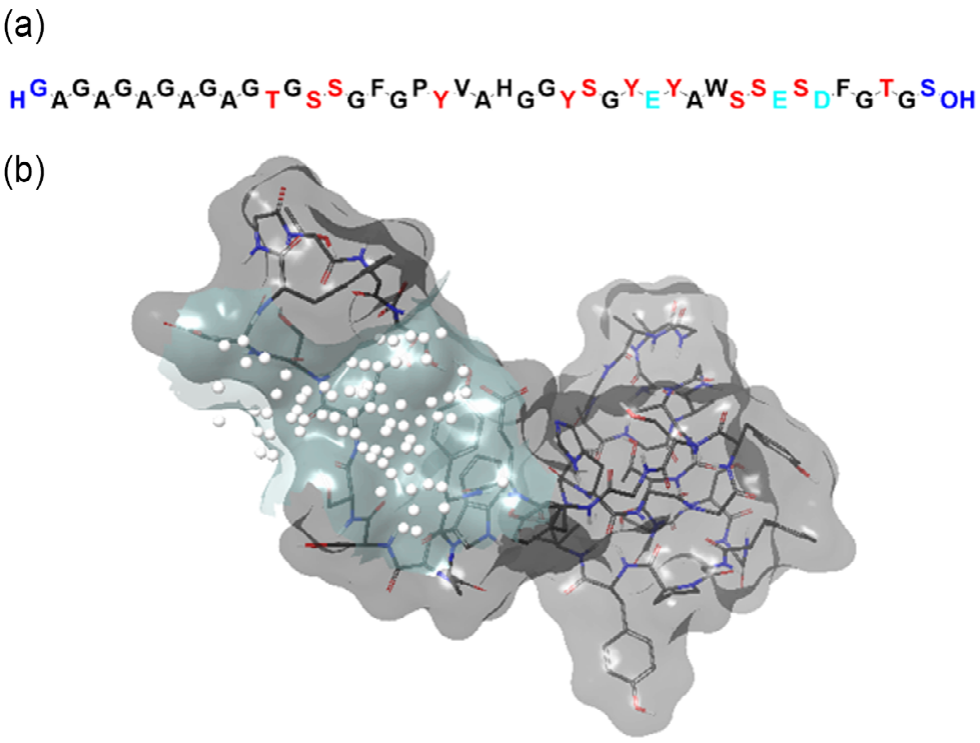
a) Amino acidic sequence of the SF segment likely involved in catalysis of Michael reactions. The N‐ and C‐ terminal are highlighted in blue, while the polar amino acids tyrosine (Y), serine (S), and threonine (T) are targeted in red. The acidic amino acids glutamate (E) and aspartate (D) are colored in cyan. b) The most likely main catalytic site identified by SiteMap algorithm. The white spheres are located at 1 Å on the grid and represent the site volume.

As highlighted in **Figure** **2**, one region was spotted as the most likely main catalytic site. It is formed by the following amino acids: Tyr‐29, Tyr‐31,Ser‐35, Glu‐36, Ser‐37, Asp‐38, and Phe‐39. For the sake of completeness, all the details are available in the Supporting Information (Table S2, Supporting Information).

**Figure 3:**
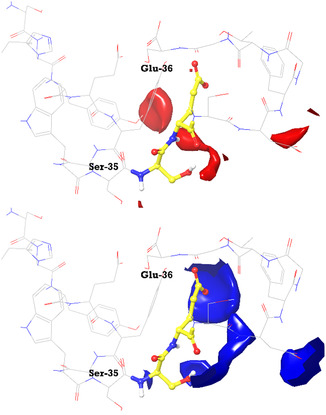
Hydrogen bond acceptor (red) and donor (blue) interaction maps in proximity of the Ser‐35/Glu‐36 pair.

The mapped binding site is rich in polar residues which can be engaged in hydrogen bond interactions and can be supposedly involved in promoting the Michael reaction. Interestingly, the MIF‐based analysis returned definite contours at a cut‐off value of −10 kcal/mol for the Ser‐35/Glu‐36 pair, which is supposed to behave as BA/BB switch (see **Figure** **3**). Noteworthy, the good solvent accessible surface areas of Ser‐35 and Glu‐36, equal to 65.97 Å^2^ and 29.73 Å^2^, respectively, confirm their key role.

Since the Michael reaction requires an acid‐base catalysis, it is reasonable that the polar amino acids of SF could be directly involved in the mechanism of catalysis, as observed for aldolases.^[^ ^35^ ^]^

As a support of our hypothesis, a natural enzyme (Rabbit muscle aldolase (RAMA)) was found to promote intramolecular nitroaldol reactions using exclusively acid‐base interaction sites through amino acid side groups.^[^ ^36^ ^]^

**Scheme 3:**
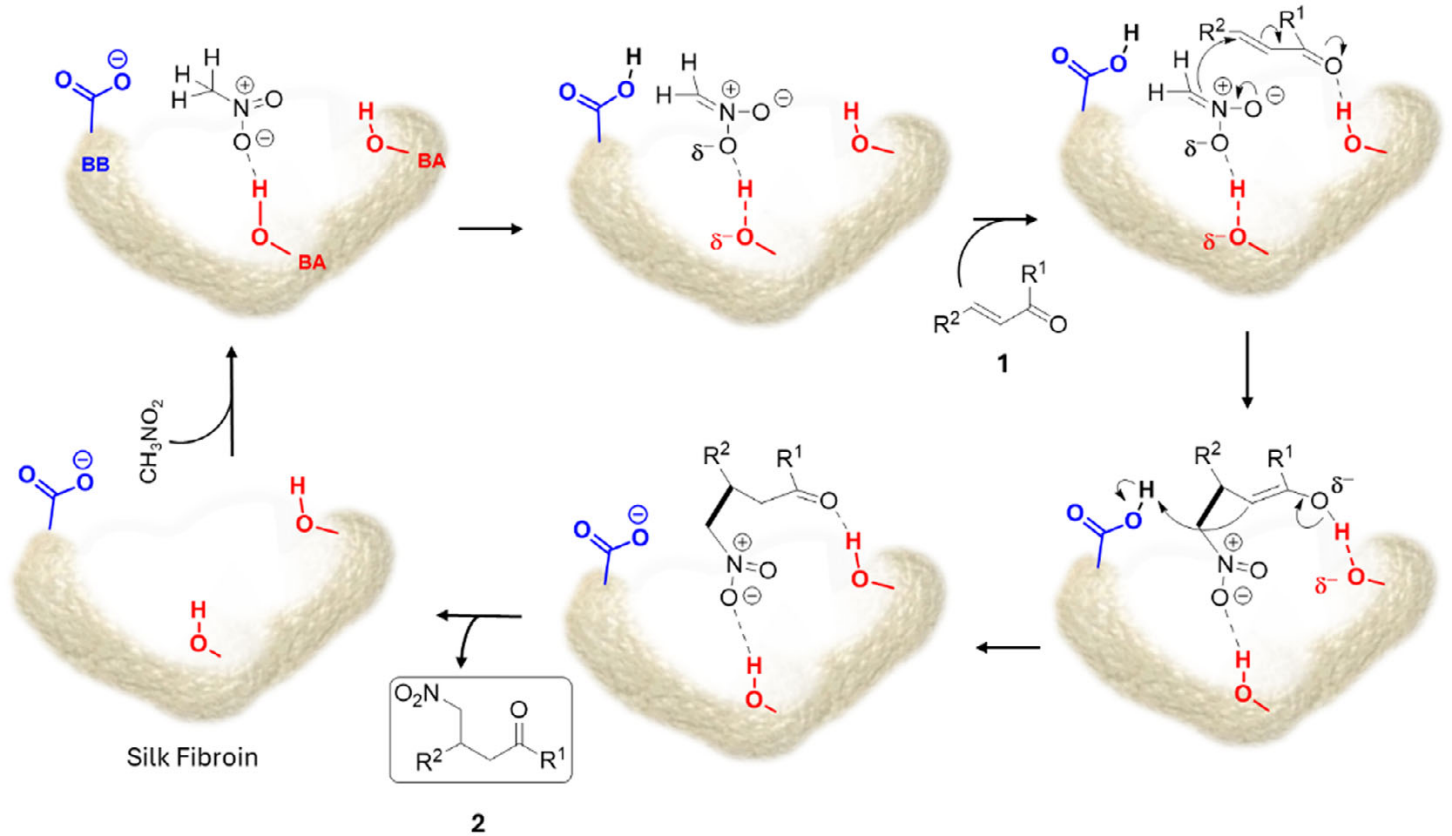
Proposed mechanism for the PSF‐catalyzed Michael addition of nitromethane to *α,β*‐unsaturated ketones **2**. (PSF: powdered Silk Fibroin, cyan shape, BB, BA)

Building on this in silico hypothesis, the previously obtained experimental data and the inhibition test (*vide infra*), we propose the reaction mechanism illustrated in **Scheme** **3**. The process initiates with the formation of a hydrogen bond between nitromethane and a hydroxylated amino acid (such as Ser, Tyr, or Thr) acting as a BA. Subsequently, a weak BB, such as glutamate or aspartate, is suggested to facilitate the deprotonation of nitromethane. Next, the carbon–carbon bond formation is expected to occur between the nitromethane anion and the enone derivative **2**, previously adsorbed onto the catalyst surface through a hydrogen bond interaction.

Finally, the enolic form of derivative **2** undergoes protonation by the carboxylic group, yielding the target *γ*‐nitroketone 2 and regenerating the catalyst PSF.

### 2.5 PSF catalyst Activity Inhibition

To confirm the role of polar groups of PSF hydrophilic regions, such as the —OH groups of serine, threonine, and tyrosine, they were protected via acetylation reaction using acetic anhydride, in accordance with a literature procedure.^[^ ^37^ ^]^ By changing the time and temperature of reaction, three different PSF‐A were prepared, namely PSF‐A 2.5%, PSF‐A 5%, and PSF‐A 8.6% based on the different degree of acetylation as detected by ATR‐FTIR spectroscopy (Figure S4, Supporting Information). The model reaction of chalcone **1a** with CH_3_NO_2_ was performed for each of the PSF‐As, comparing the results with those obtained with the PSF catalyst. Progressive acetylation of PSF was found to inhibit its catalytic efficiency, likely preventing the ability of —OH groups to coordinate and activate the substrates, with resulting reduction of yields. Compared to the quantitative conversion observed with PSF, PSF‐A 2.5%, PSF‐A 5%, and PSF‐A 8.6% catalysts led to 82%, 23%, and 16% of the product respectively. This evidence strengthens the hypothesis of a key role of the amino acidic ‐OH groups in the mechanism of Michael reaction promoted by PSF.

### 2.6 The Effect of the Solvent

As already reported in Table 1, the solvent plays a crucial role in the PSF‐catalyzed Michael reaction. In fact, DMSO was identified as the best reaction medium, since reactions occurred in good to quantitative yields in relatively short times. Chalcones are rapidly converted into the desired products in less than one hour at 60 °C.

To assess the role of DMSO as a solvent, we used DMSO/ethanol mixtures of different compositions (see Table S3, Supporting Information). Based on our hypothesis of the enzyme‐like catalytic pocket of SF, ethanol is expected to inhibit the SF catalyzed process, being able to actively form H‐bonds. The data show that a minimum amount of DMSO is necessary for the reaction to efficiently proceed. When the molar fraction of DMSO *χ*_DMSO_ is higher than 0.32, the reaction occurs quantitatively. On the contrary, for lower *χ*_DMSO_, the yields are significantly reduced by the progressive DMSO volume decrease. For *χ*_DMSO_ equal to zero (*i.e.,* reaction in neat EtOH), the chalcone does not react at all. We further investigated the effect of DMSO, by maintaining the *χ*_DMSO_ equal to 0.16 (yield 58%) and changing the amount of the catalyst: results revealed that there is a strict correlation between the DMSO and the catalyst amounts, since a lower amount of PSF catalyst (12 mg instead of 25 mg) does not affect yields (57%), whereas an increase of the catalyst to 100 mg led to quantitative yield. DMSO is a strong coordinating solvent but unable to donate H‐bonds, whilst EtOH and other protic solvents compete with the inner H‐bonding network of PSF that creates the catalytic pocket. When the solvent is progressively enriched in DMSO, the —OH groups can rearrange in intra‐protein bonds, reaching a critical condition when *χ*_DMSO_ is 0.16. A further suggestion of this mechanism is given by the variation of the ratio between PSF and DMSO.

### 2.7 Recyclability of the Catalyst

**Figure 4:**
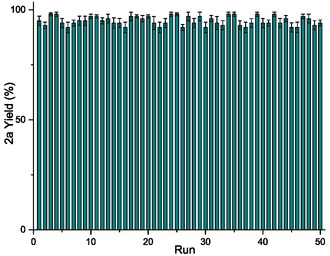
Recycling tests of PSF‐promoted Michael reaction, performed under the optimized conditions [1 mmol of chalcone **1a**, 10.0 mmol of CH_3_NO_2_, 50 mg of PSF, 2 mL of DMSO at 60 °C for 1 h]. Yields of **2a** product were calculated by ^1^H‐NMR analysis on the crude product.

The recyclability of the PSF catalyst in the Michael addition was evaluated through 50 consecutive reaction cycles. In each cycle, chalcone **1a** reacted with nitromethane and PSF in DMSO at 60 °C. After completing each reaction, the PSF catalyst was just washed with ethyl acetate, air‐dried and subsequently reused with fresh reagents. Noteworthy, the reaction consistently produced enone **2** in high yields (ranging from 92% to 99%) throughout all 50 cycles, without any noticeable decline in catalytic efficiency (**Figure** **4**). ATR‐FTIR analysis of the PSF catalyst before use and after 50 cycles (Figure S5, Supporting Information) showed the absence of significant structural changes in the biopolymer and confirming its chemical stability and suitability for repeated use.

## 3 Conclusion

In conclusion, the catalytic activity of *B. mori* SF towards the Michael reaction on several chalcones has been investigated, using both SF fibers and powder, with the latter catalytic form being the most effective due to the optimization of the surface/weight ratio. The catalyst promotes the Michael reaction in less than one hour, providing the addition product without side reactions, despite the relatively high temperatures (60 °C). The catalyst can be recycled up to 50 times without appreciable loss of activity.

An “enzyme‐like” action of the SF is envisaged, due to the existence of small SF hydrophilic regions SF located between the rigid *β*‐sheet crystal domains mainly composed of hydrophobic glycine and alanine residues. In fact, the hydrophilic segments are the sole regions of SF structure which are accessible to substrates. Polar amino acids (such threonine, serine, and tyrosine) are supposed to have a fundamental role in the creation of the SF catalytic cavities in which the reaction occurs and in actively cooperating to activate both reactants in the Michael reaction. In silico studies were carried out to spot a putative enzymatic pocket and to identify the amino acids relevant for catalysis. As a further confirmation of the key role played by —OH group of polar amino acids on the catalytic process, acetylation of SF to protect its —OH groups showed a detrimental effect on the Michael reaction, as an effect of their inhibition. Another confirmation of the key role of protein hydrogen bonding was given using protic solvents such as EtOH, able to compete with the hydrogen bond framework of the catalytic pocket, thus lowering the yield of the Michael addition or preventing it.

Overall, our study discloses the concept of using SF as an heterogeneous enzyme‐like organocatalyst for a C—C bond forming reaction. Its exceptional performances (high yields, complete diastereoselectivity) together with easy recovery of the products, very high number of recycling, robustness, and easy accessibility, make it interesting and worth of further investigation of generality for other organic reactions. The use of easily accessible and robust biopolymers for heterogeneous enzyme‐like catalysis of organic reactions can be foreseen as a general tool for sustainable organic synthesis.

## 4 Experimental Section

4.1

4.1.1

##### General Methods

All reagents were purchased from Sigma Aldrich and were used without any further purification. NMR spectra were recorded on a Agilent Technologies spectrometer at 500 and 125 MHz for ^1^H‐ and ^13^C‐nuclei, respectively. Chemical shifts are reported in part per million (δ). ATR‐FTIR spectra were acquired with a PerkinElmer Spectrum Two spectrophotometer equipped with A 2 × 2 mm Diamond crystal. Spectra were recorded in the range 4000–400 cm^−1^ with a 2 cm^−1^ resolution, using 0.25 cm^−1^ acquisition interval and acquiring 32 scans for each sample. SEM analyzes were carried out by a VP field emission SEM EDS Zeiss Sigma 300 equipped with an in lens backscattered and secondary electron detectors. An accelerating voltage of 7 kV was used and 5 mm working distance. Field emission scanning electron miscroscope (FE‐SEM) samples were placed onto stainless steel sample holders with carbon tape. A gold sputtering was performed onto samples before analyzes in order to prevent electron charging due to the low sample conductivity, thus enhancing topography imaging. The EDS detector was an X‐Max Silicon Drift Detector, Oxford Instruments NanoAnalysis. The sample was prepared by depositing ethanol dispersions onto aluminum stubs coated with carbon tape. The acquired images were analyzed with AZtec Software. The SF hydrophilic structure was refined by employing the Protein Preparation Wizard tool (Schrodinger Suite, version 2024‐1). The Force Fields OPLS4 was employed to mitigate steric clashes. The potential binding site was identified and characterized by using SiteMap, a tool available from the Schrödinger Suite molecular modeling package (version 2024‐1).^[^ ^38^ ^]^ Default parameters were set to detect shallow binding sites, including a standard grid with grid spacing equal to 0.7 Å. The Van der Waals and distance‐dependent electrostatic interactions were probed at each grid point to generate hydrophobic and hydrophilic potentials. Threshold values were thus set to contour the corresponding hydrophobic and hydrophilic maps. The hydrophilic maps were further divided into donor or acceptor regions. Solvent accessible surface areas (SASA) were computed by using the POPScomp (Parameter OPtimzed Surfaces) algorithm (version 3.2.2), free available at http://popscomp.org:3838.

##### Preparation of Degummed SF

Degummed SF was obtained from *B. mori* cocoons purchased from Tajima Shoji (Japan). To remove sericin, the cocoons were cut into fourths, shredded and boiled for 30 min in an aqueous solution of Na_2_CO_3_ 0.02 M, then rinsed thoroughly with bidistilled water to remove the residual sericin and the excess of salt. The fibers were dried at ambient conditions for 24 h.

##### Preparation of PSF

PSF was prepared by mechanical pulverization of SF using a Mixer Mill IST636, with ZrO_2_ Yttrium doped jars and balls, using eight spheres (Ø 7 mm), operating at 36 Hz for 30 min. Powder was characterized through ATR‐FTIR spectroscopy and SEM for particulate morphology and dimensions.

##### General Procedure for Michael Addition Reaction

In a screwed glass vial, the following components were added in order: 1 equivalent of substrate (chalcone or DBA) and 2 mL of DMSO. After complete dissolution, the catalyst PSF and CH_3_NO_2_ (10 equivalents for chalcone substrates or 20 equivalents in the case of DBAs) were added, and the reaction was let to proceed for a certain time. Upon completion, the reaction mixture was filtered through a funnel to remove the catalyst. Brine water was added to the liquors, and the liquid phases were extracted in a separatory funnel with ethyl acetate. The organic phases were collected, dried over Na_2_SO_4_, filtrated through a cotton‐filled funnel, and then evaporated under reduced pressure. The crude products, if needed, were purified through column chromatographic. The final purified products were characterized by ^1^H‐, ^13^CNMR, and melting point determination.

## Conflict of Interest

The authors declare no conflict of interest.

## Supporting information

Supplementary Material

## Data Availability

The data that support the findings of this study are available in the supplementary material of this article.
