# Supplementary material for: Bombyx Mori Silk Fibroin as a Sustainable Organocatalyst for Diastereoselective Michael Additions
Source: ChemSusChem. 2025 Jul 14;18(16):e202500584. doi: 10.1002/cssc.202500584 (PMC12330330; doi:10.1002/cssc.202500584)
Supplement: Supplementary file 1 — Supplementary Material [file CSSC-18-e202500584-s001.pdf]

Supporting Information

Bombyx Mori Silk Fibroin as a Sustainable Organocatalyst for Diastereoselective Michael Additions

Carola Ricciardelli†, [a] Giorgio Rizzo†, [a] Pietro Cotugno*, [a] Antonio Salomone, [a] Daniela Trisciuzzi, [b] Orazio Nicolotti, [b] Erica Colaprico, [a] Cosimo D. Altomare*, [b]

and Gianluca M. Farinola [a]

[a] Dr. C.Ricciardelli, Dr. G. Rizzo, Prof. P. Cotugno, Prof. A. Salomone, Ms. E. Colaprico, Prof. G. M. Farinola
Department of Chemistry, University of Bari Aldo Moro, Via E. Orabona 4, 70125 Bari, Italy
E-mail: pietro,cotugno@uniba.it

[b] Dr. D.Trisciuzzi, Prof. O. Nicolotti, Prof. C.D. Altomare
Department of Pharmacy-Pharmaceutical Sciences, University of Bari Aldo Moro, Via E. Orabona 4, 70125 Bari, Italy
E-mail: cosimodamiano.altomare@uniba.it

† These authors contributed equally to this work.

Experimental Section

General Information

Silk fibroin (SF) was obtained from *Bombyx mori* cocoons from Tajima Shoji (Japan). All the chemicals were purchased from commercial sources and used as received without purification. Column chromatographies were performed with Fluka silica gel, pore size 60 Å, 70-230 mesh, 63-200 µm. ^{1}H-NMR and ^{13}C-NMR spectra were recorded at room temperature in deuterated solvents solution with an Agilent 500 spectrometer, operating at a frequency of 500 MHz for ^{1}H, 125 MHz for ^{13}C; chemical shifts (δ) values are given in parts per million (ppm) and coupling constants (J) in Hertz. Melting points were determined with a Kohfler melting points apparatus.
Scanning Electron Microscopy (SEM) analyses of degummed SF and powdered SF were performed with a VP Field emission SEM EDS Zeiss Sigma 300 equipped with an in lens backscattered and secondary electron detectors. An accelerating voltage of 7 kV was used and 5 mm working distance. SEM samples were placed onto stainless-steel sample holders with carbon tape. A gold sputtering was performed onto samples before analyses in order to prevent electron charging due to the low sample conductivity, thus enhancing topography imaging. The acquired images were analyzed with Aztec Software. The EDS Energy Dispersive X-ray Spectroscopy) detector was an X-Max - Silicon Drift Detector (SSD) – Nanoanalysis – Oxford Instruments. The sample was prepared by depositing ethanol dispersions onto aluminum stubs coated with carbon tape. The acquired images were analyzed with Aztec Software.
ATR-FTIR spectra were acquired with a Perkin Elmer Spectrum Two Spectrophotometer equipped with a 2x2 mm Diamond crystal. Spectra were recorded in the range 4000 - 400 cm^{-1} with a 2 cm^{-1} resolution, using 0.25 cm^{-1} acquisition interval and acquiring 32 scans for each sample.

Preparation of the catalyst

Raw silk cocoons (5.00 g) were shredded in 1 cm pieces and boiled in a 0.02 M Na_2CO_3 aqueous solution (2L) at 100°C for 30 minutes under constant stirring to remove the external sericine coating. Degummed silk, called silk fibroin (SF) is the true protein part of cocoons and was repeatedly washed in bidistilled water to remove the salts and the sericines extracted. The final SF was air-dried for 24 hours, obtaining 3.75 g of pure SF.[1]

Powdered Silk Fibroin (PSF) was prepared by mechanical pulverization using a Mixer Mill IST636, with ZrO_2 Yttrium doped jars and balls, using 8 spheres (Ø 7 mm), operating at 36 Hz for 30 minutes. Powder was characterized through ATR-FTIR spectroscopy and SEM for particulate morphology and dimensions.

ATR-FTIR characterization

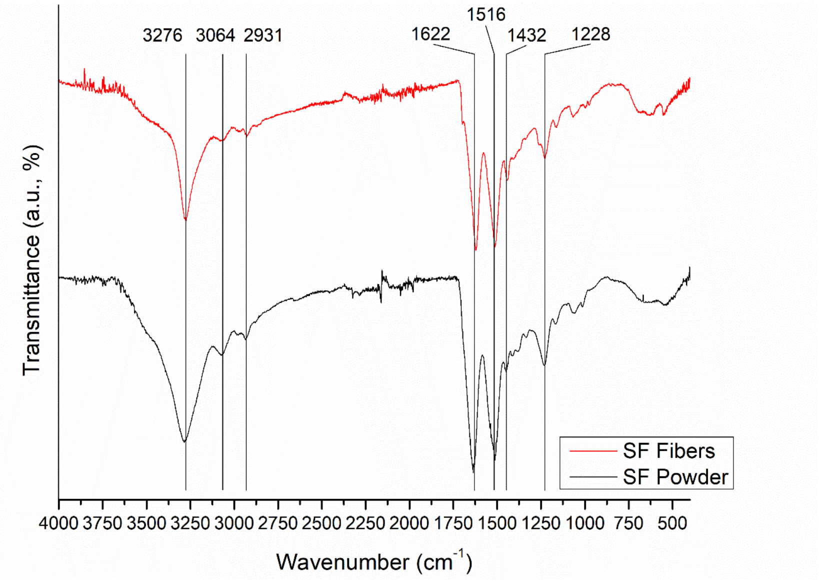

Figure S1: ATR-FTIR Spectra of degummed SF fibers and powder SF. Black lines highlight the presence of typical SF bands and relative wavenumbers.

SEM - EDS characterization

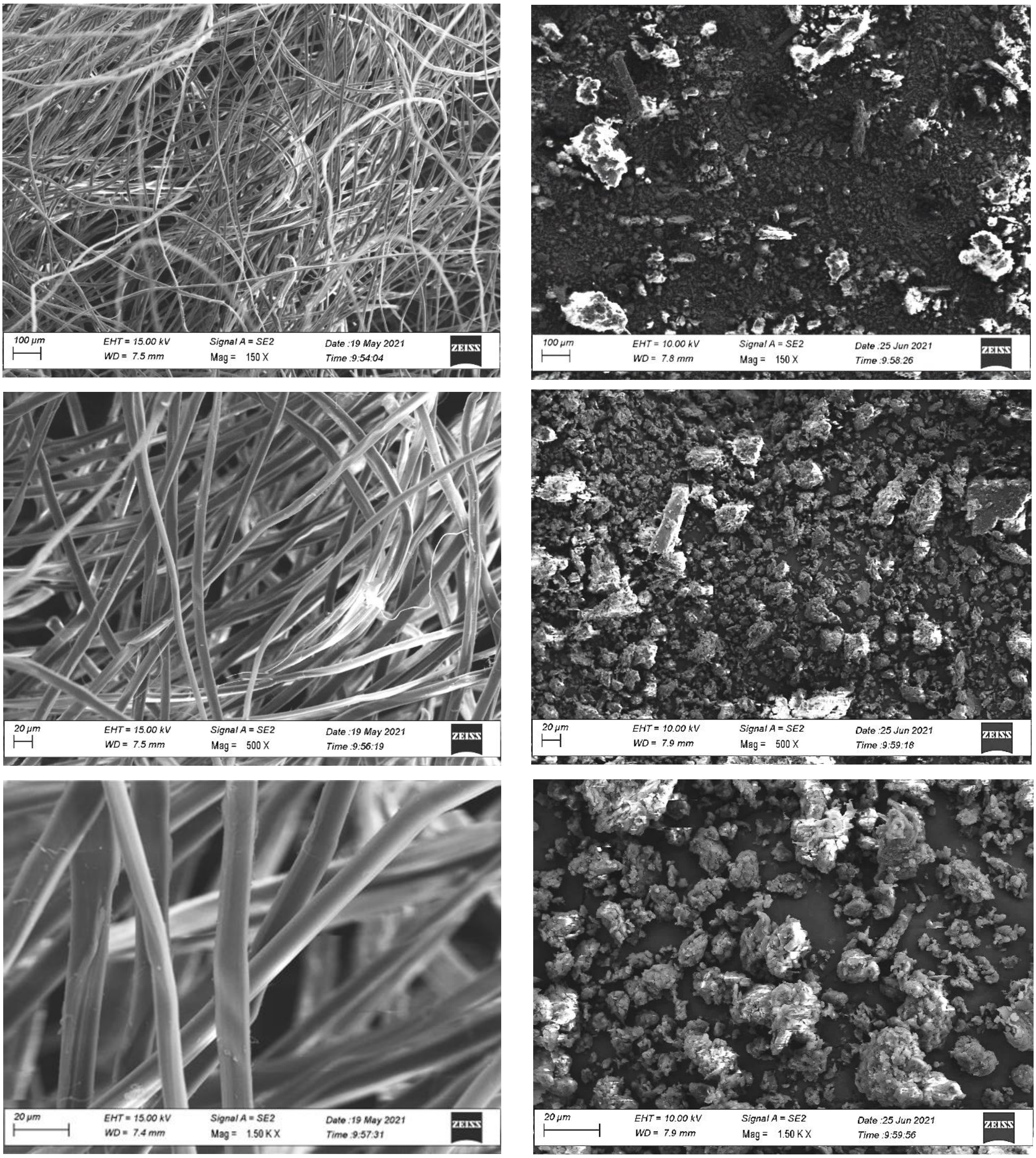

Figure S2: SEM images of degummed SF (on the right) and powder SF (on the left) at three different magnifications.

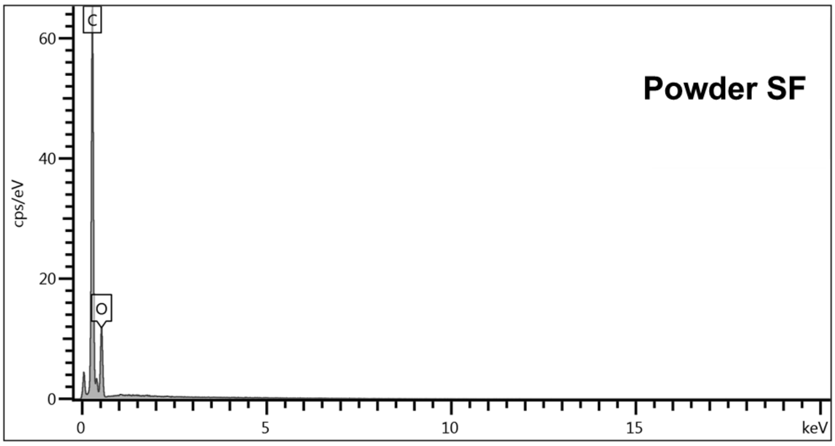

Figure S3: EDS spectra and peak assignment. Lower table reports weight and atomic percentages of atoms in the sample.

Table S 1: Elemental composition of PSF by EDS spectroscopy.

Element	Wt%	Wt% Sigma
C	56.69	0.29
O	43.31	0.29
Total:	100	

In silico mapping of putative catalytic cavities

The SF hydrophilic structure was refined by employing the Protein Preparation Wizard tool (Schrodinger Suite version 2024-1). According to Singh *et al.*,[2] the 42-based amino acid peptide GAGAGAGAGAGTGSSGFGPYVAHGGYSGYEYAWSSESDFGTGS, (where G = glycine, A = alanine, T = threonine, S = serine, F = phenylalanine, P = proline, Y = tyrosine, V = valine, H = histidine, E = glutamic acid, W = tryptophan, D = aspartic acid) was used as a model to mime hydrophilic and amorphous region of SF. The Force Fields OPLS-4 was employed to mitigate steric clashes. The potential binding site was identified and characterized by using SiteMap, a tool available from the Schrödinger Suite molecular modeling package (version 2024-1). Default parameters were set to detect shallow binding sites, including a standard grid with grid spacing equal to 0.7Å. The van der Waals and distance-dependent electrostatic interactions were probed at each grid point to generate hydrophobic and hydrophilic potentials. Threshold values were thus set to contour the corresponding hydrophobic and hydrophilic maps. The hydrophilic maps were further divided into donor or acceptor regions. Solvent accessible surface areas (SASA) were computed by using the POPScomp (Parameter OPtimsed Surfaces) algorithm (version 3.2.2), free available at http://popscomp.org:3838/4.

Table S 2: Structural and energetic features on the SF binding site detected by the SiteMap algorithm.

Pocket surface	504.255A^2
Hydrophobic residues	0.130
Hydrophilic residues	0.846
Balance Hydrophobic/ Hydrophilic	0.154
Hydrophobic surface (-0.75kcal/mol)	94.068 A^2
HB acceptor surface (-8kcal/mol)	83.469 A^2
HB donor surface (-8kcal/mol)	309.391 A^2

Study of the inhibition of the active site

In order to study the effect of the side-chain groups of the aminoacids in SF on the activation of the Michael reaction, PSF was progressively acetylated according to a literature protocol.[3] Acetylation with acetic

anhydride ensured the progressive esterification of the hydroxyl groups of serine, threonine, and tyrosine, thus preventing any coordinating and activating effect of these functionalities towards the substrates. In principle, also amidation is possible, but the amount of side-chain amines in SF (lysine 0.2 mol%, arginine 0.3 mol%, histidine 0.1 mol%) is negligible. Three different grades of acetylation were achieved, namely 2.5%, 5.0% and 8.6% by changing both reaction times and temperatures. Acetylation was confirmed by ATR-FTIR spectroscopy on final catalysts by disappearing of -OH stretching band and consecutive appearing of the ester carbonyl stretching band. The Acetylated catalyst was dried in oven at 60°C and used for the model reaction of (E)-chalcone (2.5 mmol) and CH_3NO_2 (25 mmol) in 2 mL of DMSO, using 25 mg of acetylated PSF. Work-up of the reaction followed a typical extraction protocol reported in the general procedure. Conversion of the chalcone was assessed by ^{1}H-NMR spectroscopy.

2.5% Acetylated PSF: In a 100 mL round bottom flask were introduced 5 mL of acetic anhydride, 3 mL of triethylamine, and 100 mg of PSF. The reaction was let to proceed at 60°C for 4 hours. The suspension progressively darkened and the PSF turned yellow. After completion, the mixture was let to cool to room temperature, poured in cold water and stirred to remove acetic anhydride and triethylamine. The acetylated catalyst was recovered by decantation and washed other three times in fresh cold water and then three times in methanol. Conversion of (E)-chalcone was 82%.

5.0% Acetylated PSF: In a 100 mL round bottom flask were introduced 10 mL of acetic anhydride, 2 mL of triethylamine, and 100 mg of PFS. The reaction was heated to reflux (about 100°C) for 1 hour. Catalyst isolation, purification and model reaction procedure was the same as for 2.5% acetylated PSF previously described. Conversion of (E)-chalcone was 23%.

8.6% Acetylated PSF: In a 100 mL round bottom flask were introduced 10 mL of acetic anhydride, 1 mL of triethylamine, and 200 mg of PFS. The reaction was heated to reflux (about 140°C) for 7 hours. Catalyst isolation, purification and model reaction procedure was the same as for 2.5% acetylated PSF previously described. Conversion of (E)-chalcone was 16%.

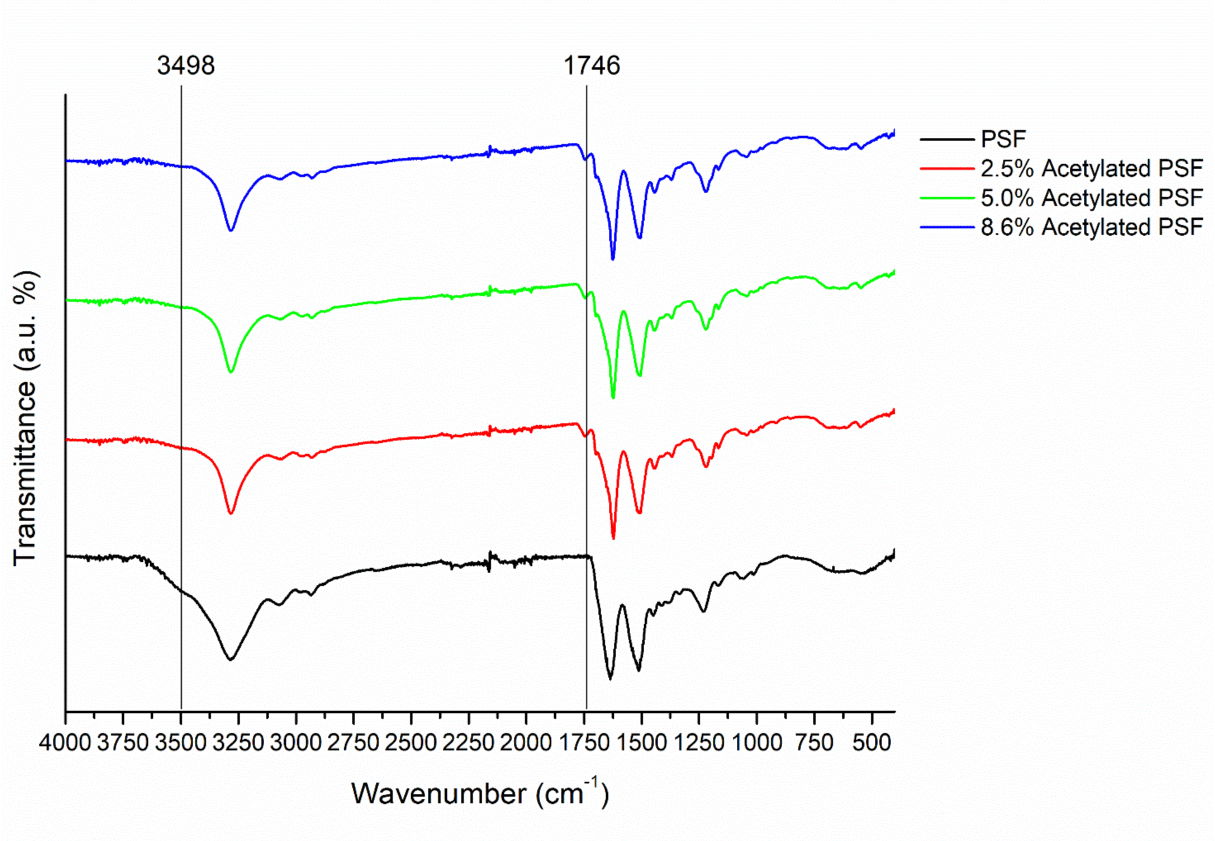

Figure S4: Stacked ATR-FTIR spectra of progressively acetylated PFS catalysts. Are highlighted the bands of -OH and ester C=O stretching frequencies at 3498 and 1746 cm-1, respectively.

Study on the effect of DMSO

To evaluate the beneficial effect of DMSO in the Michael reaction, a binary system composed of DMSO and EtOH was set as the model solvent for aldol reaction (E)-chalcone. EtOH was selected as the inefficient solvent since the reaction does not proceed at all when this solvent is used. Moreover, it can effectively donate H-bonds that compete with the inner H-bonding texture of SF. Calculations were performed on the basis of the molar fraction χ of each solvent, using 1.25 mmol of (E)-chalcone, 12.5 mmol of CH_3NO_2, 25 mg of PFS at 60°C for 1 hour. The amount of solvents used is ca. 2 mL. Yields were determined by ^{1}H-NMR. To study the effect of DMSO in terms of catalyst quantity, other two reactions were performed using half (12.5 mg) and double amount of PSF (50 mg) and maintaining the χ_{DSMO} = 0.16 as the reference reaction.

Table S 3: Study on the effect of DMSO in the Michael addition reaction.

Entry	χ_{DMSO}	V_{DMSO} (mL)	χ_{EtOH}	V_{EtOH} (mL)	Yield %[a]
1	1.00	2.2	0.00	0.00	100
2	0.96	2.13	0.04	0.07	100
3	0.80	1.78	0.20	0.35	100
4	0.64	1.42	0.36	0.63	100
5	0.48	1.07	0.52	0.91	100
6	0.32	0.71	0.68	1.19	100
7	0.16	0.36	0.84	1.47	56
8	0.12	0.27	0.86	1.51	26
9	0.08	0.18	0.92	1.61	20
10	0.04	0.09	0.96	1.68	16
11	0	0.00	1.00	1.76	0
12[b]	0.16	0.36	0.84	1.47	57
13[c]	0.16	0.36	0.84	1.47	100

a: Yields determined by ^{1}H-NMR.

b: PSF catalyst amount halved to 12.5 mg

c: PSF catalyst amount doubled to 50 mg

Recycling of the catalyst

The recyclability of the SF catalyst was tested by repeating 50 times the model reaction of (*E*)-chalcone (208 mg, 1.0 mmol) with nitromethane (540 µL, 10.0 mmol) and 50 mg of PSF in 2 mL of DMSO at 60 °C for 1 hour. In a 20 mL screw glass vial, the reagents were dissolved in a suspension of DMSO and PSF in the following order: chalcone and nitromethane. After the addition of CH_3NO_2, the reaction time was monitored, and the reaction was quenched after 1 hour. Quenching was made by addition of ethyl acetate (5 mL) and by centrifugation of the mixture for three times (2500 rpm, 2 minutes each time). The organic layers were collected and then extracted with bidistilled water (10 mL) in a separatory funnel. The organic phase was dried over anhydrous Na_2SO_4 and the solvent was removed under vacuum. The PSF was dried with hot air and reused for the next cycle without any further treatment, using fresh reagents and solvents for each cycle. The reagent conversion was calculated with respect to the starting chalcone by ^{1}H-NMR spectroscopic analysis.

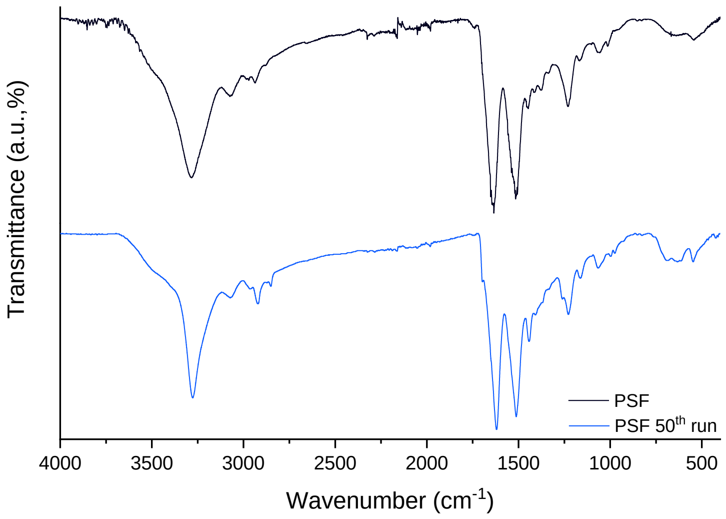

Figure S5: ATR-FTIR Spectra of Powder SF before any catalytic run and PSF catalyst after 50 recycling runs.

Synthesis of precursors (chalcones and DBAs)

All the chalcones and DBAs were synthesized via a Claisen-Schmidt aldol condensation reaction involving a proper aldehyde and ketone as starting materials, according to the literature.[4] The aldehyde and the ketone were mixed in ethanol in an ice bath, followed by a slow drop-addition of 0.6 M NaOH solution to the flask. After the complete addition of the base, the reaction was heated to room temperature and let to proceed for 2 hours under continuous and vigorous stirring. After completion, all the products were collected by precipitation and filtration on a Buchner funnel, washed with water and cold ethanol. The pure compounds were obtained by crystallization from hot ethanol solutions. Physical and spectroscopic data were all in accordance with the literature references.

General procedure for Michael reaction

In a 10 mL screwed glass vial, in the following order, were added 1 equivalent (2.5 mmol) of substrate (chalcone or DBA) and 2 mL of DMSO. After complete dissolution with magnetic stirring, the catalyst based on SF (fibers or powder) were added and let soaked and fully submerged in DMSO solution. After 5 min of equilibration at room temperature or at a selected temperature, CH_3NO_2 (10 equivalents for chalcone substrates or 20 equivalents in the case of DBAs, 1 equivalent for 3p,3q, 3t, 3u substrates) were injected and the reaction was let to proceed for a certain time. After completion, the reaction mixture was filtered through Buchner funnel to remove the catalyst. SF scaffolds were repeatedly washed with bidistilled water and then with ethyl acetate. Brine water (20 mL) was added to the liquors, and the liquid phases were then extracted in a separatory funnel with ethyl acetate (3x10 mL). The organic phases were collected, dried over Na_2SO_4, filtrated through cotton funnel and the filtrate was then dried under reduced pressure. The crude products, if needed, were purified through chromatographic columns, using different mixtures of organic solvents depending on the polarity of each compound. Final purified products were characterized by ^{1}H-, ^{13}C-NMR, and melting points.

Synthesis of 4-nitro-1,3-diphenylbutan-1-one (2a)

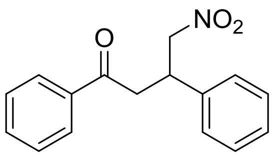

Following the general procedure described in the general conditions, 2.5 mmol of chalcone were dissolved in 2 mL of DMSO. After dissolution, 25 mg of powder silk fibroin were added and 25 mmol of CH_3NO_2 were injected. The reaction was let to proceed at 60°C for 1 hours. The ^{1}H-NMR spectroscopic analysis of crude product revealed a quantitative conversion, without the needing of chromatography purification. Isolated yield 99%.

White crystals. M.p.: 86-91°C (lit. 84-85°C[21]).

^{1}H-NMR (500 MHz, $CDCl_3$), δ (ppm): 7.92 (d, *J* = 7.4 Hz, 2H); 7.62-7.54 (m, 1H); 7.46 (t, *J* = 7.8 Hz, 2H); 7.34 (m, 2H); 7.28 (m, 3H); 4.83 (dd, *J* = 12.5, 6.5 Hz, 1H); 4.69 (dd, *J* = 12.5 Hz, 8.1 Hz, 1H); 4.23 (bquintuplet, *J* ~ 7 Hz, 1H); 3.54-3-38 (m, 2H).

^{13}C-NMR (125 MHz, $CDCl_3$), δ (ppm): 196.97; 139.25; 136.49; 133.71; 129.21; 128.87; 128.15; 128.01; 127.58; 79.69; 41.65; 39.41.

Synthesis of 3-(4-methoxyphenyl)-4-nitro-1-phenylbutan-1-one (2b)

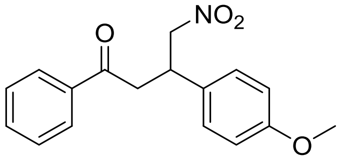

Following the general procedure described in the general conditions, 2.5 mmol of chalcone were dissolved in 2 mL of DMSO. After dissolution, 25 mg of powder silk fibroin were added and 25 mmol of CH_3NO_2 were injected. The reaction was let to proceed at 60°C for 1 hours. The crude product was purified through column chromatography (SiO_2, *n*-hexane/AcOEt 8:2). Isolated yield 74%.

White solid. M.p.: 60-63 °C (lit. 62-63 °C[5]).

^{1}H-NMR (500 MHz, $CDCl_3$), δ (ppm): 7.94-7.88 (m, 2H); 7.57 (t, *J*=7.4 Hz, 1H); 7.44 (t, *J*=7.7 Hz, 2H); 7.19 (d, *J*=8.6 Hz, 2H); 6.84 (d, *J*=8.6 Hz, 2H); 4.78 (dd, *J*=12.4, 6.5 Hz, 1H); 4.63 (dd, *J*=12.4, 8.2 Hz, 1H); 4.21-4.13 (m, 1H); 3.76 (s, 3H), 3.49-3.35 (m, 2H).

^{13}C-NMR (125 MHz, $CDCl_3$), δ (ppm): 197.08; 159.13; 136.47; 133.60; 131.08; 128.78; 128.59; 128.09; 114.47; 79.91; 55.30; 41.71; 38.69.

Synthesis of 4-nitro-1-phenyl-3-(p-tolyl)butan-1-one (2c)

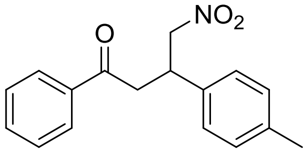

Following the general procedure described in the general conditions, 2.5 mmol of chalcone were dissolved in 2 mL of DMSO. After dissolution, 25 mg of powder silk fibroin were added and 25 mmol of CH_3NO_2 were injected. The reaction was let to proceed at 60°C for 1 hours. The ^{1}H-NMR spectroscopic analysis of crude product revealed a quantitative conversion, without the needing of chromatography purification. Isolated yield 97%.

Pale yellow crystals. M.p. 75-78°C (lit. 74-75 °C[6])

^{1}H-NMR (500 MHz, $CDCl_3$), δ (ppm): 7.87 (d, *J*=7.2 Hz, 2H); 7.52 (t, *J*=7.4 Hz, 1H); 7.40 (t, *J*=7.8 Hz, 2H); 7.14 (d, *J*=8.1 Hz, 2H); 7.09 (d, *J*=8.1 Hz, 2H); 4.77 (dd, *J*=12.5, 6.5 Hz, 1H); 4.62 (dd, *J*=12.5, 8.3 Hz, 1H); 4.21-4.10 (m, 1H); 3.42 (dd, *J*=17.7, 6.5 Hz, 1H); 3.36 (dd, *J*=17.7, 7.4 Hz, 1H); 2.26 (s, 3H).

^{13}C-NMR (125 MHz, $CDCl_3$), δ (ppm): 196.93; 137.39; 136.36; 136.12; 133.46; 129.63; 128.66; 127.95; 127.28; 79.68; 41.54; 38.91; 20.98.

Synthesis of 4-nitro-1-phenyl-3-(m-tolyl)butan-1-one (2d)

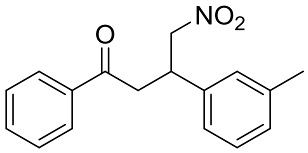

Following the general procedure described in the general conditions, 2.5 mmol of chalcone were dissolved in 2 mL of DMSO. After dissolution, 25 mg of powder silk fibroin were added and 25 mmol of CH_3NO_2 were injected. The reaction was let to proceed at 60°C for 1 hours. The ^{1}H-NMR spectroscopic analysis of crude product revealed a quantitative conversion, without the needing of chromatography purification. Isolated yield 97%.

Pale yellow oil

^{1}H-NMR (500 MHz, $CDCl_3$), δ (ppm): 7.85 (d, *J*=7.3 Hz, 2H); 7.49 (t, *J*=7.3 Hz, 1H); 7.37 (t, *J*=7.7 Hz, 2H); 7.14 (t, *J*= 7.6 Hz, 1H); 7.06-6.98 (m, 3H); 4.75 (dd, *J*=12.6, 6.4 Hz, 1H); 4.61 (dd, *J*=12.6, 8.4 Hz, 1H); 4.18-4.06 (m, 1H); 3.40 (dd, *J*=17.8, 6.4 Hz, 1H); 3.34 (dd, *J*=17.8, 7.5 Hz, 1H); 2.49 (DMSO); 2.25 (s, 3H).

^{13}C-NMR (125 MHz, $CDCl_3$), δ (ppm): 196.83; 139.10; 138.44; 136.24; 133.35; 128.70; 128.55; 128.39; 128.13; 127.84; 124.24; 79.46; 41.43; 40.70 (DMSO); 39.07; 21.24.

Synthesis of 4-nitro-1-phenyl-3-(o-tolyl)butan-1-one (2e)

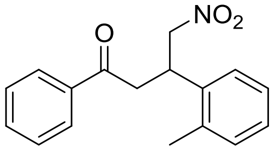

Following the general procedure described in the general conditions, 2.5 mmol of chalcone were dissolved in 2 mL of DMSO. After dissolution, 25 mg of powder silk fibroin were added and 25 mmol of CH_3NO_2 were injected. The reaction was let to proceed at 60°C for 1 hours. The crude product was purified through column chromatography (SiO_2, *n*-hexane/AcOEt 7:3). Isolated yield 80%.

Yellow oil.

^{1}H-NMR (500 MHz, $CDCl_3$), δ (ppm): 7.92 (dd, *J*=8.3, 1.1 Hz, 2H); 7.57 (t, *J*=7.4 Hz, 1H); 7.45 (t, *J*=7.8 Hz, 2H); 7.22-7.13 (m, 4H); 4.79 (dd, *J*=12.5, 7.0 Hz, 1H); 4.66 (dd, *J*=12.5, 7.8 Hz, 1H); 4.58-4.52 (m, 1H); 3.48 (dd, *J*=17.8, 6.5 Hz, 1H); 3.39 (dd, *J*=17.8, 7.3 Hz, 1H); 2.48 (s, 3H).

^{13}C-NMR (125 MHz, $CDCl_3$), δ (ppm): 197.01; 137.45; 136.49; 136.39; 133.56; 131.20; 128.76; 128.03; 127.52; 126.63; 125.45; 79.08; 41.62; 34.39; 19.60.

Synthesis of 4-nitro-3-(4-nitrophenyl)-1-phenylbutan-1-one (2f)

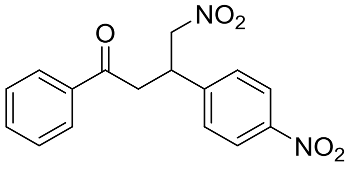

Following the general procedure described in the general conditions, 2.5 mmol of chalcone were dissolved in 2 mL of DMSO. After dissolution, 25 mg of powder silk fibroin were added and 25 mmol of CH_3NO_2 were injected. The reaction was let to proceed at 60°C for 1 hours. The ^{1}H-NMR spectroscopic analysis of crude

product revealed a complete conversion, without the needing of chromatography purification. Isolated yield 89%.

White crystals. M.p.: 95-98°C (lit. 91-94 °C[7])

^{1}H-NMR (500 MHz, $CDCl_3$), δ (ppm): 8.20-8.18 (m, 2H); 7.92-7.90 (m, 2H); 7.61-7.58 (m, 1H) 7.51-7.45 (m, 4H); 4.88 (dd, *J*=12.9, 6.1 Hz, 1H); 4.74 (dd, *J*=12.9, 8.5 Hz, 1H); 4.40-4.31 (m, 1H); 3.52 (m, 2H).

^{13}C-NMR (125 MHz, $CDCl_3$), δ (ppm): 195.97; 147.43; 146.67; 135.93; 133.93; 128.86; 128.68; 127.98; 124.21; 78.80; 41.06; 38.97.

Synthesis of 4-nitro-3-(2-nitrophenyl)-1-phenylbutan-1-one (2g)

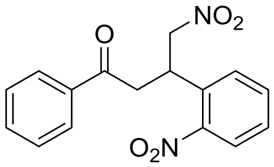

Following the general procedure described in the general conditions, 2.5 mmol of chalcone were dissolved in 2 mL of DMSO. After dissolution, 25 mg of powder silk fibroin were added and 25 mmol of CH_3NO_2 were injected. The reaction was let to proceed at 60°C for 1 hours. The crude product was purified through column chromatography (SiO_2, *n*-hexane/AcOEt 7:3). Isolated yield 72%.

Brown oil

^{1}H-NMR (500 MHz, $CDCl_3$), δ (ppm): 7.90 (d, *J*=7.2 Hz, 2H); 7.87 (d, *J*=8.1 Hz, 1H); 7.59- 7.53 (m, 2H); 7.49-7.38 (m, 4H); 4.94 (dd, *J*=13.1, 6.5 Hz, 1H); 4.89 (dd, *J*=13.1, 7.1 Hz, 1H); 4.75 (bquintuplet, *J*=6.8 Hz, 1H); 3.63 (dd, *J*=18.1, 6.6 Hz, 1H); 3.54 (dd, *J*=18.1, 7.2 Hz, 1H).

^{13}C-NMR (125 MHz, $CDCl_3$), δ (ppm): 196.35; 150.13; 136.11; 133.91; 133.89; 133.45; 128.91; 128.78; 128.61; 128.14; 125.27; 78.27; 40.97; 34.23.

Synthesis of 4-(1-nitro-4-oxo-4-phenylbutan-2-yl)benzonitrile (2h)

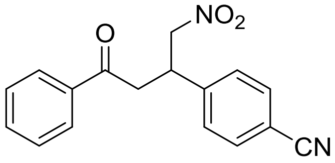

Following the general procedure described in the general conditions, 2.5 mmol of chalcone were dissolved in 2 mL of DMSO. After dissolution, 25 mg of powder silk fibroin were added and 25 mmol of CH_3NO_2 were injected. The reaction was let to proceed at 60°C for 1 hours. The ^{1}H-NMR spectroscopic analysis of crude product revealed a complete conversion of the substate, without the needing of chromatography purification. Isolated yield 90%.

White crystals. M.p.: 145-150°C (lit. 136 °C[8])

^{1}H-NMR (500 MHz, $CDCl_3$), δ (ppm): 7.93 – 7.87 (m, 2H); 7.66-7.62 (m, 2H); 7.62 – 7.56 (m, 1H); 7.49-7.41 (m, 4H); 4.85 (dd, *J*=12.9, 6.2 Hz, 1H); 4.71 (dd, *J*=12.9, 8.4 Hz, 1H); 4.34-4.26 (m, 1H); 3.53 – 3.41 (m, 2H).

^{13}C-NMR (125 MHz, $CDCl_3$), δ (ppm): 196.12; 144.69; 136.07; 134.02; 132.94; 128.97; 128.61; 128.09; 118.46; 111.99; 78.91; 41.11; 39.26.

Synthesis of 3-(benzo[d][1,3]dioxol-5-yl)-4-nitro-1-phenylbutan-1-one (2i)

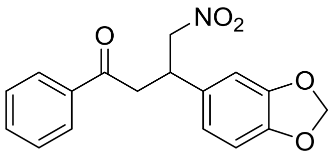

Following the general procedure described in the general conditions, 2.5 mmol of chalcone were dissolved in 2 mL of DMSO. After dissolution, 25 mg of powder silk fibroin were added and 25 mmol of CH_3NO_2 were injected. The reaction was let to proceed at 60°C for 1 hours. The crude product was purified through column chromatography (SiO_2, *n*-hexane/AcOEt 8:2). Isolated yield 53%.

Orange oil

^{1}H-NMR (500 MHz, $CDCl_3$), δ (ppm): 7.94-7.90 (m, 2H); 7.58 (t, *J*=7.5 Hz, 1H); 7.46 (t, *J*=7.7 Hz, 2H); 6.78-6.72 (m, 3H); 5.93 (s, 2H); 4.78 (dd, *J*=12.4, 6.7 Hz, 1H); 4.62 (dd, *J*=12.4, 8.1 Hz, 1H); 4.15 (m, 1H); 3.41 (dd, *J*=17.7, 6.5 Hz, 1H); 3.34 (dd, *J*=17.7, 3.4 Hz, 1H).

^{13}C-NMR (125 MHz, $CDCl_3$), δ (ppm): 196.88; 148.05; 147.10; 136.35; 133.56; 132.83; 128.73; 127.99; 120.79; 108.62; 107.76; 101.19; 79.77; 41.61; 39.10.

Synthesis of 1-(4-bromophenyl)-4-nitro-3-phenylbutan-1-one (2j)

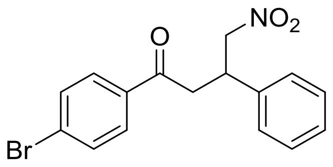

Following the general procedure described in the general conditions, 2.5 mmol of chalcone were dissolved in 2 mL of DMSO. After dissolution, 25 mg of powder silk fibroin were added and 25 mmol of CH_3NO_2 were injected. The reaction was let to proceed at 60°C for 1 hours. The ^{1}H-NMR spectroscopic analysis of crude product revealed a complete conversion of the substrate, without the needing of chromatography purification. Isolated yield 95%.

White crystals. M.p.: 90-96°C (lit. 87-89 °C[9])

^{1}H-NMR (500 MHz, $CDCl_3$), δ (ppm): 7.73 (d, *J*=8.6 Hz, 2H); 7.54 (d, *J*=8.6 Hz, 2H); 7.32- 7.27 (m, 2H); 7.27-7.22 (m, 3H); 4.78 (dd, *J*=12.6, 6.6 Hz, 1H); 4.65 (dd, *J*=12.6, 8.1 Hz, 1H); 4.21-4.14 (m, 1H); 3.41 (dd, *J*=17.8, 6.6 Hz, 1H); 3.36 (dd, *J*=17.8, 7.3 Hz, 1H).

^{13}C-NMR (125 MHz, $CDCl_3$), δ (ppm): 196.01; 139.07; 135.21; 132.17; 129.65; 129.23; 128.91; 128.08; 127.56; 79.62; 41.59; 39.37.

Synthesis of 4-nitro-1-(4-nitrophenyl)-3-phenylbutan-1-one (2k)

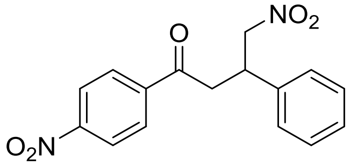

Following the general procedure described in the general conditions, 2.5 mmol of chalcone were dissolved in 2 mL of DMSO. After dissolution, 25 mg of powder silk fibroin were added and 25 mmol of CH_3NO_2 were injected. The reaction was let to proceed at 60°C for 1 hours. The ^{1}H-NMR spectroscopic analysis of crude product revealed a quantitative conversion, without the needing of chromatography purification. Isolated yield 94%.

Orange crystals. M.p.: 97-100°C (lit. 106-107 °C)[9]

^{1}H-NMR (500 MHz, $CDCl_3$), δ (ppm): 8.22 (d, *J*=8.5 Hz, 2H); 8.03 (d, *J*= 8.5 Hz, 2H); 7.30- 7.23 (m, 5H); 4.81 (dd, *J*=12.6, 6.9 Hz, 1H); 4.70 (dd, *J*=12.6, 7.8 Hz, 1H); 4.24-4.17 (m, 1H); 3.56-3.47 (m, 2H).

^{13}C-NMR (125 MHz, $CDCl_3$), δ (ppm): 195.67; 150.41; 140.75; 138.73; 129.12; 129.10; 128.02; 127.46; 123.88; 79.39; 42.08; 39.16.

Synthesis of 1-(naphthalen-2-yl)-4-nitro-3-phenylbutan-1-one (2l)

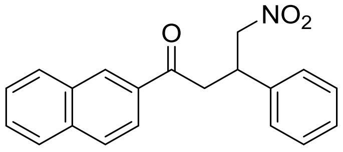

Following the general procedure described in the general conditions, 2.5 mmol of chalcone were dissolved in 2 mL of DMSO. After dissolution, 25 mg of powder silk fibroin were added and 25 mmol of CH_3NO_2 were injected. The reaction was let to proceed at 60°C for 1 hours. The ^{1}H-NMR spectroscopic analysis of crude product revealed a quantitative conversion, without the needing of chromatography purification. Isolated yield 98%.

White crystals. M.p 93-100°C.

^{1}H-NMR (500 MHz, $CDCl_3$), δ (ppm): 8.36 (s, 1H); 7.90 (dd, *J*=8.6, 1.7 Hz, 1H); 7.87 (d, *J*=8.2 Hz, 1H); 7.79 (d, *J*=8.8 Hz, 2H); 7.57-7.52 (m, 1H); 7.51-7.46 (m, 1H); 7.30-7.26 (m, 4H); 7.25-7.19 (m, 1H); 4.83 (dd, *J*=12.6, 6.4 Hz, 1H); 4.67 (dd, *J*=12.6, 8.3 Hz, 1H); 4.29-4.20 (m, 1H); 3.54 (dd, *J*=17.6, 6.4 Hz, 1H); 3.48 (dd, *J*=17.6, 7.5 Hz, 1H).

^{13}C-NMR (125 MHz, $CDCl_3$), δ (ppm): 196.90; 139.33; 135.85; 133.82; 132.53; 129.98; 129.71; 129.21; 128.89; 128.77; 128.01; 127.93; 127.63; 127.10; 123.68; 79.74; 41.73; 40.98 (DMSO); 39.56.

Synthesis of 3-(4-methoxyphenyl)-4-nitro-1-(4-nitrophenyl)butan-1-one (2m)

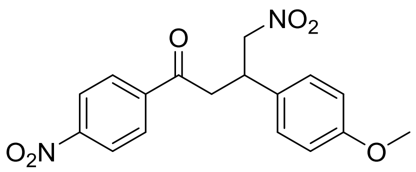

Following the general procedure described in the general conditions, 2.5 mmol of chalcone were dissolved in 2 mL of DMSO. After dissolution, 25 mg of powder silk fibroin were added and 25 mmol of CH_3NO_2 were injected. The reaction was let to proceed at 60°C for 1 hours. The ^{1}H-NMR spectroscopic analysis of crude product revealed a quantitative conversion, without the needing of chromatography purification. Isolated yield 91%.

Orange oil.

^{1}H-NMR (500 MHz, $CDCl_3$), δ (ppm): 8.24 (d, *J*=8.8 Hz, 2H); 8.02 (d, *J*=8.8 Hz, 2H); 7.17 (d, *J*=8.7 Hz, 2H); 6.81 (d, *J*=8.7 Hz, 2H); 4.77 (dd, *J*=12.5, 6.9 Hz, 1H); 4.65 (dd, *J*=12.5, 7.8 Hz, 1H); 4.15 (bquintuplet, *J*=7 Hz, 1H); 3.73 (s, 3H); 3.54 – 3.41 (m, 2H).

^{13}C-NMR (125 MHz, $CDCl_3$), δ (ppm): 195.76; 159.18; 150.43; 140.78; 130.48; 129.10; 128.53; 123.92; 114.48; 79.65; 55.24; 42.20; 38.50.

Synthesis of 1-(4-methoxyphenyl)-4-nitro-3-(4-nitrophenyl)butan-1-one (2n)

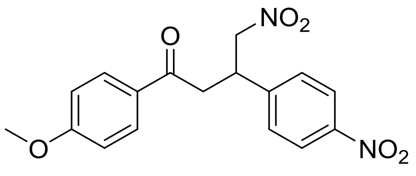

Following the general procedure described in the general conditions, 2.5 mmol of chalcone were dissolved in 2 mL of DMSO. After dissolution, 25 mg of powder silk fibroin were added and 25 mmol of CH_3NO_2 were injected. The reaction was let to proceed at 60°C for 1 hours. The crude product was purified through column chromatography (SiO_2, *n*-hexane/AcOEt 7:3). Isolated yield 65%.

Yellow oil

^{1}H-NMR (500 MHz, $CDCl_3$), δ (ppm): 8.14 (d, *J*=8.6 Hz, 2H); 7.87 (d, *J*=8.9 Hz, 2H); 7.48 (d, *J*=8.8 Hz, 2H); 6.89 (d, *J*=8.9 Hz, 2H); 4.72 (dd, *J*=13.0, 8.7 Hz, 1H); 4.33 (m, 1H); 3.83 (s, 3H); 3.48-3.37 (m, 2H).

^{13}C-NMR (125 MHz, $CDCl_3$), δ (ppm): 194.46; 164.13; 147.39; 146.97; 130.39; 129.06; 128.72; 124.20; 114.07; 78.91; 55.61; 40.71; 39.13.

Synthesis of 1,3-bis(4-methoxyphenyl)-4-nitrobutan-1-one (2o)

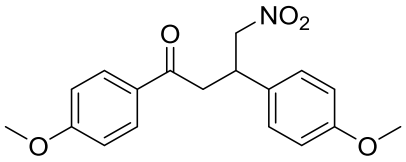

Following the general procedure described in the general conditions, 2.5 mmol of chalcone were dissolved in 2 mL of DMSO. After dissolution, 25 mg of powder silk fibroin were added and 25 mmol of CH_3NO_2 were injected. The reaction was let to proceed at 60°C for 1 hours. The crude product was purified through column chromatography (SiO_2, *n*-hexane/AcOEt 8:2). Isolated yield 65%.

Yellow oil

^{1}H-NMR (500 MHz, $CDCl_3$), δ (ppm): 7.88 (d, *J*=8.9 Hz, 2H); 7.18 (d, *J*=8.7 Hz, 2H); 6.90 (d, *J*=8.9 Hz, 2H); 6.84 (d, *J*=8.7 Hz, 2H); 4.79 (dd, *J*=12.4, 6.4 Hz, 1H); 4.62 (dd, *J*=12.4, 8.3 Hz, 12 1H); 4.20-4.11 (m, 1H); 3.84 (s, 3H); 3.75 (s, 3H); 3.37 (dd, *J*=17.3, 6.4 Hz, 1H); 3.31 (dd, *J*= 17.3, 7.6 Hz, 1H).

^{13}C-NMR (125 MHz, $CDCl_3$), δ (ppm): 195.61; 163.94; 159.16; 131.33; 130.48; 129.65; 128.64; 114.51; 114.00; 80.05; 55.65; 55.36; 41.43; 38.93.

Synthesis of 1,7-dinitro-2,6-diphenylheptan-4-one (5p)

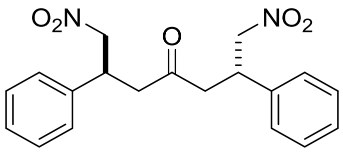

Following the general procedure described in the general conditions, 2.5 mmol of substate were dissolved in 2 mL of DMSO. After dissolution, 25 mg of powder silk fibroin were added and 25 mmol of CH_3NO_2 were injected. The reaction was let to proceed at 60°C for 1 hours. The crude product was purified through column chromatography (SiO_2, *n*-hexane/AcOEt 6:4). Isolated yield 40% 5p and 27% of 6p.

Orange oil.

^{1}H-NMR (500 MHz, $CDCl_3$), δ (ppm): 7.32-7.21 (m, 6H); 7.15-7.11 (m, 4H); 4.57-4.44 (m, 4H); 3.96-3.93 (m, 2H); 2.87-2.70 (m, 4H).

^{13}C-NMR (125 MHz, $CDCl_3$), δ (ppm): 204.86; 138.60; 138.57; 129.21; 129.19; 128.10; 128.05; 127.43; 127.37; 79.41; 79.33; 45.93; 45.88; 39.09; 39.01.

Synthesis of *anti*-4-nitro-3,5-diphenylcyclohexan-1-one (6p)

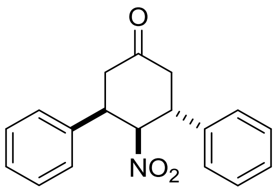

Following the general procedure described in the general conditions, 2.5 mmol of substate were dissolved in 2 mL of DMSO. After dissolution, 25 mg of powder silk fibroin were added and 2.5 mmol of CH_3NO_2 were injected. The reaction was let to proceed at 60°C for 1 hours. The crude product was purified through column chromatography (SiO_2, *n*-hexane/AcOEt 6:4). Isolated yield 60%.

Yellow oil.

^{1}H-NMR (500 MHz, $CDCl_3$), δ (ppm): 7.36-7.14 (m, 8H); 7.06-6.98 (m, 2H); 5.25 (dd, *J*= 7.0 Hz, 4.9 Hz, 1H); 3.94 (m, 1H); 3.75 (dt, *J* = 9.9, 5.2 Hz, 1H); 3.33-3.24 (m, 1H); 3.09-2.99 (m, 1H); 2.86-2.74.

^{13}C-NMR (125 MHz, $CDCl_3$), δ (ppm): 207.76; 139.48; 136.91; 129.44; 129.10; 128.49; 128.19; 127.68; 127.35; 91.59; 43.33; 42.59; 42.21; 41.93.

Synthesis of *anti*-1,7-dinitro-2,6-di-p-tolylheptan-4-one (5q)

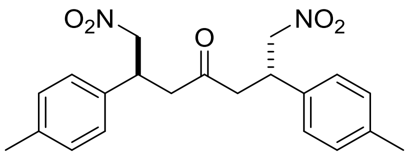

Following the general procedure described in the general conditions, 2.5 mmol of substate were dissolved in 7 mL of DMSO. After dissolution, 25 mg of powder silk fibroin were added and 25 mmol of CH_3NO_2 were injected. The reaction was let to proceed at 60°C for 1 hours. The crude product was purified through column chromatography (SiO_2, *n*-hexane/AcOEt 8:2). Isolated yield 40% of 5q and 57% of 6q.

White crystals. Mp: 160-165°C

^{1}H-NMR (500 MHz, $CDCl_3$), δ (ppm): 7.14-7.06 (m, 6H); 7.02 (t, *J*= 7.7 Hz, 2H); 4.57-4.43 (m, 4H); 3.94-3.81 (m, 2H); 2.90-2.68 (m, 4H); 2.32 (s, 3H); 2.31 (s, 3H, ethyl acetate partially overlap).

^{13}C-NMR (125 MHz, $CDCl_3$), δ (ppm): 205.15; 137.75; 137.73; 135.47; 135.44; 129.76; 129.73; 127.20; 127.15, 79.47; 79.41; 45.91; 45.88; 38.68; 38.61; 21.08; 21.05.

Synthesis of *anti*-4-nitro-3,5-di-p-tolylcyclohexan-1-one 133 (6q)

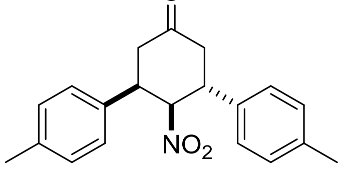

Following the general procedure described in the general conditions, 2.5 mmol of substate were dissolved in 7 mL of DMSO. After dissolution, 25 mg of powder silk fibroin were added and 2.5 mmol of CH_3NO_2 were injected. The reaction was let to proceed at 60°C for 1 hours. The crude product was purified through column chromatography (SiO_2, *n*-hexane/AcOEt 8:2). Isolated yield 74%.

Orange oil.

^{1}H-NMR (500 MHz, $CDCl_3$), δ (ppm): 7.18-7.08 (m, 6H); 6.95 (d, *J*= 8.1 Hz, 2H); 5.27 (dd, *J*= 7.2, 4.9 Hz); 3.88 (dd, *J*= 14.3, 7.3 Hz, 1H); 3.80-3.73 (m, 1H); 3.27 (dd, *J*= 15.9, 9.0 Hz, 1H); 3.02 (dd, *J*= 16.4, 6.0 Hz, 1H); 2.88-2.75 (m, 2H); 2.33 (s, 3H); 2.32 (s, 3H).

^{13}C-NMR (125 MHz, $CDCl_3$), δ (ppm): 208.03; 138.22; 137.90; 136.58; 134.03; 130.06; 129.76; 127.58; 127.24; 91.81; 43.58; 42.20; 42.15; 42.00; 21.19; 21.16.

Synthesis of (*E*)-6-nitro-1,5-di-o-tolylhex-1-en-3-one (4s)

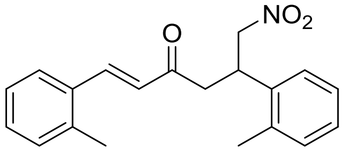

Following the general procedure described in the general conditions, 2.5 mmol of substate were dissolved in 2 mL of DMSO. After dissolution, 25 mg of powder silk fibroin were added and 25 mmol of CH_3NO_2 were injected. The reaction was let to proceed at 60°C for 1 hours. The crude product was purified through column chromatography (SiO_2, *n*-hexane/AcOEt 8:2). Isolated yield 80%.

Orange oil.

^{1}H-NMR (500 MHz, $CDCl_3$), δ (ppm): 7.84 (d, *J*= 16.0 Hz, 1H); 7.51 (d, *J*= 7.6 Hz, 1H); 7.30-7.16 (m, 7H, $CDCl_3$ signal); 6.60 (d, *J*= 16.0 Hz, 1H); 4.75 (dd, *J*= 12.5, 7.1 Hz, 1H); 4.65 (dd, *J*= 12.5, 7.7 Hz, 1H); 4.47-4.44 (m, 1H); 3.17-3-06 (m, 2H); 2.48 (s, 3H); 2.42 (s, 3H).

^{13}C-NMR (125 MHz, $CDCl_3$), δ (ppm): 196.90; 141.11; 138.39; 137.34; 136.59; 133.15; 131.35; 131.08; 130.68; 127.68; 126.75; 126.57; 126.50; 125.53; 79.15; 44.15; 34.63; 19.93; 19.74.

Synthesis of (*E*)-1,5-di(naphthalen-2-yl)-6-nitrohex-1-en-3-one (4t)

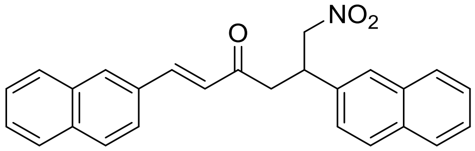

Following the general procedure described in the general conditions, 2.5 mmol of substate were dissolved in 2 mL of DMSO. After dissolution, 25 mg of powder silk fibroin were added and 25 mmol of CH_3NO_2 were injected. The reaction was let to proceed at 60°C for 1 hours. The crude product was purified through column chromatography (SiO_2, *n*-hexane/AcOEt 7:3). Isolated yield 49% of 4t and 22% of 6t.

Orange oil.

^{1}H-NMR (500 MHz, $CDCl_3$), δ (ppm): 7.85-7.78 (m, 6H); 7.71 (d, *J*= 16.1 Hz, 1H); 7.63 (dd, *J*= 8.7, 1.7 Hz, 1H); 7.56-7.43 (m, 6H); 7.45 (dd, *J*= 8.5, 1.9 Hz, 1H); 6.82 (d, *J*= 16.1 Hz, 1H); 4.91 (dd, *J*= 12.5, 6.6 Hz, 1H); 4.79 (dd, *J*= 12.5, 8.0 Hz, 1H); 4.37-4.32 (m, 1H); 3.33-3.23 (m, 2H).

^{13}C-NMR (125 MHz, $CDCl_3$), δ (ppm): 196.76; 143.95; 136.31; 134.69; 133.65; 133.47; 131.80; 130.99; 129.21; 129.06; 128.85; 128.06; 128.03; 127.90; 127.79; 127.08; 126.80; 126.70; 126.44; 125.89; 125.44; 125.32; 123.61; 79.72; 44.00; 39.76.

Synthesis of *anti*-3,5-di(naphthalen-2-yl)-4-nitrocyclohexan-1-one (6t)

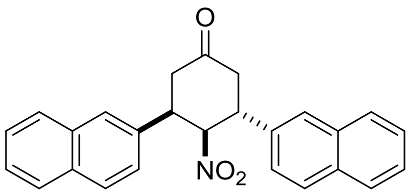

Following the general procedure described in the general conditions, 2.5 mmol of substate were dissolved in 2 mL of DMSO. After dissolution, 25 mg of powder silk fibroin were added and 2.5 mmol of CH_3NO_2 were injected. The reaction was let to proceed at 60°C for 1 hours. The crude product was purified through column chromatography (SiO_2, *n*-hexane/AcOEt 7:3). Isolated yield 61%.

Orange oil.

^{1}H-NMR (500 MHz, $CDCl_3$), δ (ppm): 7.89-7.76 (m, 6H); 7.72 (brs, 1H); 7.55-7.48 (m, 5H); 7.39 (dd, *J*= 8.5, 1.9 Hz, 1H); 7.14 (dd, *J*= 8.5, 1.9 Hz, 1H); 5.50 (dd, *J*= 6.5, 4.8 Hz, 1H); 4.19-4.08 (m, 1H, ethyl acetate traces); 3.96 (dt, *J*= 9.9, 5.1 Hz, 1H); 3.50 (dd, *J*= 15.7, 9.4 Hz, 1H); 3.20 (dd, *J*= 16.4, 6.1 Hz, 1H); 3.04-2.95 (m, 2H).

^{13}C-NMR (125 MHz, $CDCl_3$), δ (ppm): 207.87; 136.79; 134.34; 133.51; 133.34; 133.05; 132.87; 129.51; 128.93; 128.15; 128.11; 127.82; 127.77; 126.94; 126.79; 126.74; 126.72; 126.66; 126.58; 125.37; 125.03; 91.45; 43.11; 43.00; 42.14; 41.98.

Synthesis of *anti*-4-nitro-3,5-di(pyridin-2-yl)cyclohexan-1-one (6u)

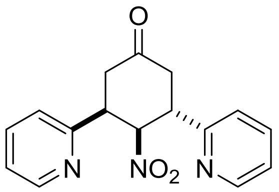

Following the general procedure described in the general conditions, 2.5 mmol of substate were dissolved in 2 mL of DMSO. After dissolution, 25 mg of powder silk fibroin were added and 25 mmol of CH_3NO_2 were injected. The reaction was let to proceed at 60°C for 1 hours. The crude product was purified through column chromatography (SiO_2, *n*-hexane/AcOEt 7:3). Isolated yield 25%.

Orange oil.

^{1}H-NMR (500 MHz, $CDCl_3$), δ (ppm): 8.61 (m, 1H); 8.51 (m, 1H); 7.82-7.44 (m, 2H); 7.23 (dd, *J*= 7.2, 5.3 Hz, 1H); 7.18-7.09 (m, 2H); 7.02 (d, *J*= 7.7 Hz, 1H); 5.95 (dd, *J*= 10.6, 4.7, 1H); 4.26-4.12 (m, 2H); 3.00-2.84 (m, 3H); 2.75 (dd, *J*= 16.6, 11.4 Hz, 1H).

^{13}C-NMR (125 MHz, $CDCl_3$), δ (ppm): 205.06; 159.03; 157.25; 149.78; 149.50; 137.12; 137.05; 123.80; 123.21; 123.11; 122.65; 88.59; 45.88; 45.99; 43.34; 42.28.

NMR Spectra

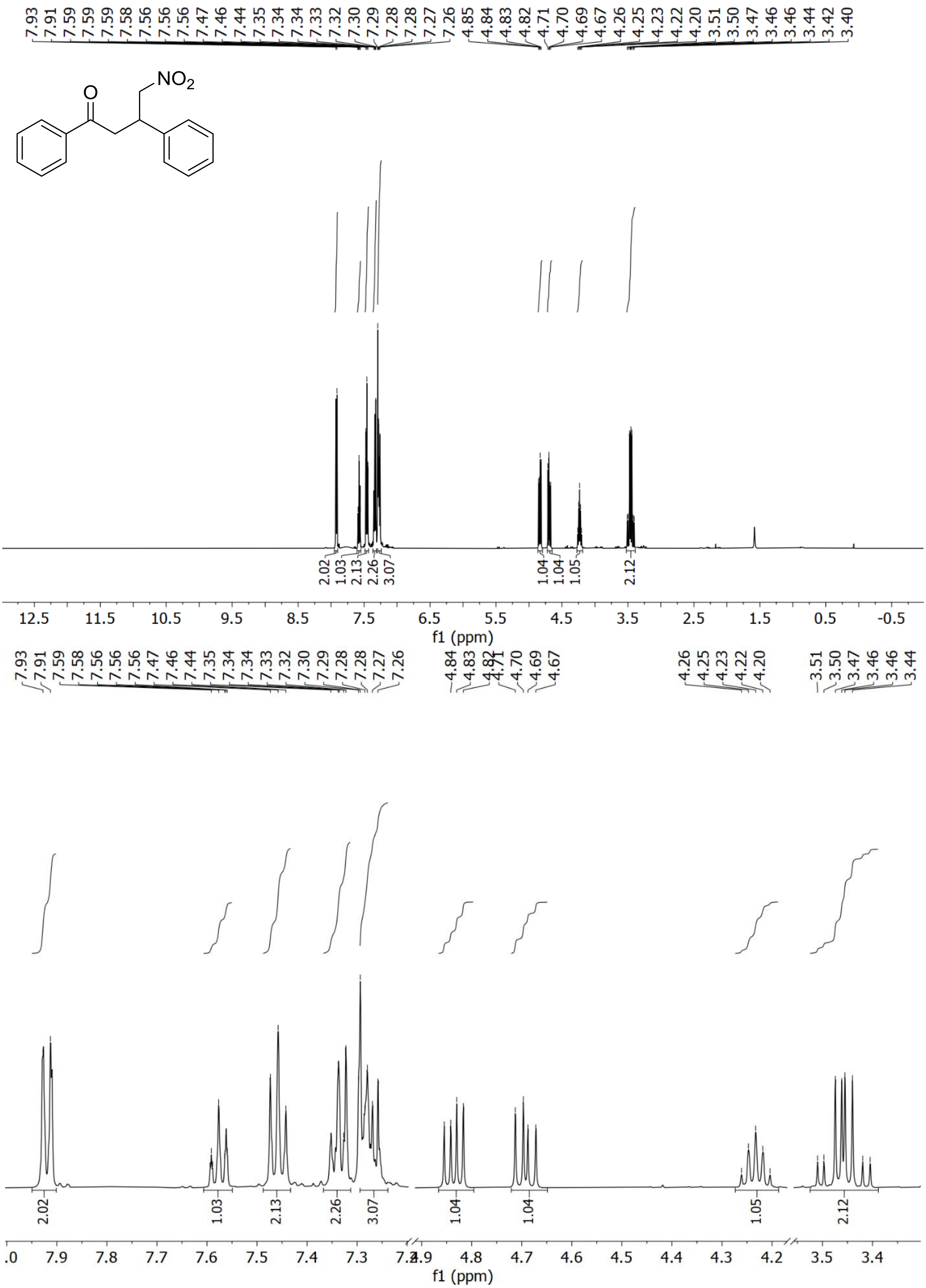

Figure S6: *^{1}H-NMR spectrum (500 MHz, $CDCl_3$) for the pure product of 4-nitro-1,3-diphenylbutan-1-one (2a): full scale spectrum (top) and spectrum expansions (bottom).*

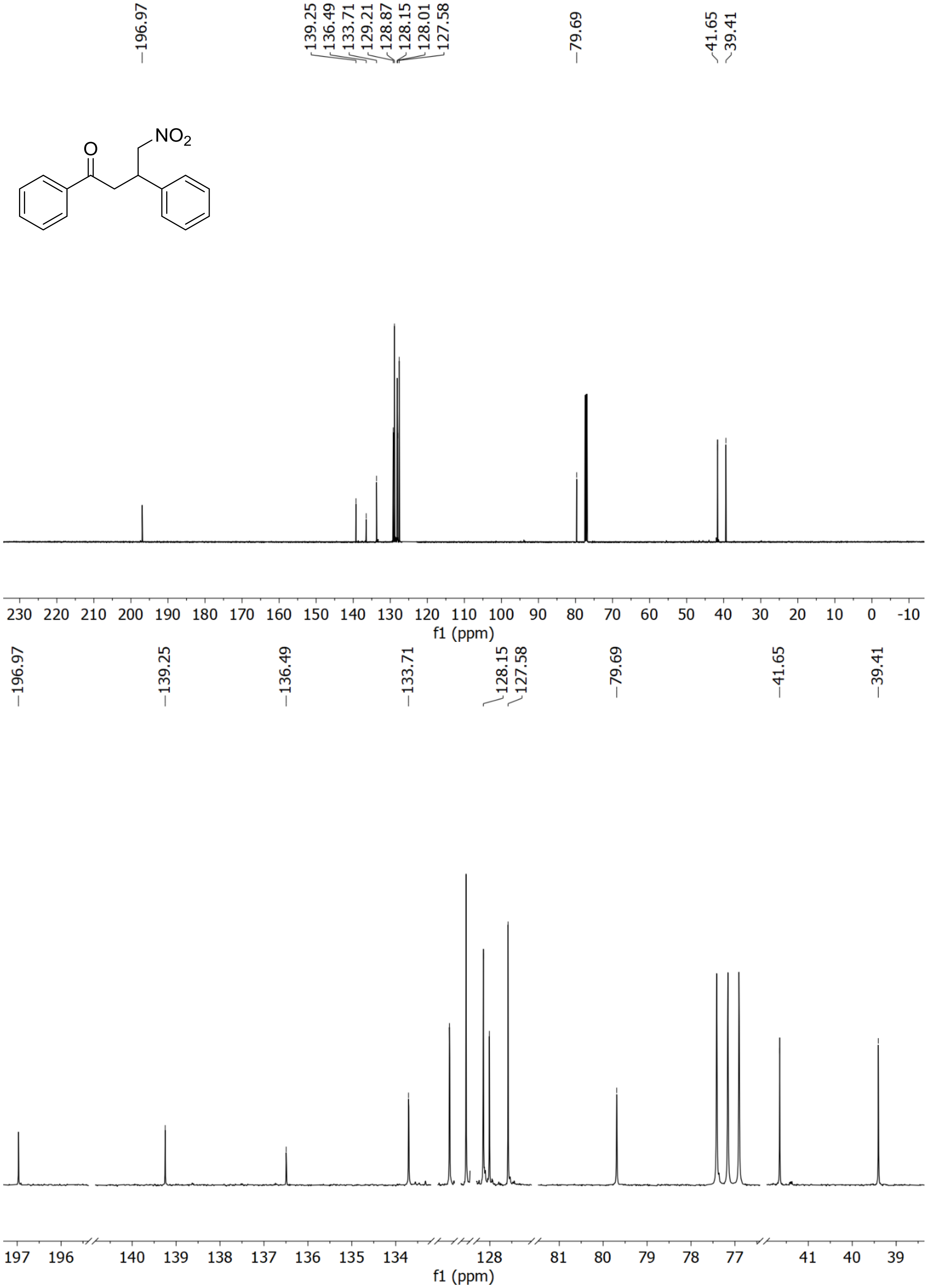

Figure S7: *^{13}C-NMR spectrum (125 MHz, $CDCl_3$) for the pure product of 4-nitro-1,3-diphenylbutan-1-one (2a): full scale spectrum (top) and spectrum expansions (bottom).*

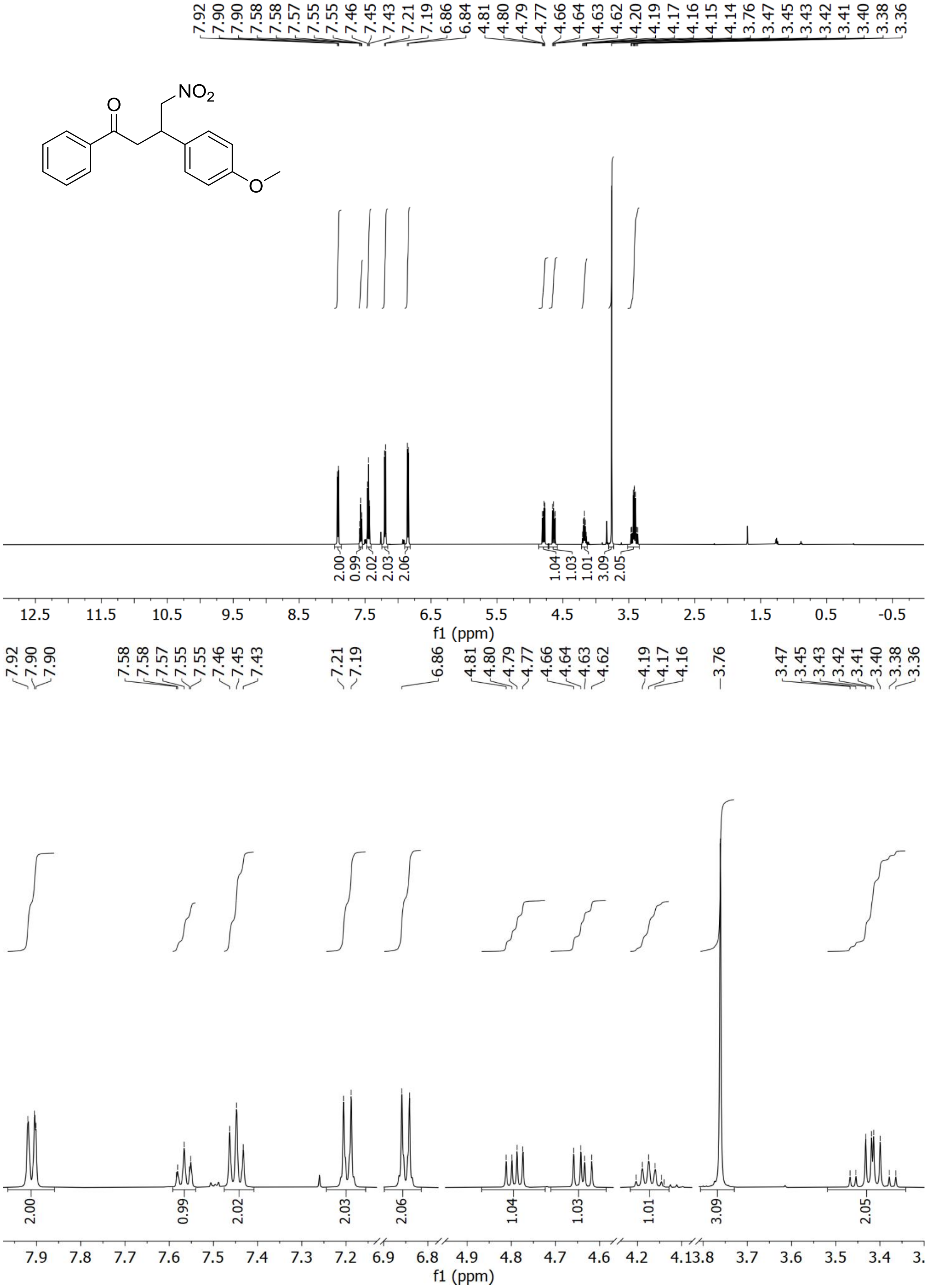

Figure S8: *^{1}H-NMR spectrum (500 MHz, $CDCl_3$) for the pure product of 3-(4-methoxyphenyl)-4-nitro-1-phenylbutan-1-one (2b): full scale spectrum (top) and spectrum expansions (bottom).*

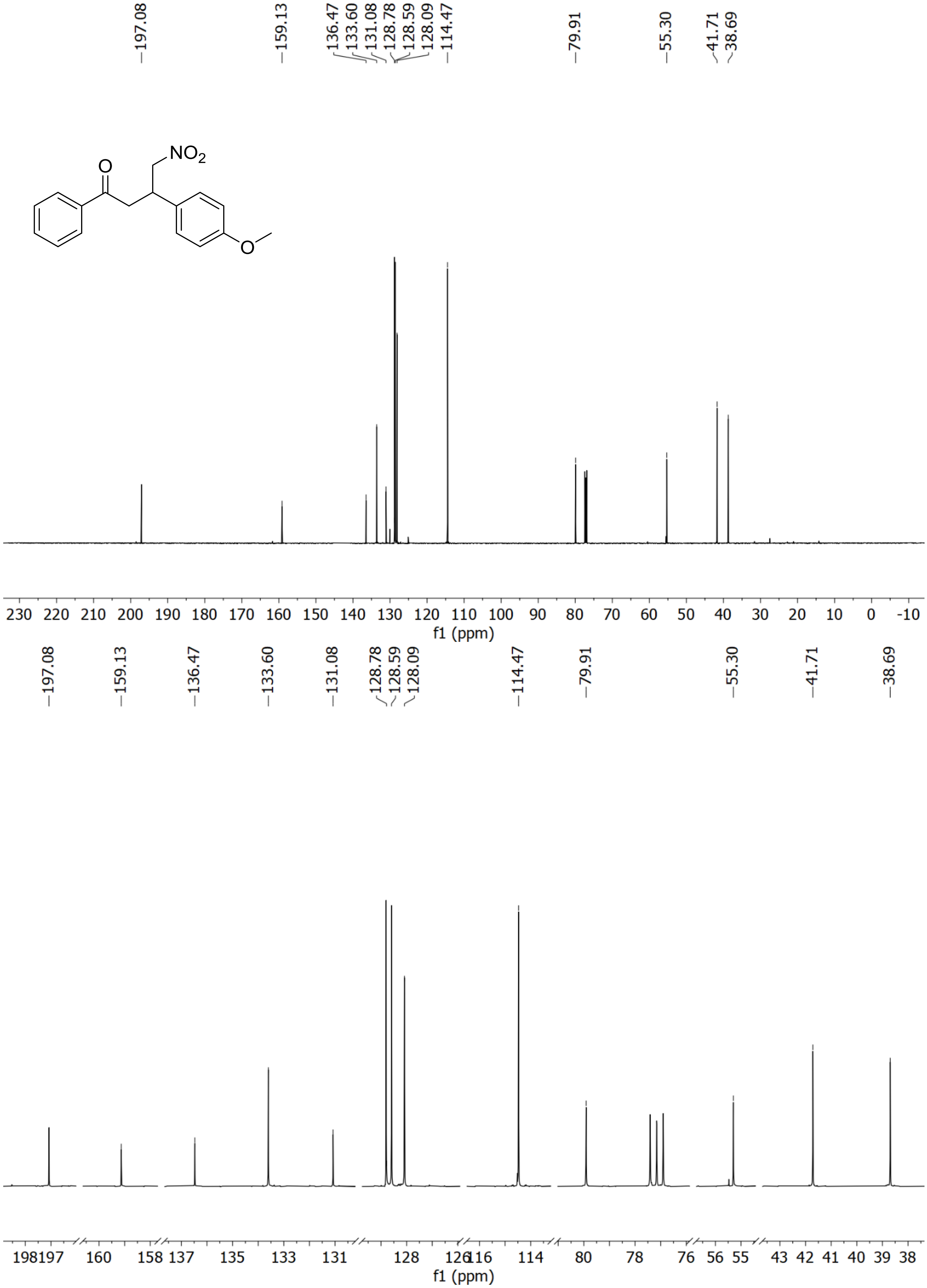

Figure S9: *^{13}C NMR spectrum (125 MHz, $CDCl_3$) for the pure product of 3-(4-methoxyphenyl)-4-nitro-1-phenylbutan-1-one (2b): full scale spectrum (top) and spectrum expansions (bottom).*

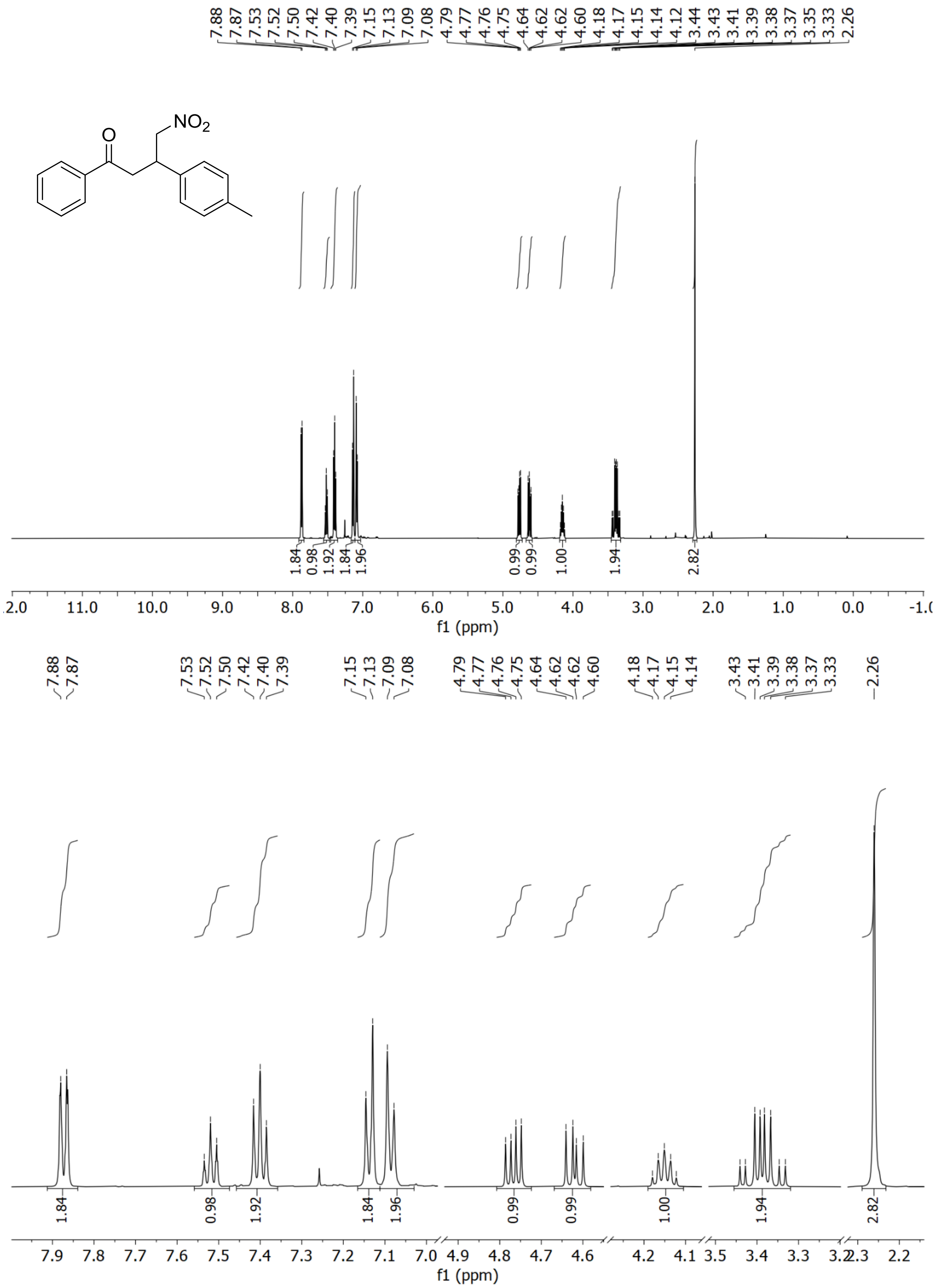

Figure S10: *^{1}H-NMR spectrum (500 MHz, $CDCl_3$) for the pure product of 4-nitro-1-phenyl-3-(p-tolyl)butan-1-one (2c): full scale spectrum (top) and spectrum expansions (bottom).*

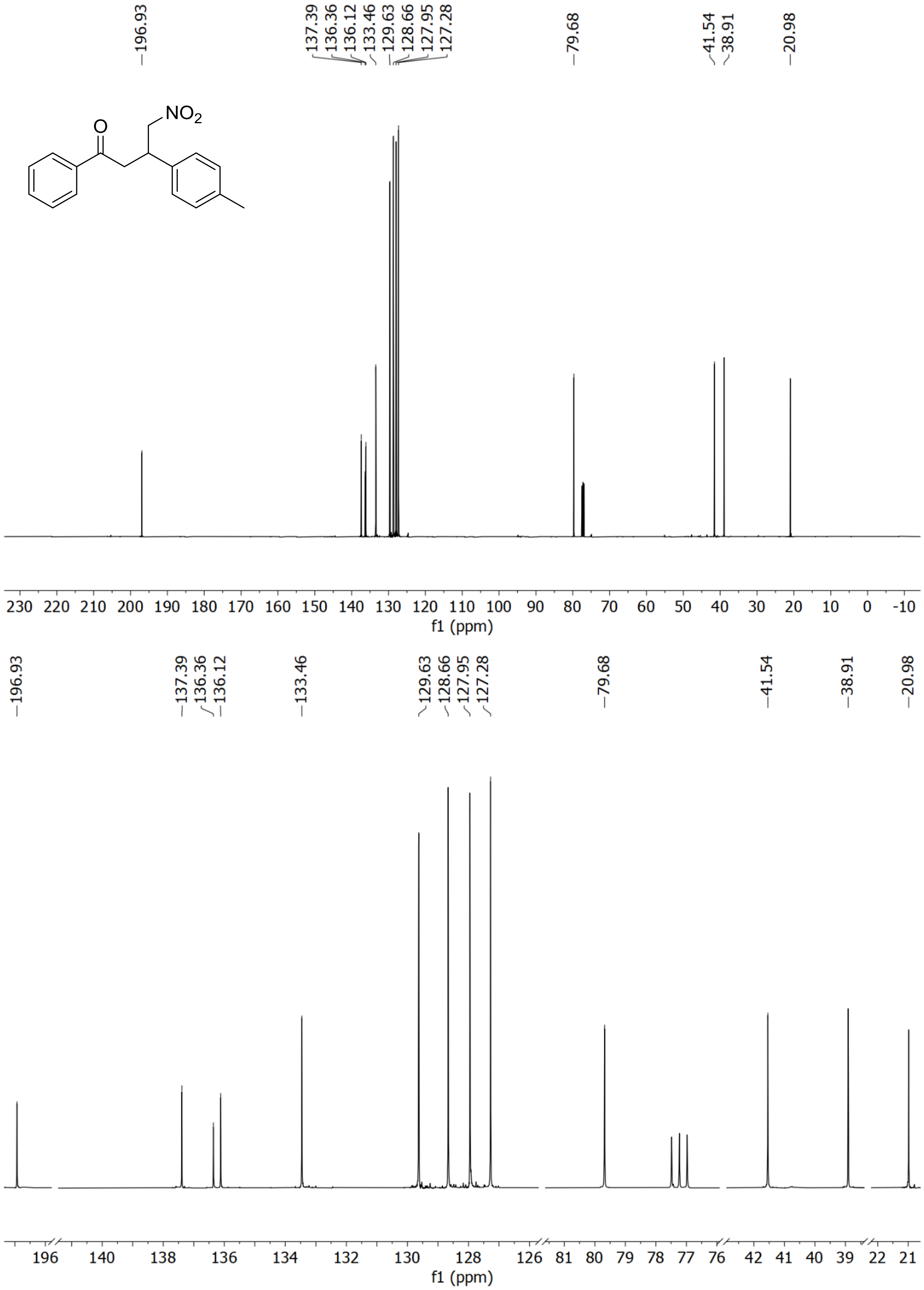

Figure S11: *^{13}C NMR spectrum (125 MHz, $CDCl_3$) for the pure product of 4-nitro-1-phenyl-3-(p-tolyl)butan-1-one (2c): full scale spectrum (top) and spectrum expansions (bottom).*

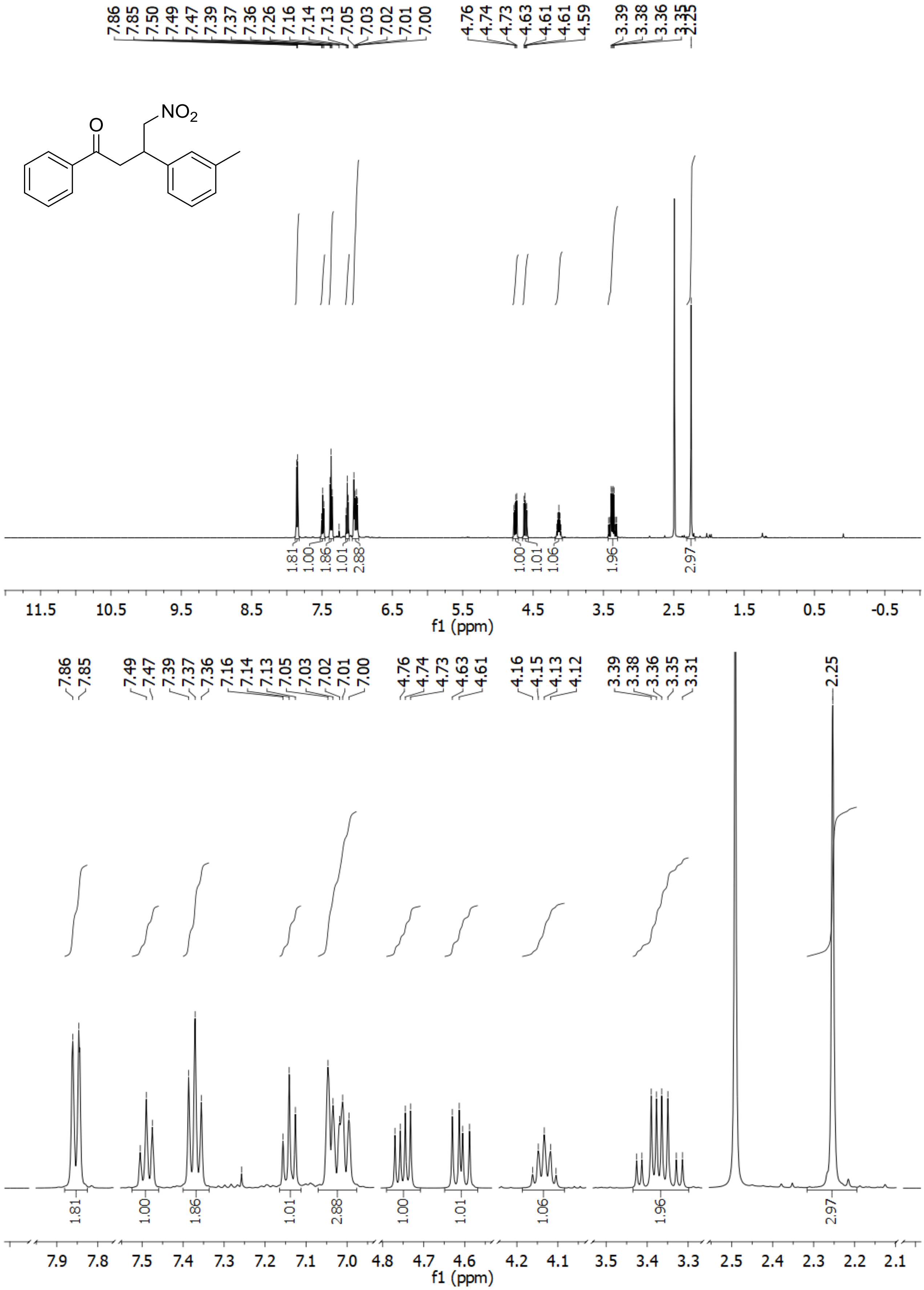

Figure S12: *^{1}H-NMR spectrum (500 MHz, $CDCl_3$) for the pure product of 4-nitro-1-phenyl-3-(m-tolyl)butan-1-one (2d): full scale spectrum (top) and spectrum expansions (bottom).*

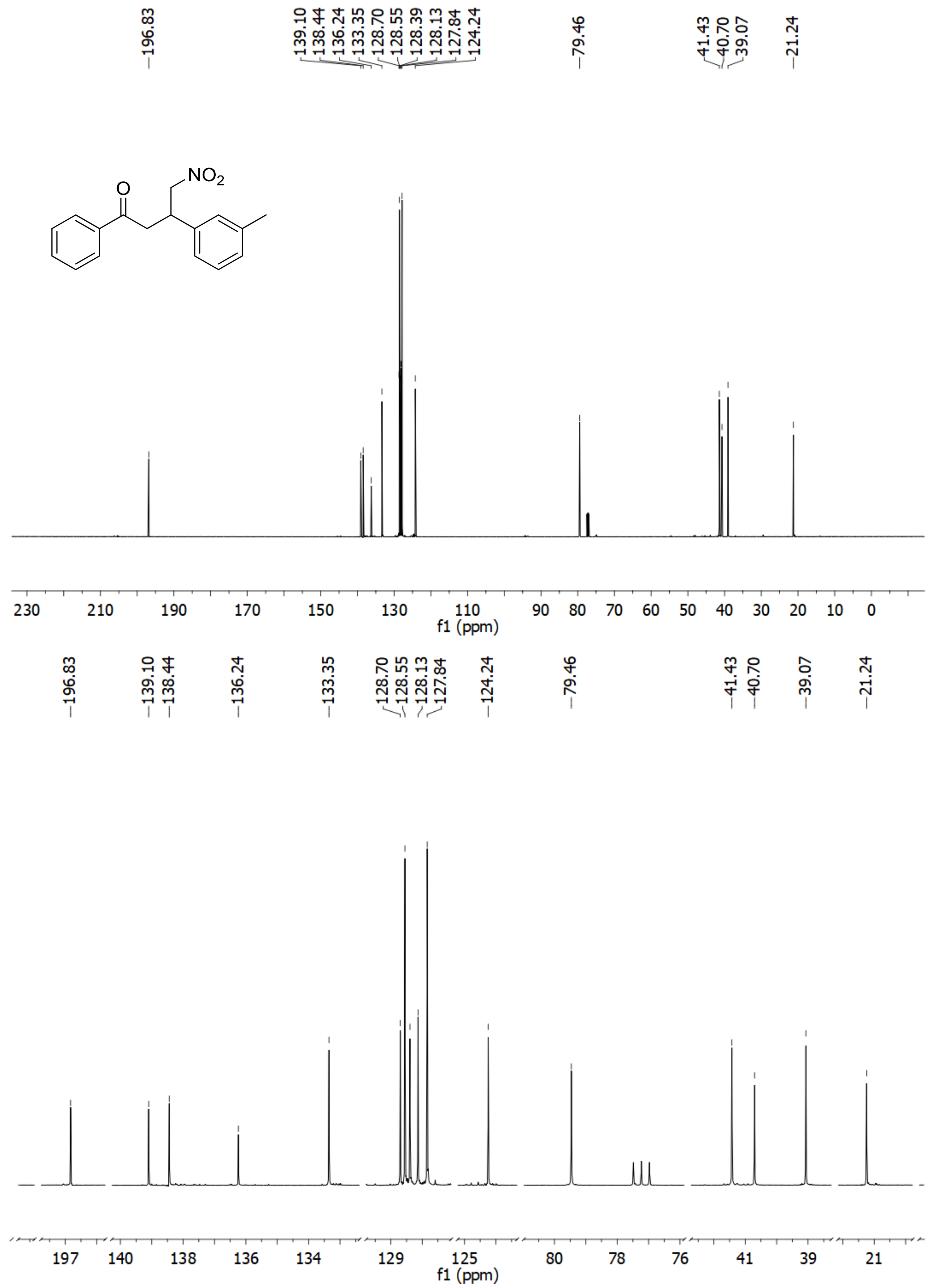

Figure S13: *^{13}C NMR spectrum (125 MHz, $CDCl_3$) for the pure product of 4-nitro-1-phenyl-3-(m-tolyl)butan-1-one (2d): full scale spectrum (top) and spectrum expansions (bottom).*

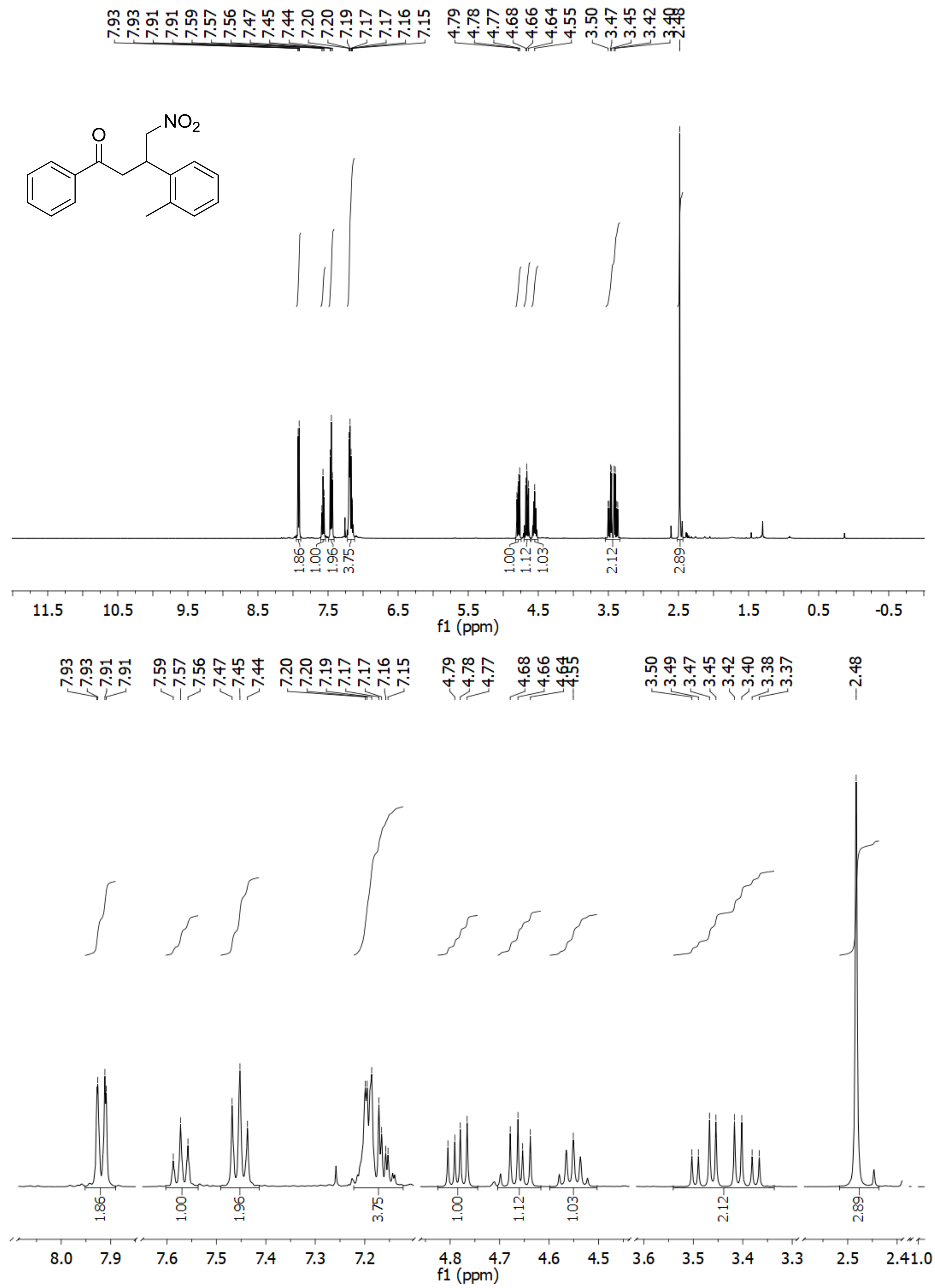

Figure S14: *^{1}H-NMR spectrum (500 MHz, $CDCl_3$) for the pure product of 4-nitro-1-phenyl-3-(o-tolyl)butan-1-one (2e): full scale spectrum (top) and spectrum expansions (bottom).*

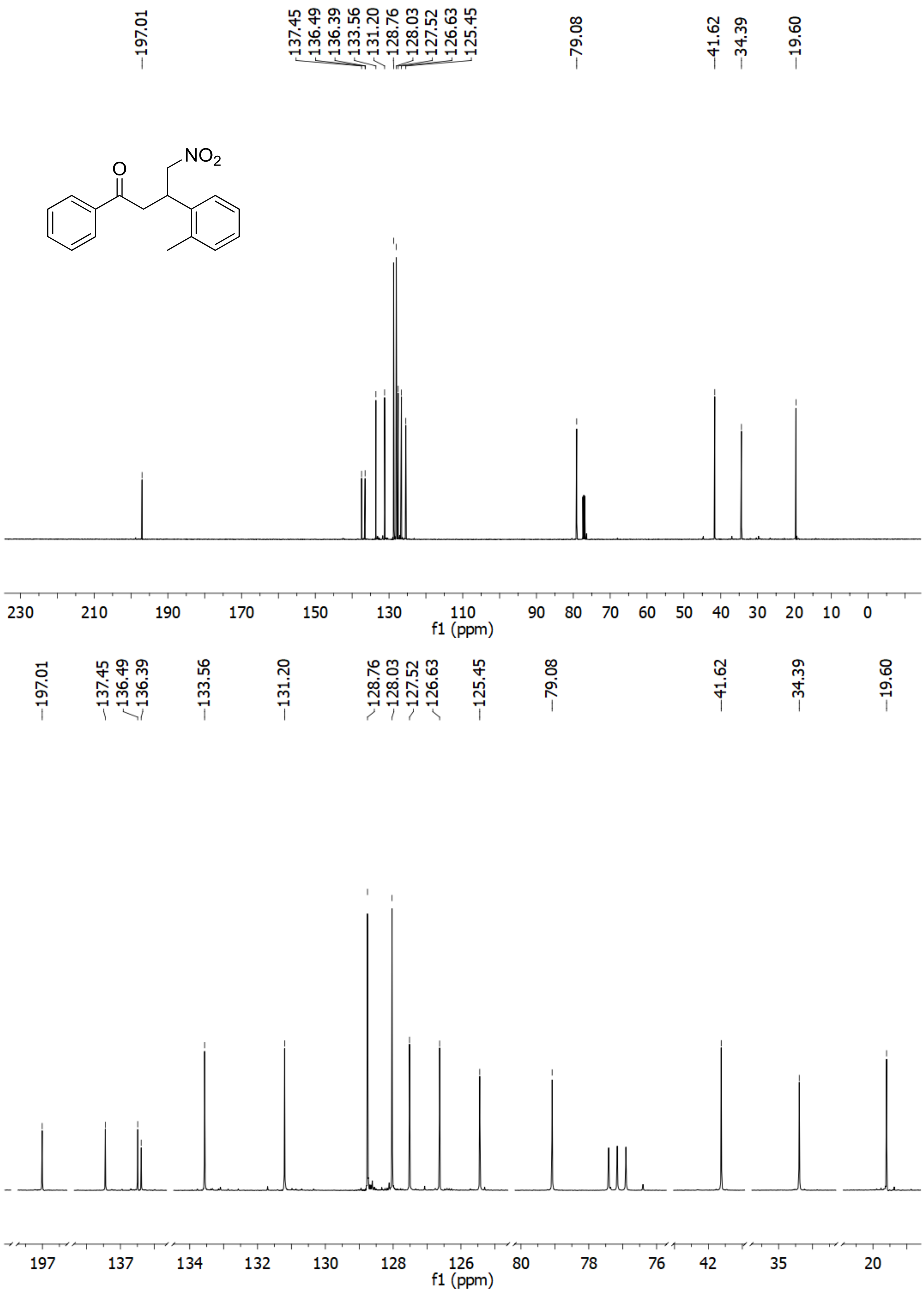

Figure S15: *^{13}C NMR spectrum (125 MHz, $CDCl_3$) for the pure product of 4-nitro-1-phenyl-3-(m-tolyl)butan-1-one (2e): full scale spectrum (top) and spectrum expansions (bottom).*

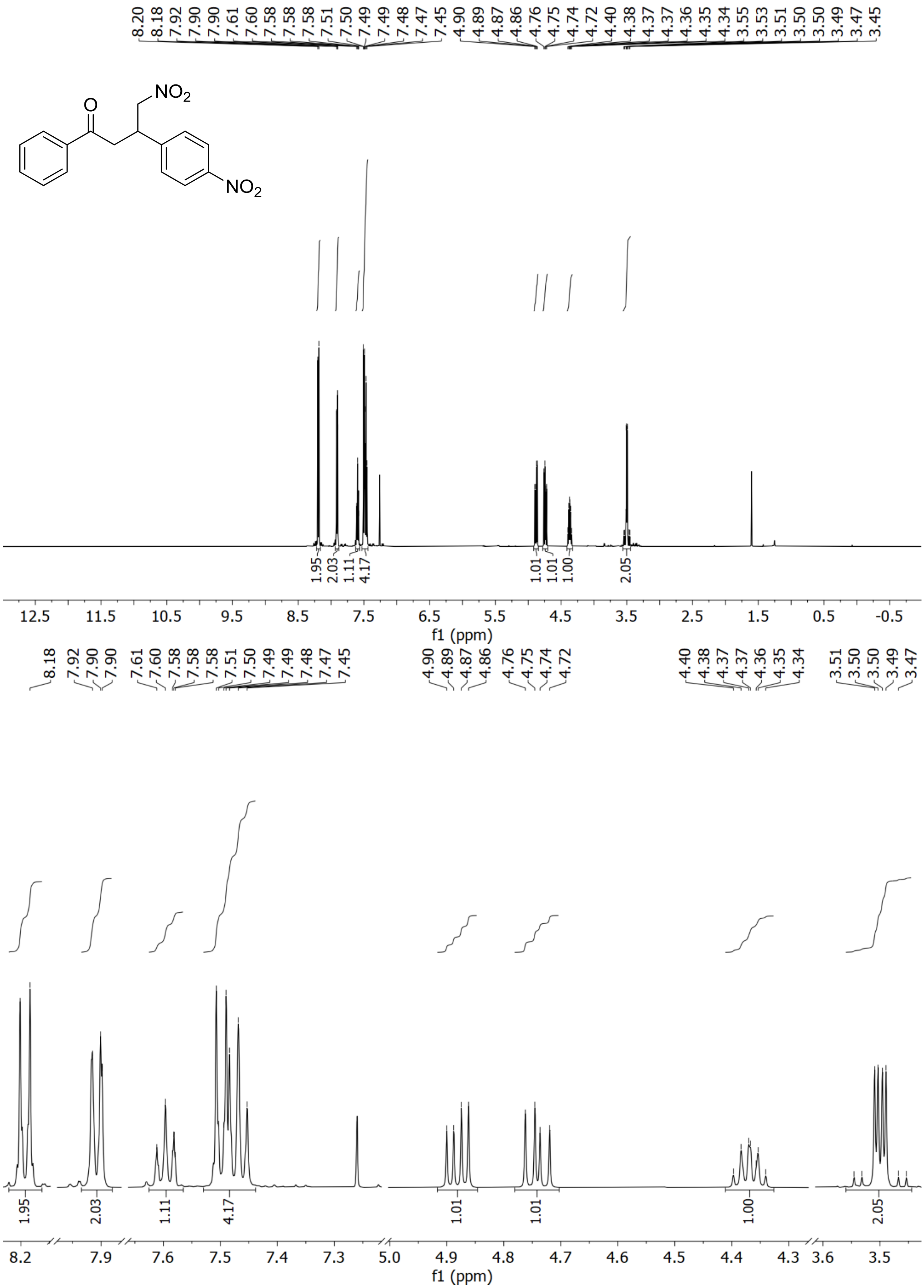

Figure S16: *^{1}H-NMR spectrum (500 MHz, $CDCl_3$) for the pure product of 4-nitro-3-(4-nitrophenyl)-1-phenylbutan-1-one (2f): full scale spectrum (top) and spectrum expansions (bottom).*

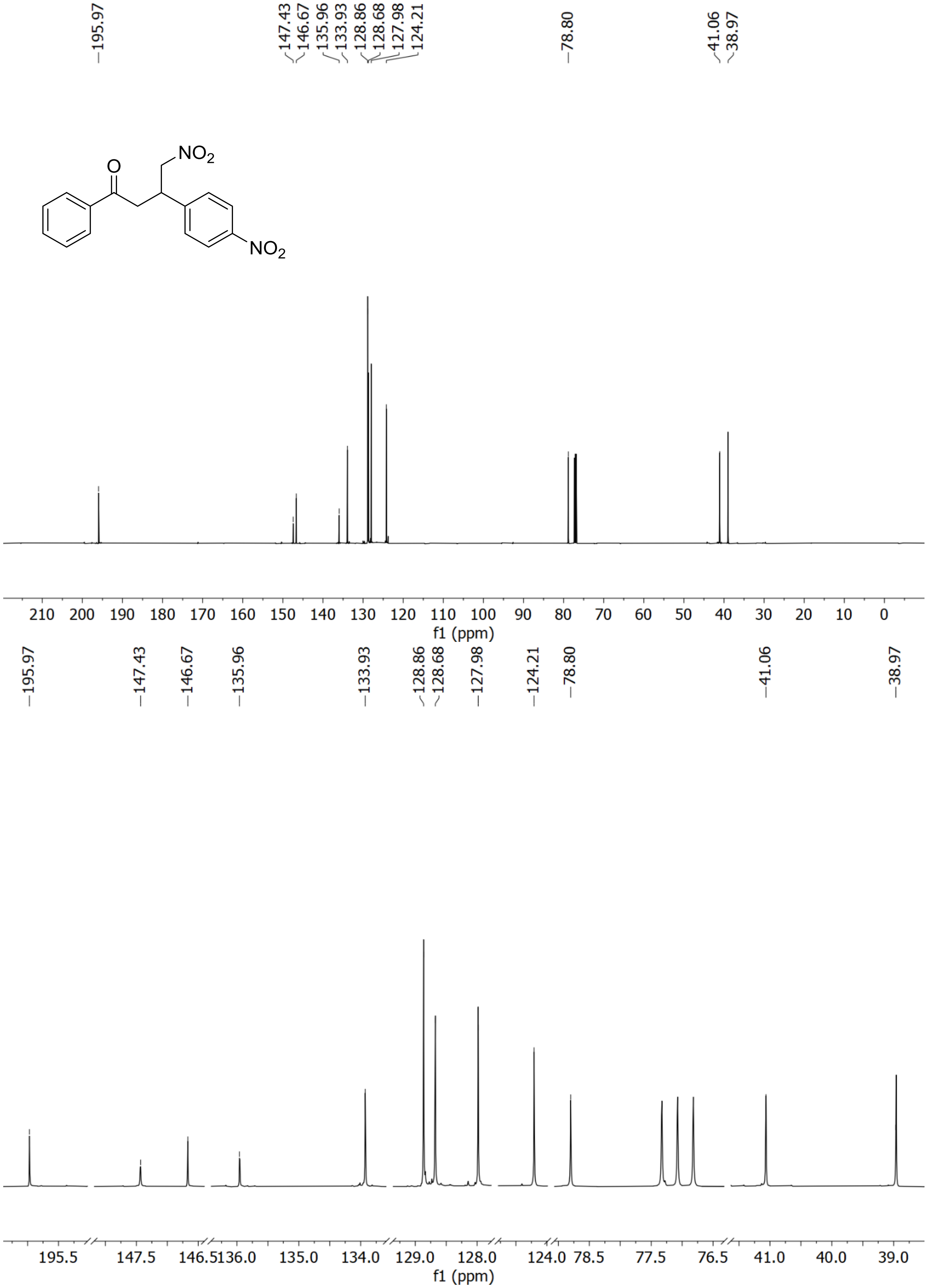

Figure S17: *^{13}C NMR spectrum (125 MHz, $CDCl_3$) for the pure product of 4-nitro-3-(4-nitrophenyl)-1-phenylbutan-1-one (2f): full scale spectrum (top) and spectrum expansions (bottom).*

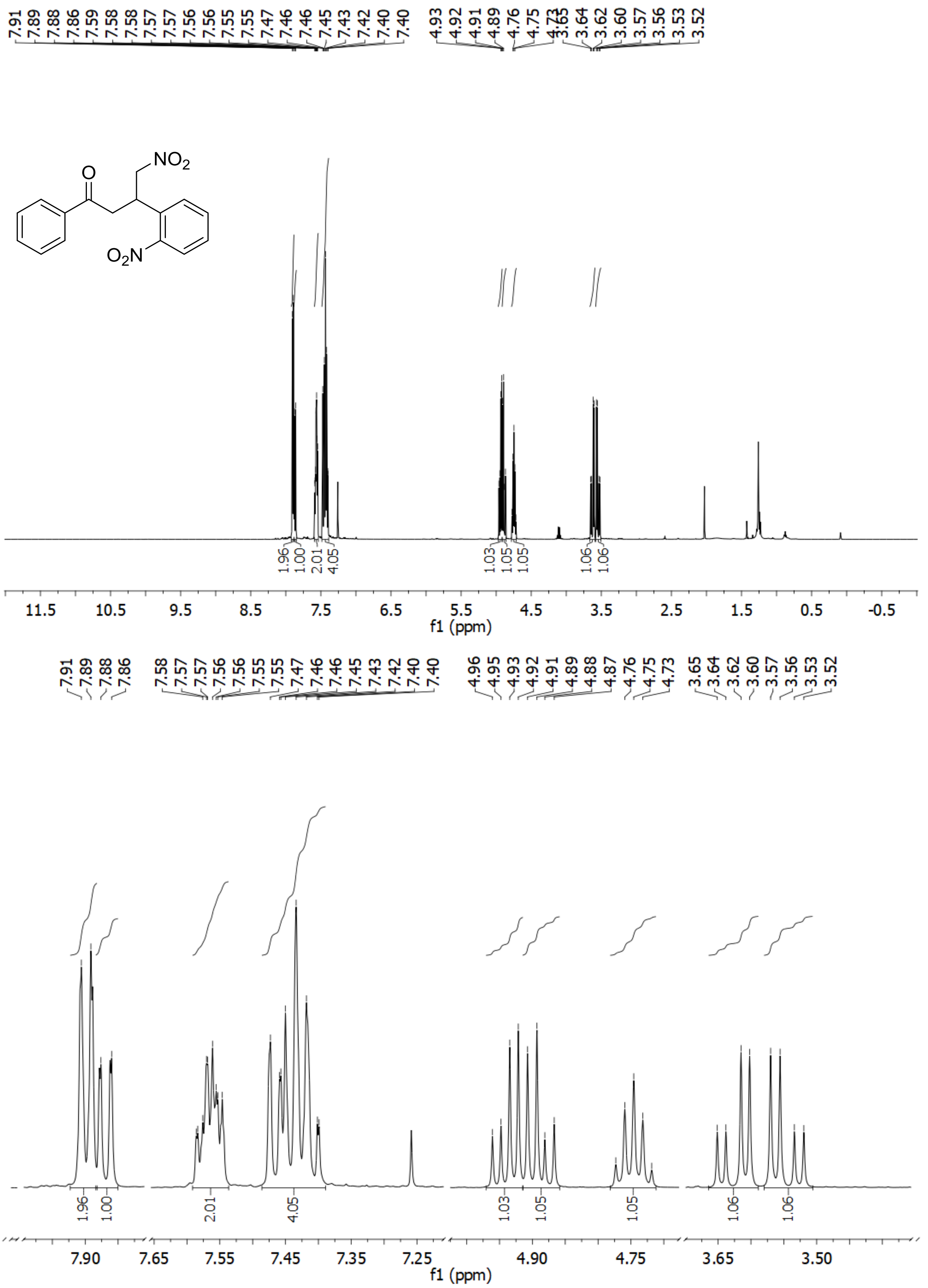

Figure S18: *^{1}H-NMR spectrum (500 MHz, $CDCl_3$) for the pure product of 4-nitro-3-(2-nitrophenyl)-1-phenylbutan-1-one (2g): full scale spectrum (top) and spectrum expansions (bottom).*

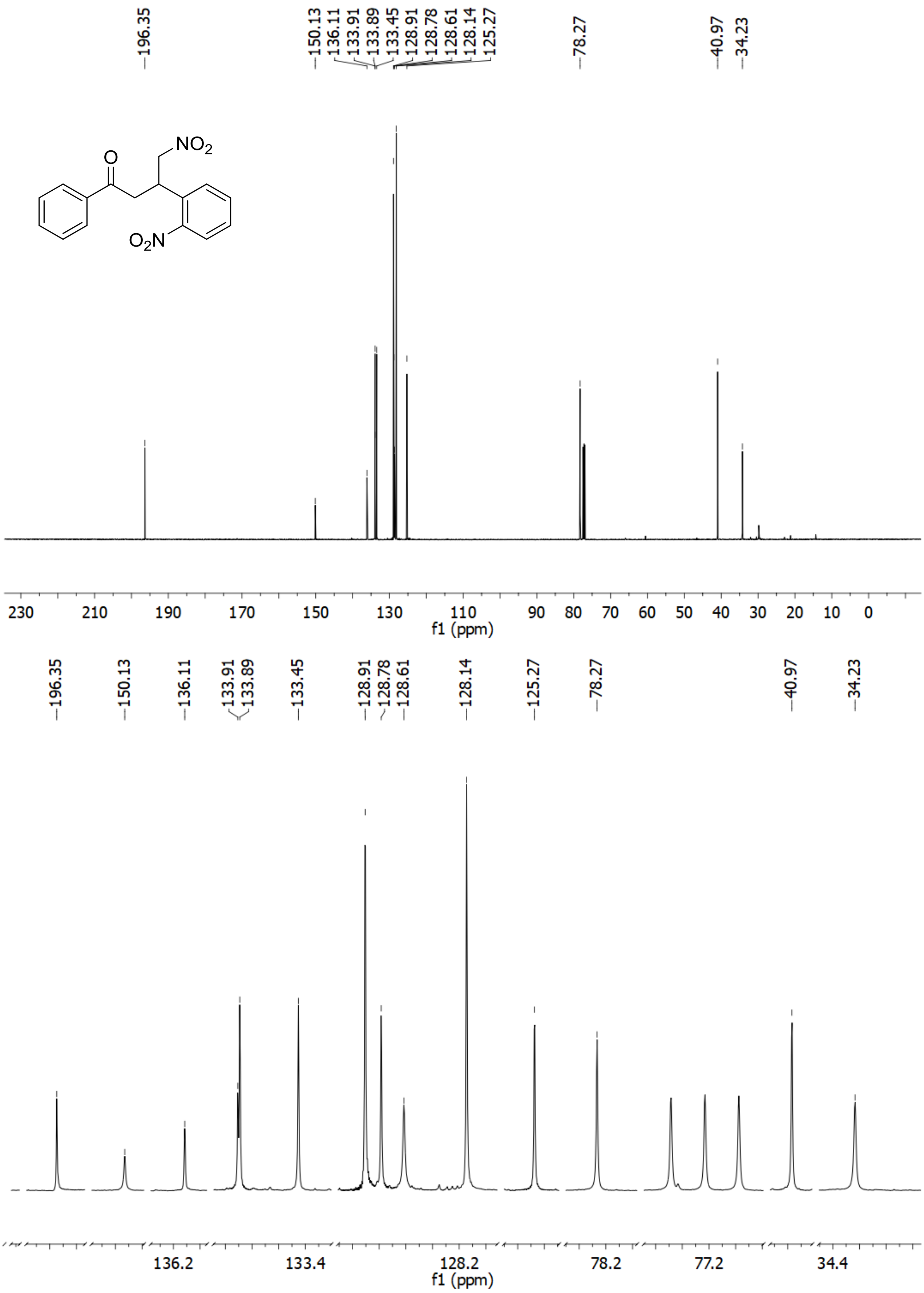

Figure S19: *^{13}C NMR spectrum (125 MHz, $CDCl_3$) for the pure product of 4-nitro-3-(2-nitrophenyl)-1-phenylbutan-1-one (2g): full scale spectrum (top) and spectrum expansions (bottom).*

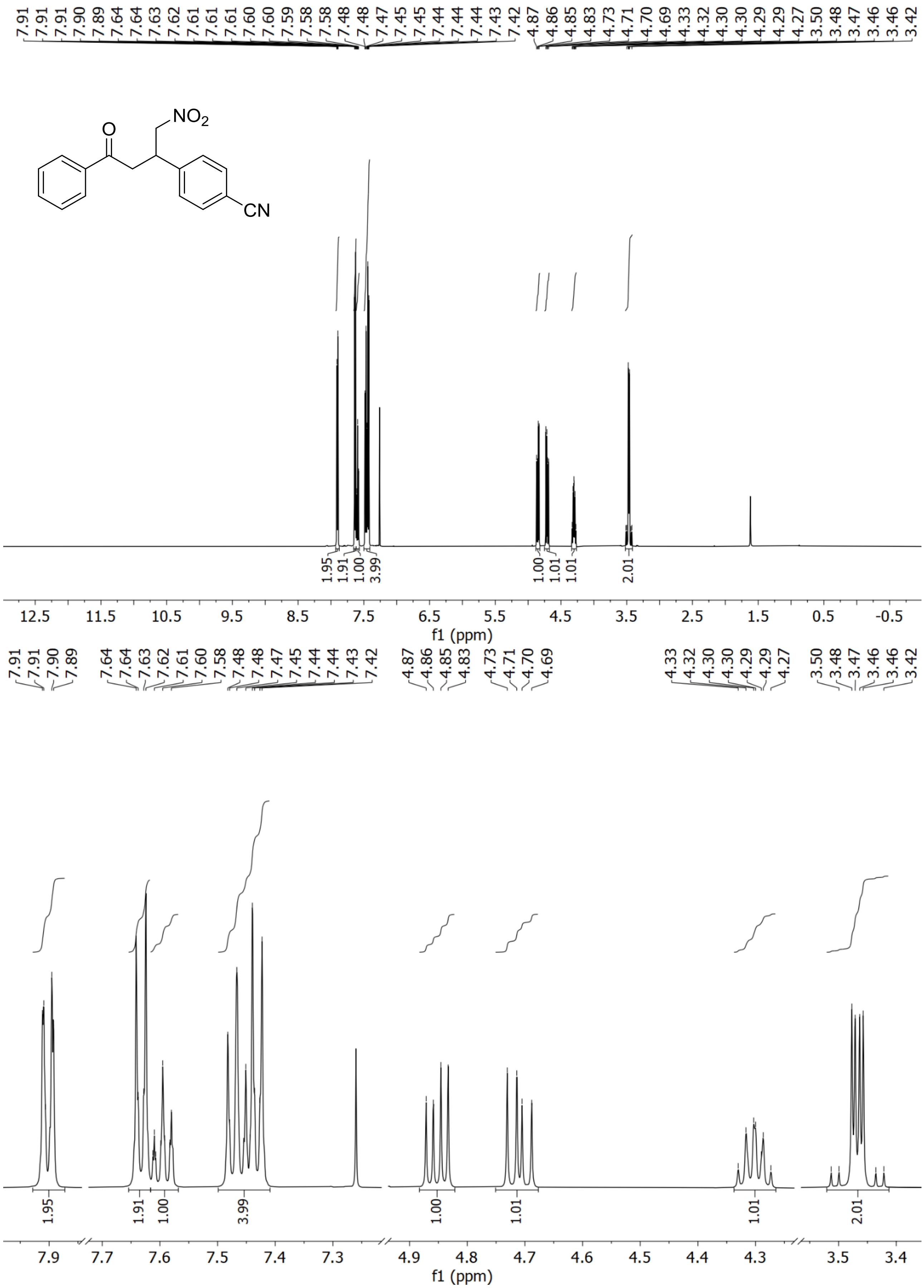

Figure S20: *^{1}H-NMR spectrum (500 MHz, $CDCl_3$) for the pure product of 4-(1-nitro-4-oxo-4-phenylbutan-2-yl)benzonitrile (2h): full scale spectrum (top) and spectrum expansions (bottom).*

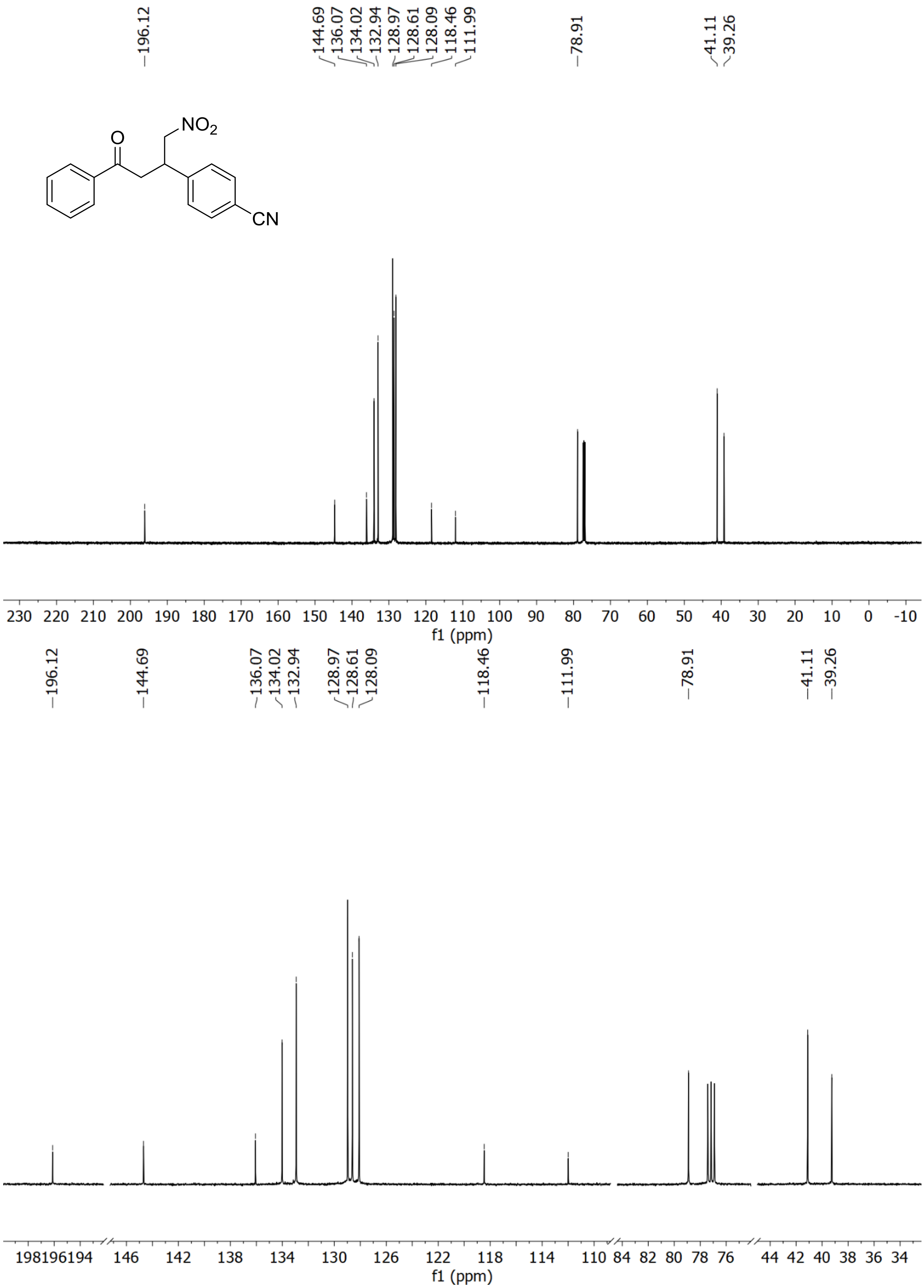

Figure S21: ^{13}C NMR spectrum (125 MHz, $CDCl_3$) for the pure product of 4-(1-nitro-4-oxo-4-phenylbutan-2-yl)benzonitrile (2h): full scale spectrum (top) and spectrum expansions (bottom).

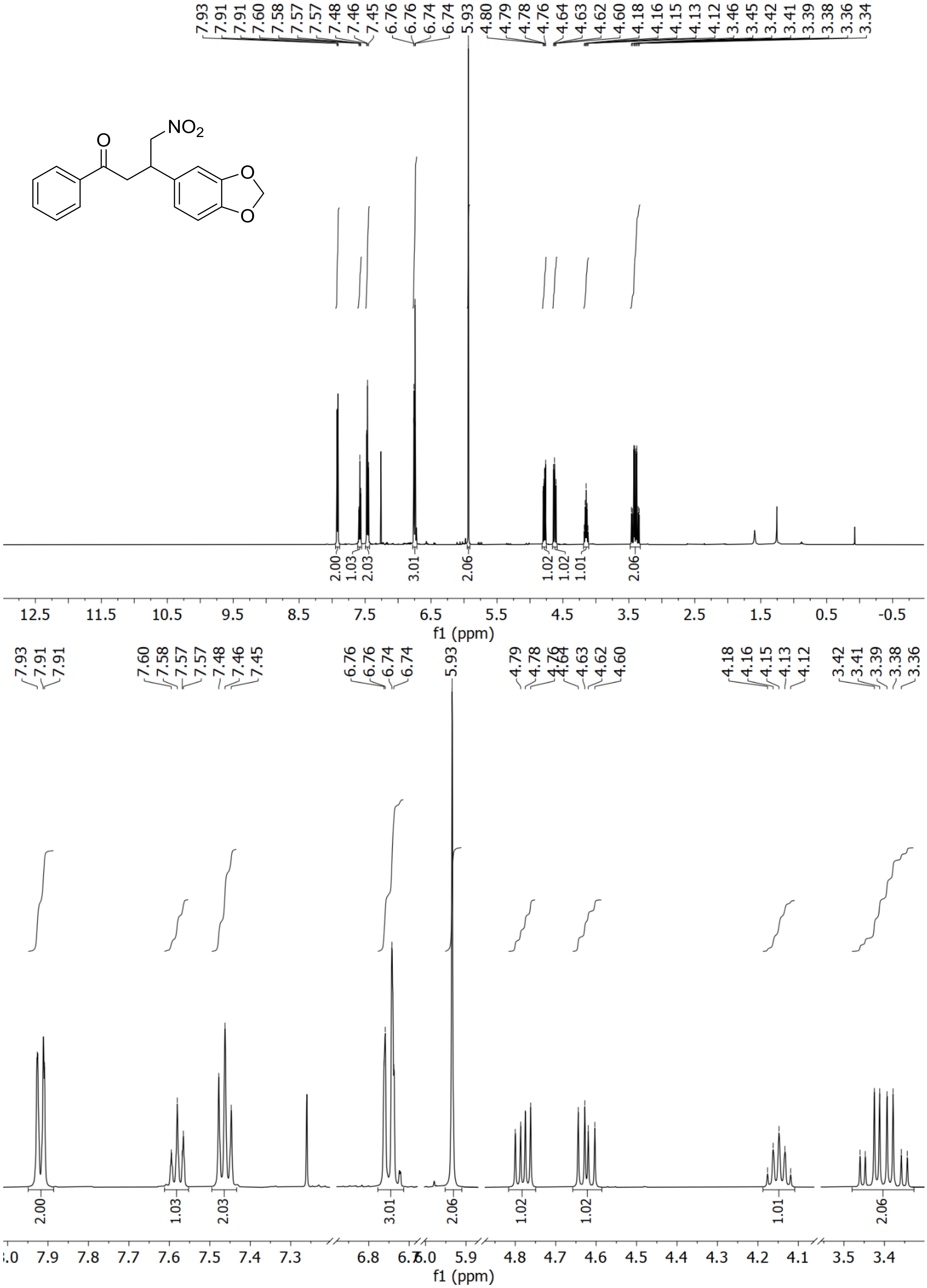

Figure S22: *^{1}H-NMR spectrum (500 MHz, $CDCl_3$) for the pure product of 3-(benzo[d][1,3]dioxol-5-yl)-4-nitro-1-phenylbutan-1-one (2i): full scale spectrum (top) and spectrum expansions (bottom).*

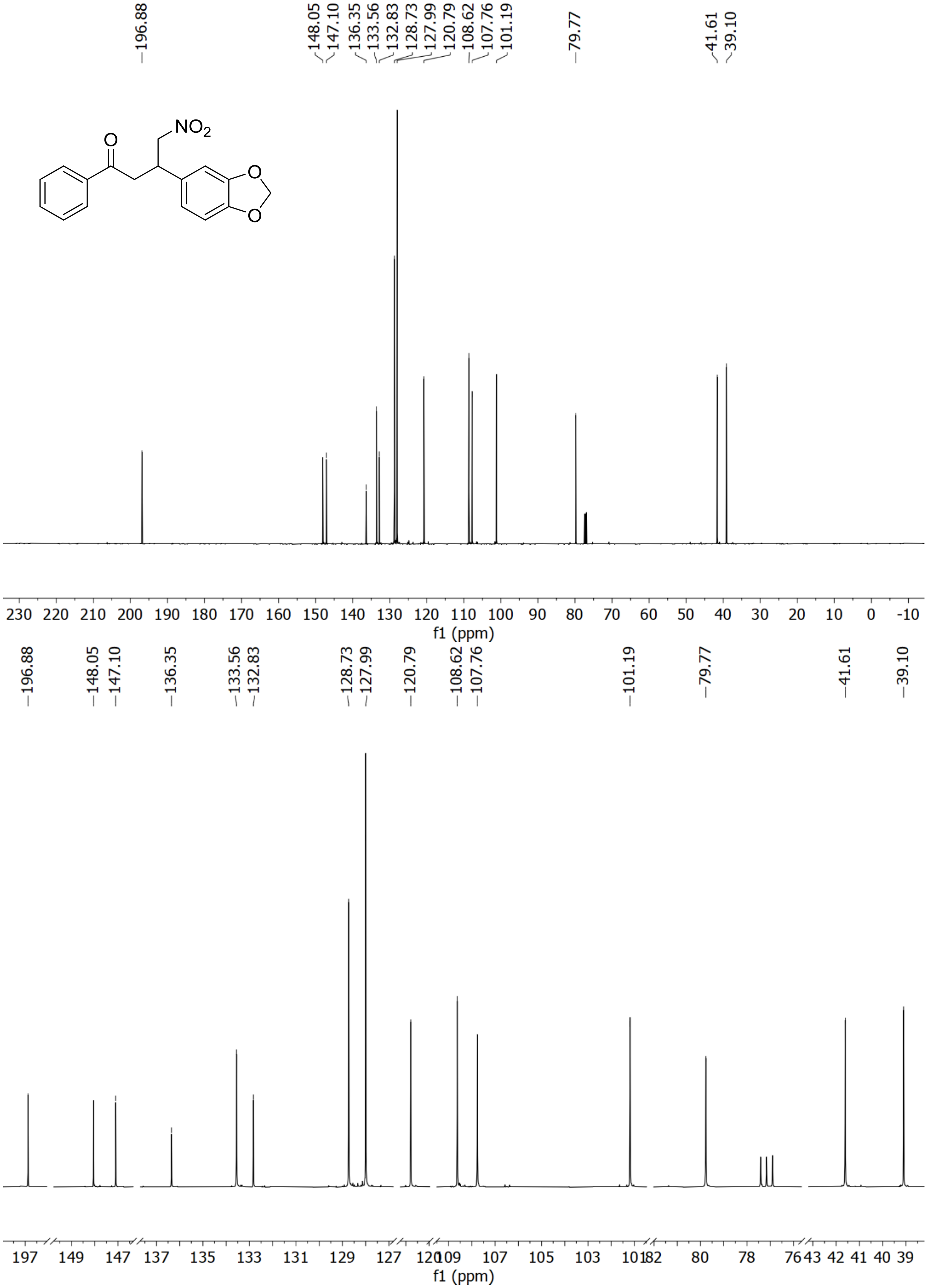

Figure S23: *^{13}C NMR spectrum (125 MHz, $CDCl_3$) for the pure product of 3-(benzo[d][1,3]dioxol-5-yl)-4-nitro-1-phenylbutan-1-one (2i): full scale spectrum (top) and spectrum expansions (bottom).*

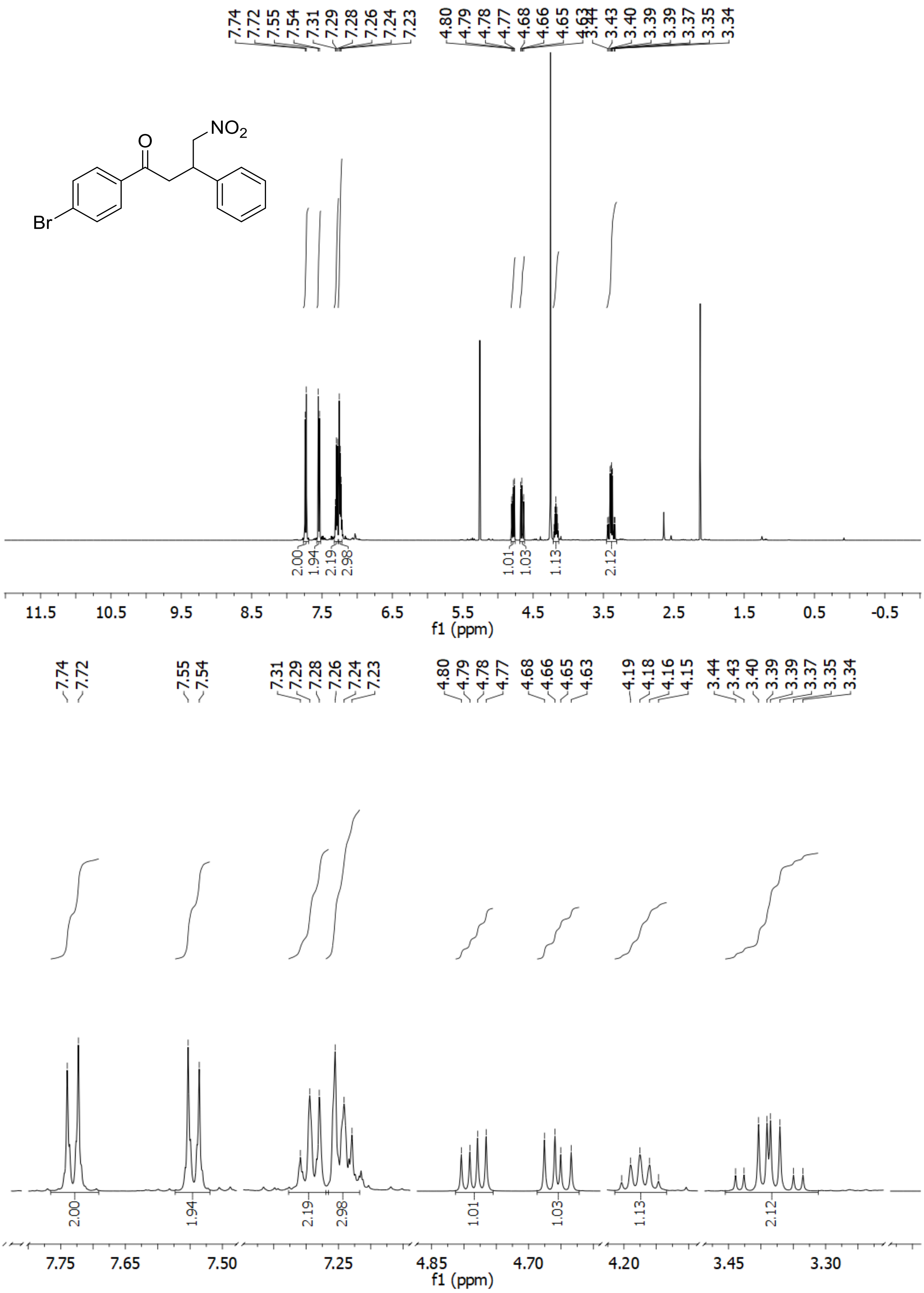

Figure S24: *^{1}H-NMR spectrum (500 MHz, $CDCl_3$) for the pure product of 1-(4-bromophenyl)-4-nitro-3-phenylbutan-1-one (2j): full scale spectrum (top) and spectrum expansions (bottom).*

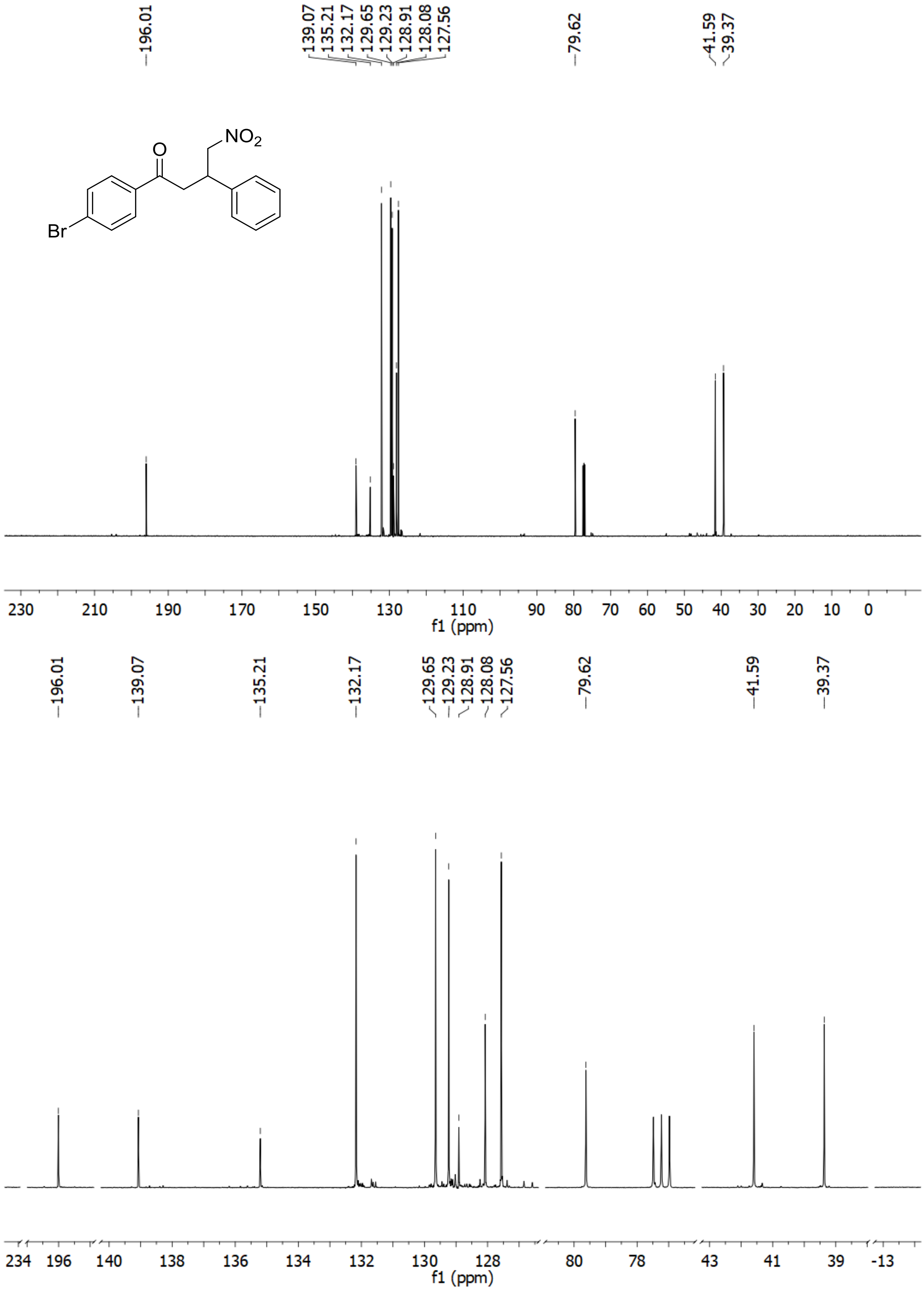

Figure S25: *^{13}C NMR spectrum (125 MHz, $CDCl_3$) for the pure product of 1-(4-bromophenyl)-4-nitro-3-phenylbutan-1-one (2j): full scale spectrum (top) and spectrum expansions (bottom).*

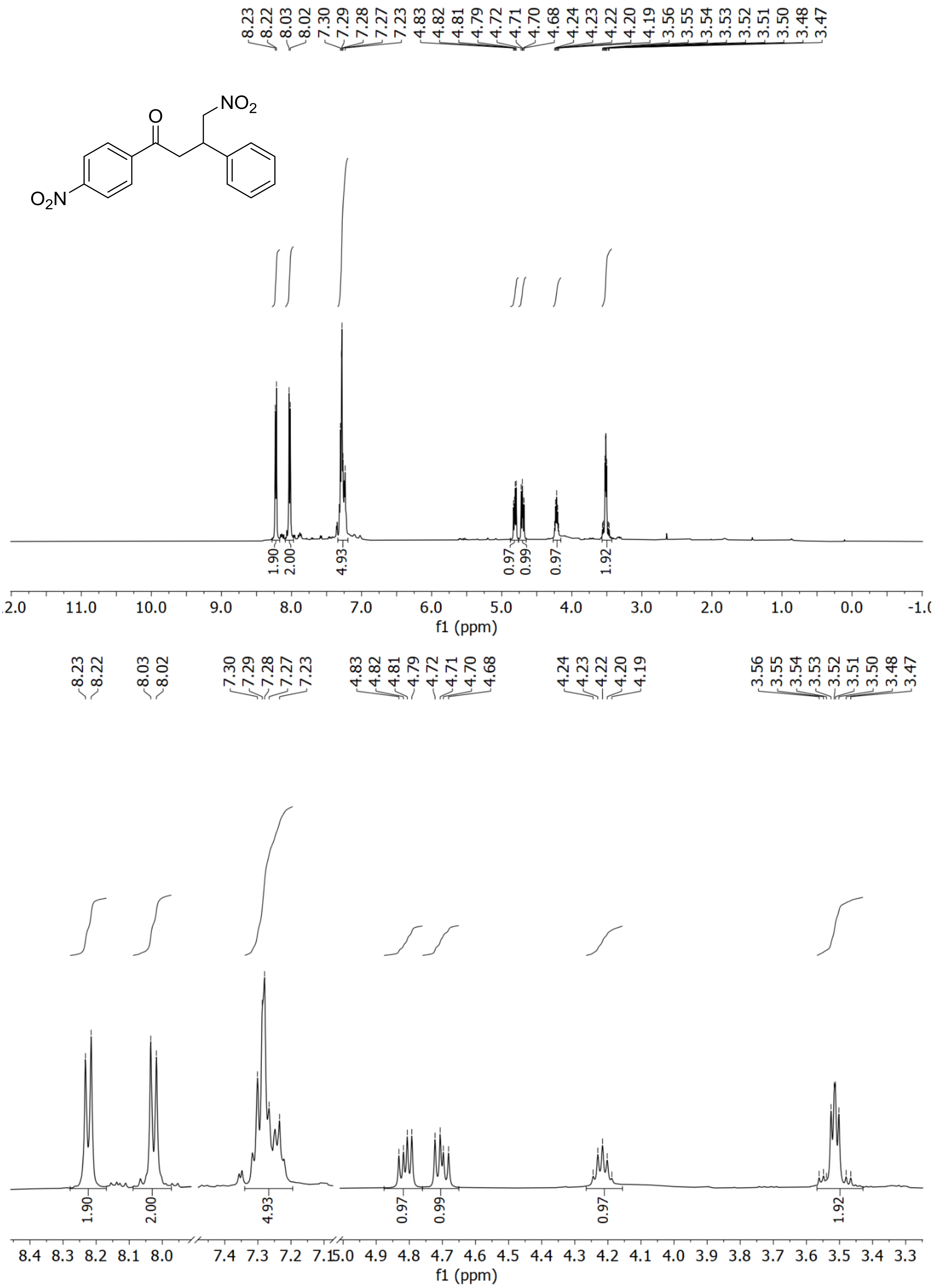

Figure S26: *^{1}H-NMR spectrum (500 MHz, $CDCl_3$) for the pure product of 4-nitro-1-(4-nitrophenyl)-3-phenylbutan-1-one (2k): full scale spectrum (top) and spectrum expansions (bottom).*

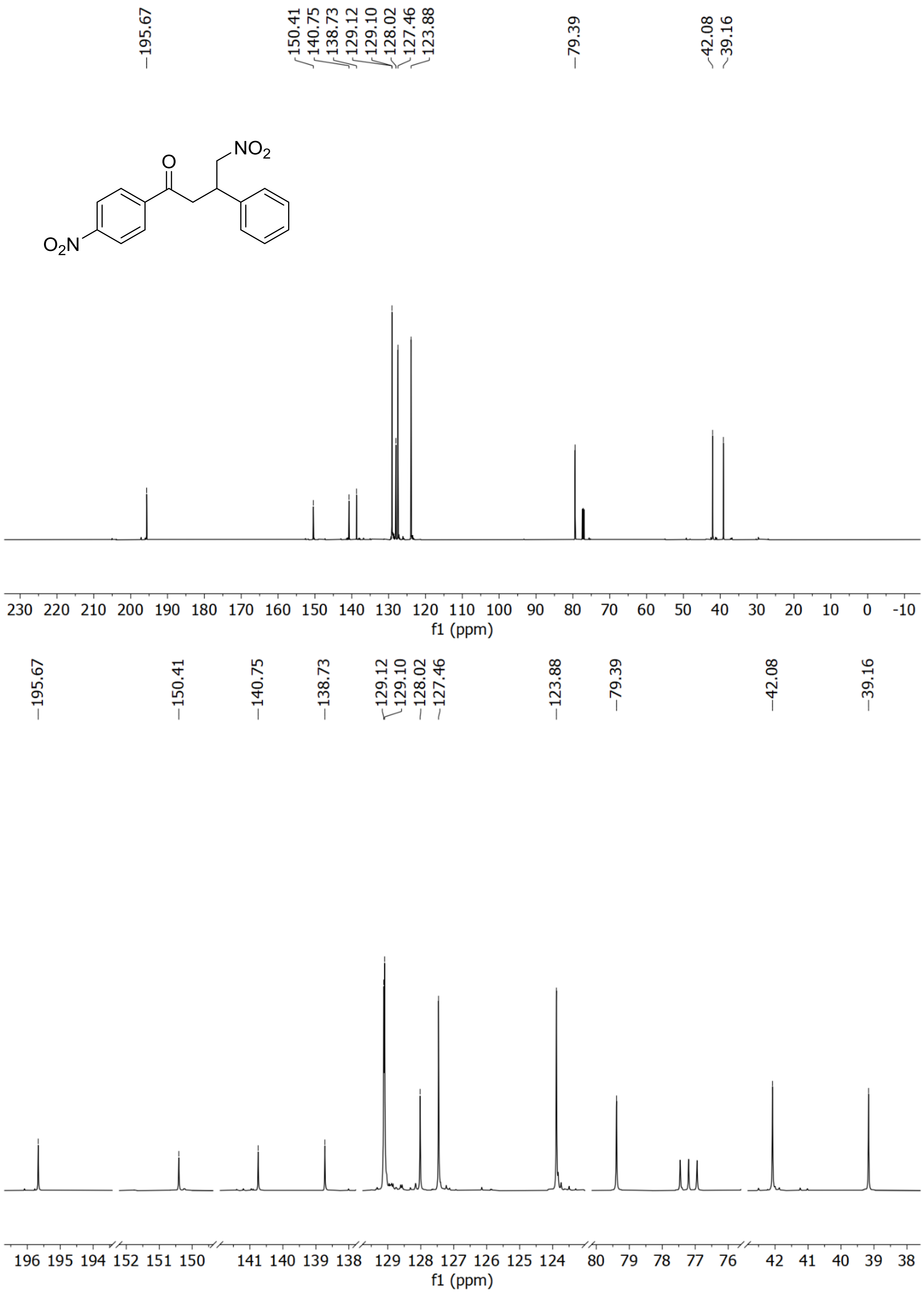

Figure S27: *^{13}C NMR spectrum (125 MHz, $CDCl_3$) for the pure product of 4-nitro-1-(4-nitrophenyl)-3-phenylbutan-1-one (2k): full scale spectrum (top) and spectrum expansions (bottom).*

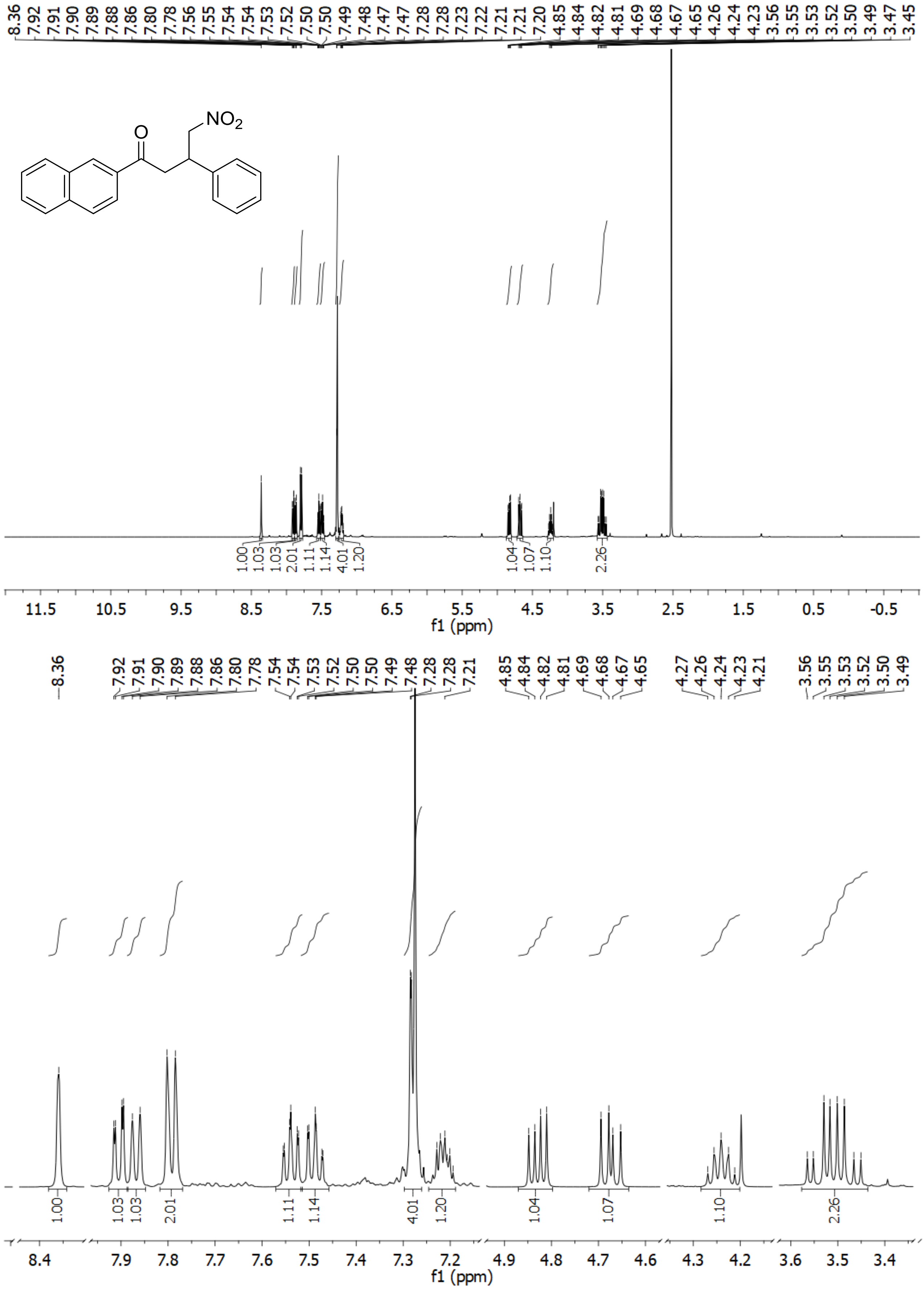

Figure S28: *^{1}H-NMR spectrum (500 MHz, $CDCl_3$) for the pure product of 1-(naphthalen-2-yl)-4-nitro-3-phenylbutan-1-one (2l): full scale spectrum (top) and spectrum expansions (bottom).*

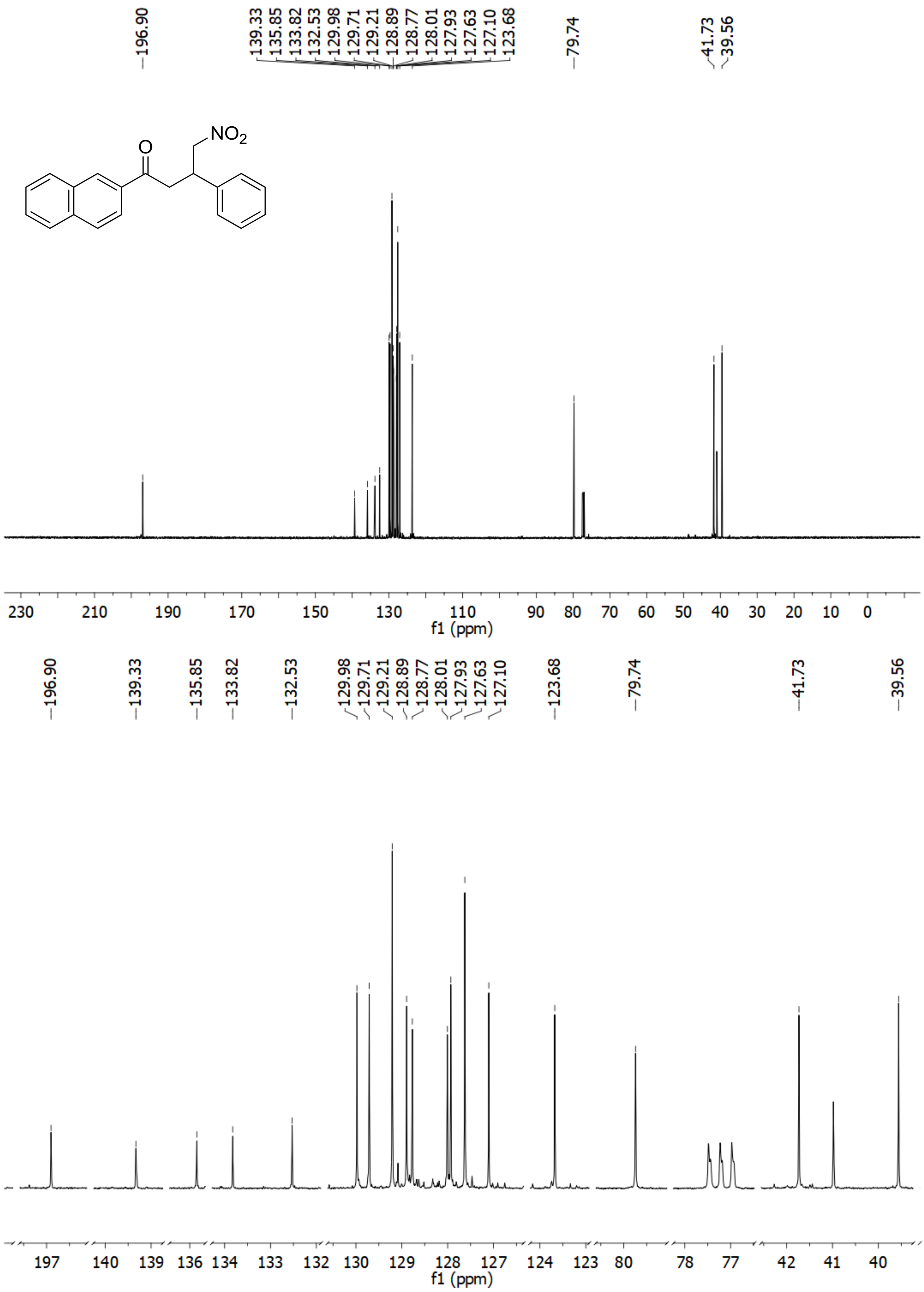

Figure S29: *^{13}C NMR spectrum (125 MHz, $CDCl_3$) for the pure product of 1-(naphthalen-2-yl)-4-nitro-3-phenylbutan-1-one (2l): full scale spectrum (top) and spectrum expansions (bottom).*

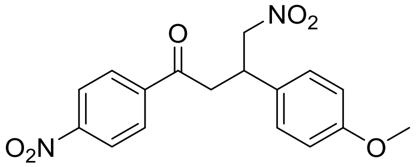

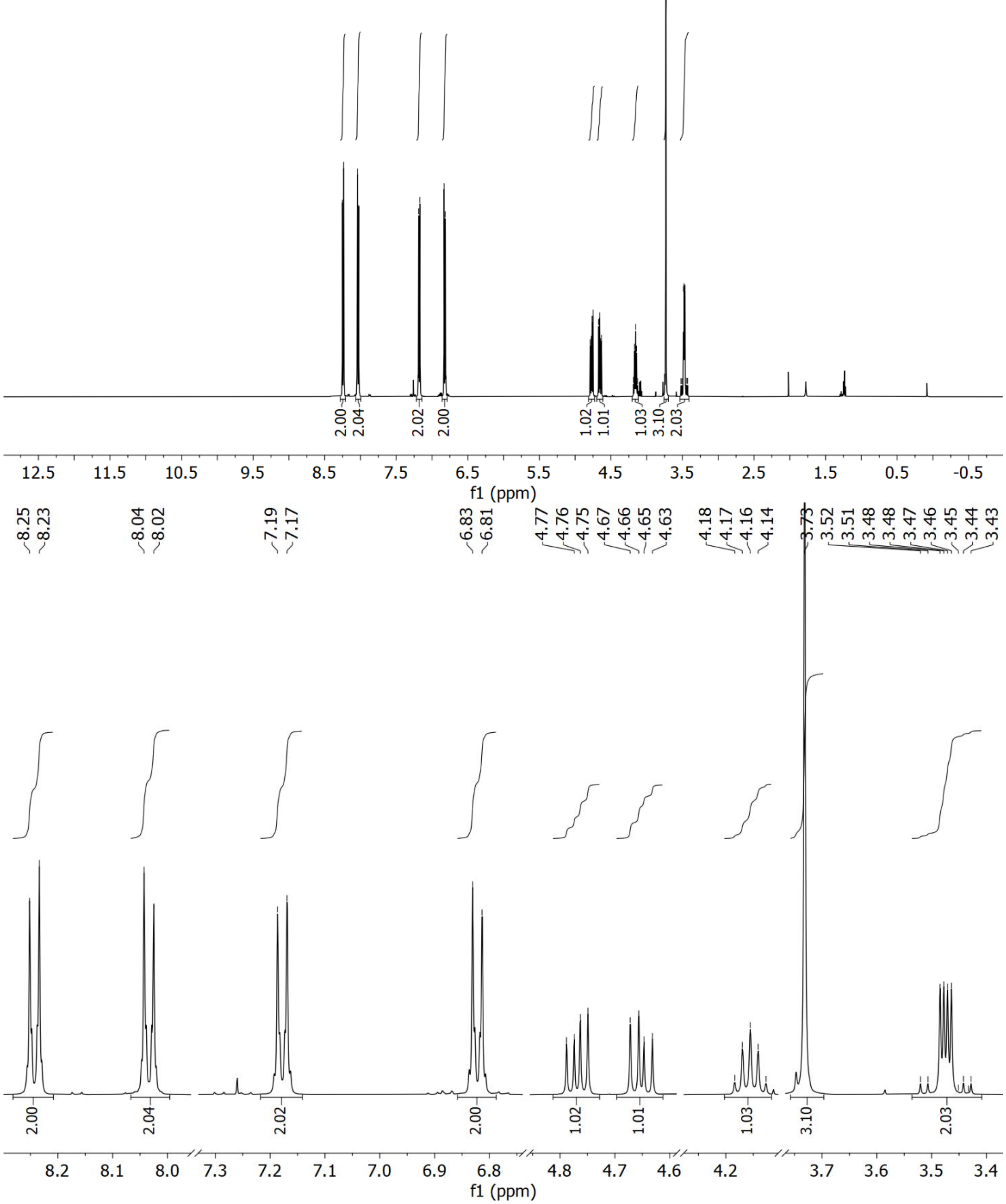

Figure S30: *^{1}H-NMR spectrum (500 MHz, $CDCl_3$) for the pure product of 3-(4-methoxyphenyl)-4-nitro-1-(4-nitrophenyl)butan-1-one (2m): full scale spectrum (top) and spectrum expansions (bottom).*

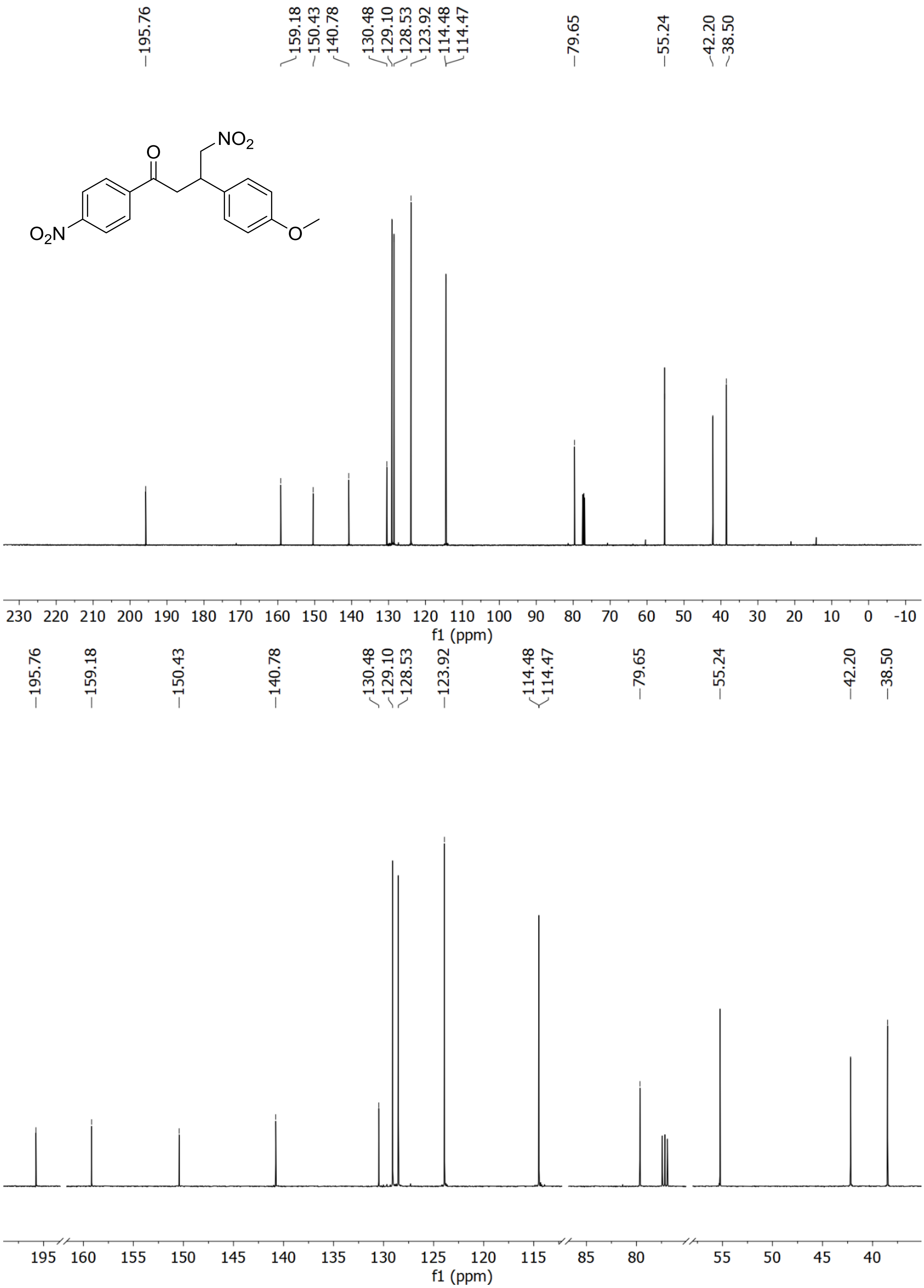

Figure S31: *^{13}C NMR spectrum (125 MHz, $CDCl_3$) for the pure product of 3-(4-methoxyphenyl)-4-nitro-1-(4-nitrophenyl)butan-1-one (2m): full scale spectrum (top) and spectrum expansions (bottom).*

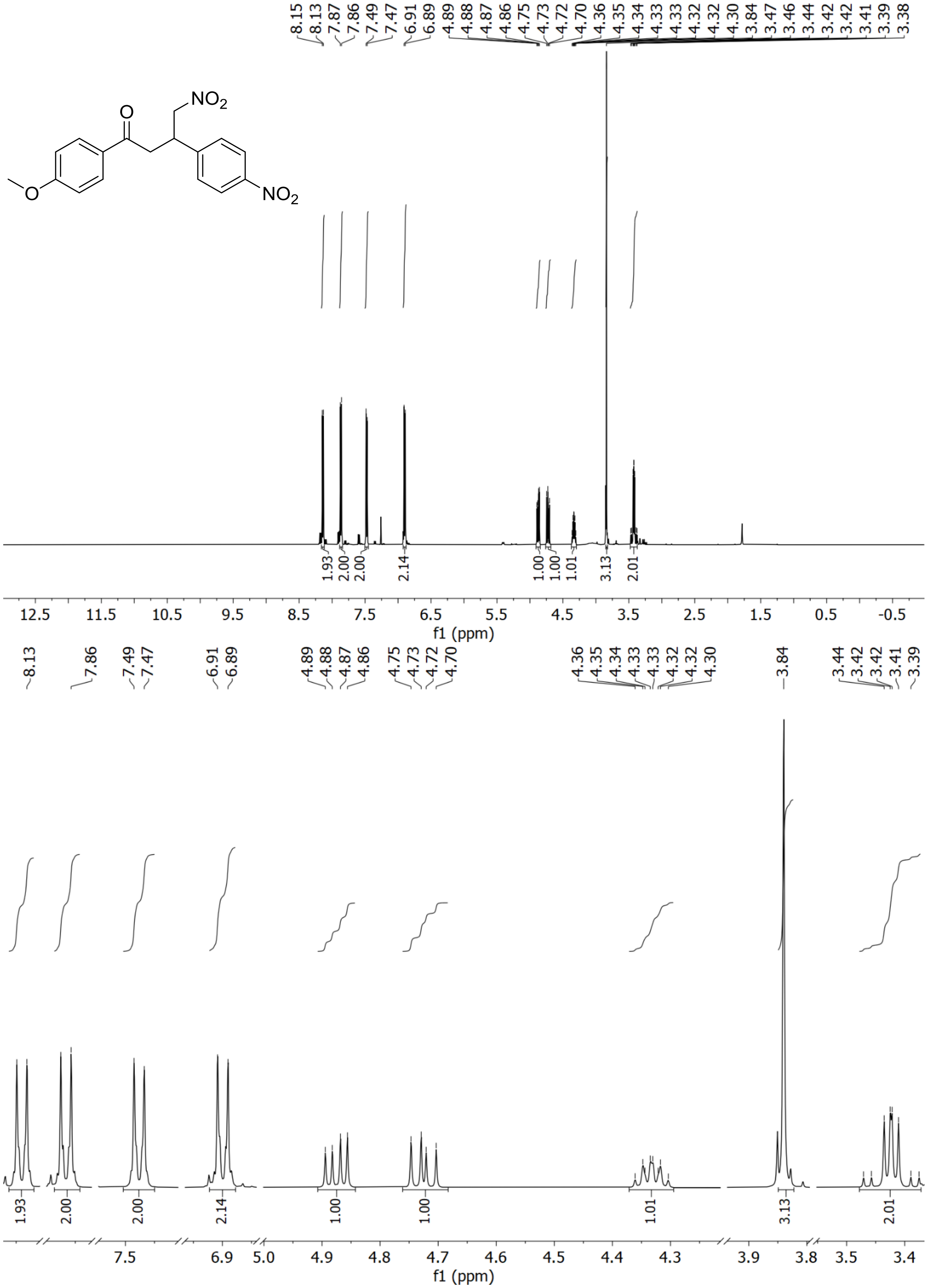

Figure S32: *^{1}H-NMR spectrum (500 MHz, $CDCl_3$) for the pure product of 1-(4-methoxyphenyl)-4-nitro-3-(4-nitrophenyl)butan-1-one (2n): full scale spectrum (top) and spectrum expansions (bottom).*

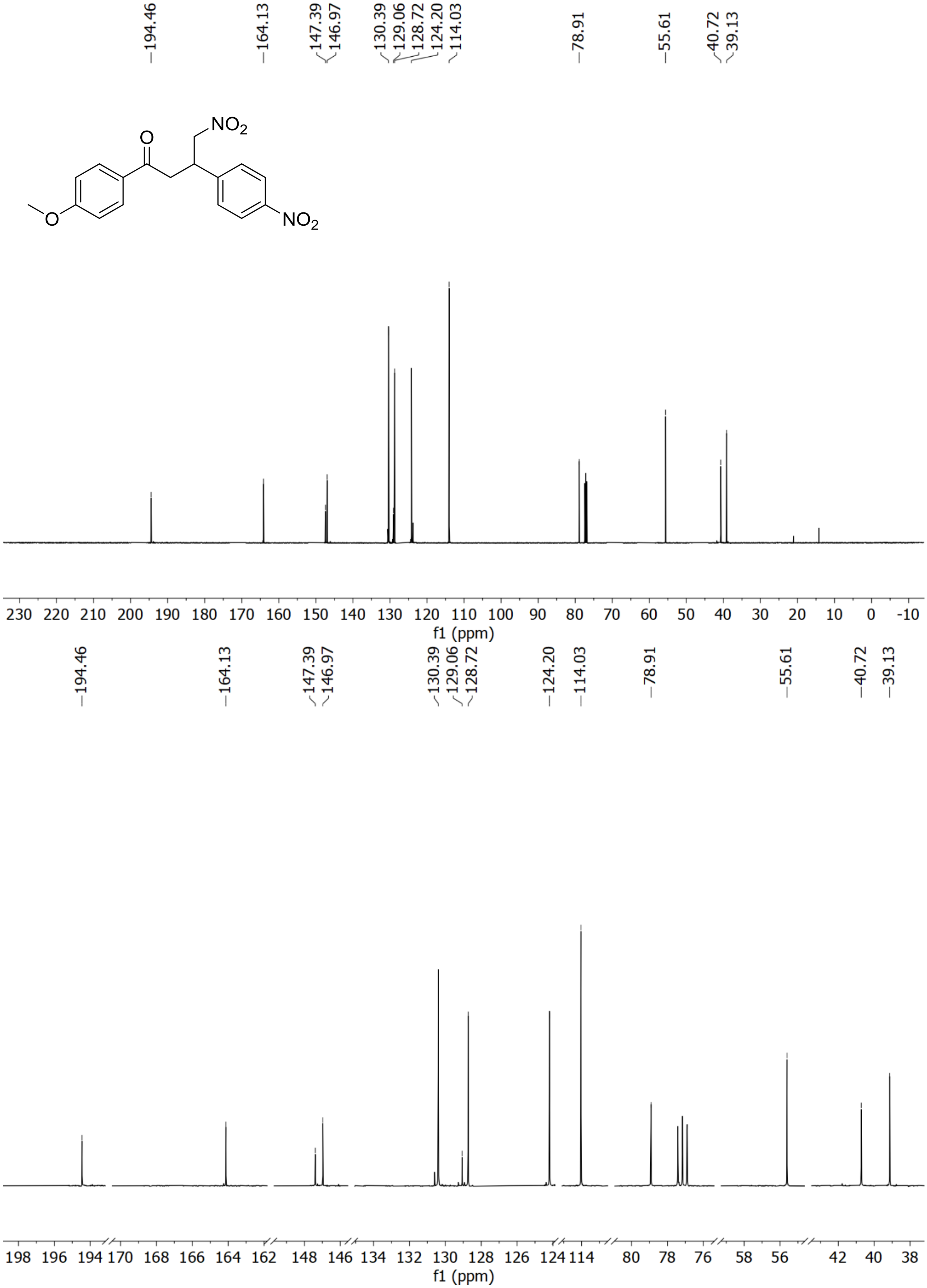

Figure S33: *^{13}C NMR spectrum (125 MHz, $CDCl_3$) for the pure product of 1-(4-methoxyphenyl)-4-nitro-3-(4-nitrophenyl)butan-1-one (2n): full scale spectrum (top) and spectrum expansions (bottom).*

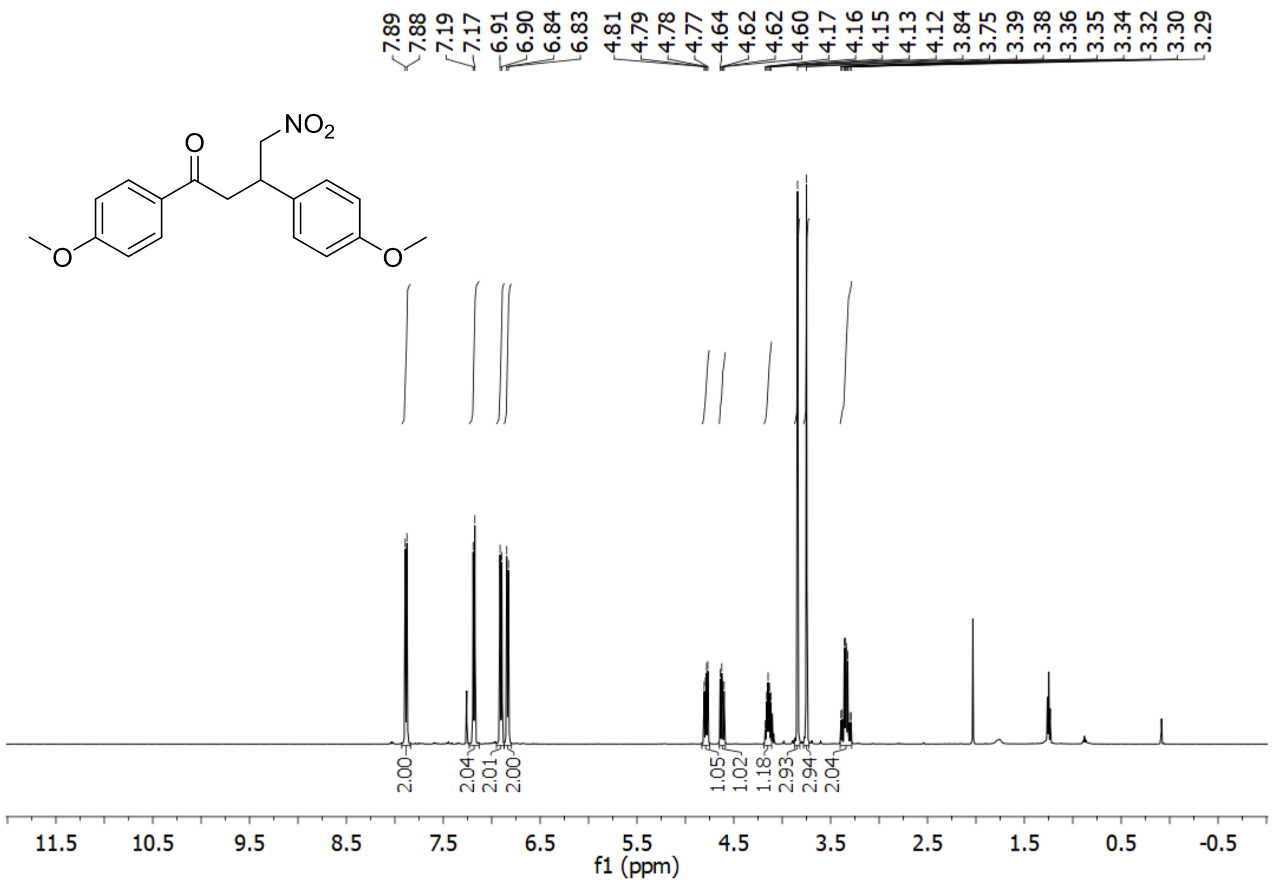

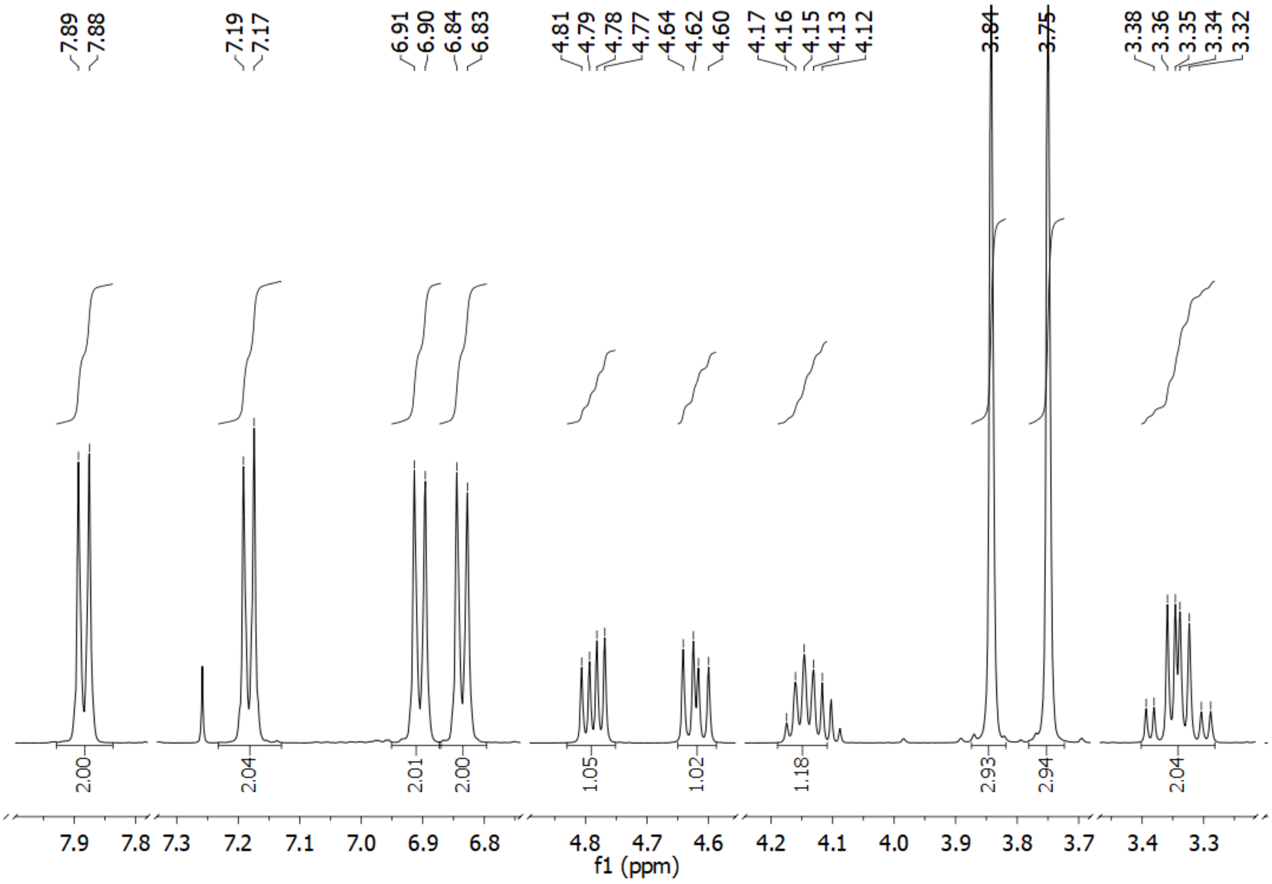

Figure S34: ^{1}H-NMR spectrum (500 MHz, $CDCl_3$) for the pure product of 1,3-bis(4-methoxyphenyl)-4-nitrobutan-1-one (2o): full scale spectrum (top) and spectrum expansions (bottom).

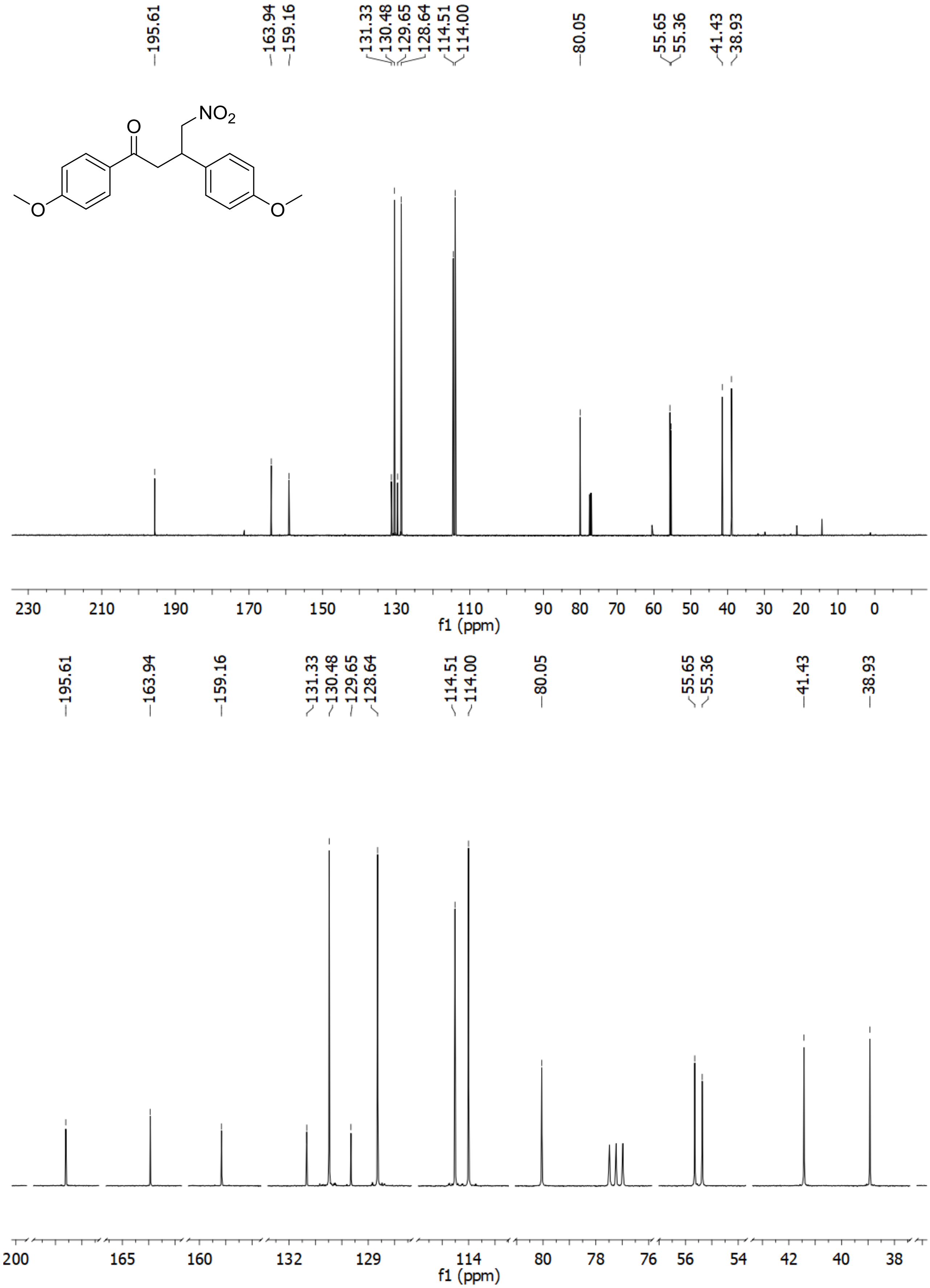

Figure S35: *^{13}C NMR spectrum (125 MHz, $CDCl_3$) for the pure product of 1,3-bis(4-methoxyphenyl)-4-nitrobutan-1-one (2o): full scale spectrum (top) and spectrum expansions (bottom).*

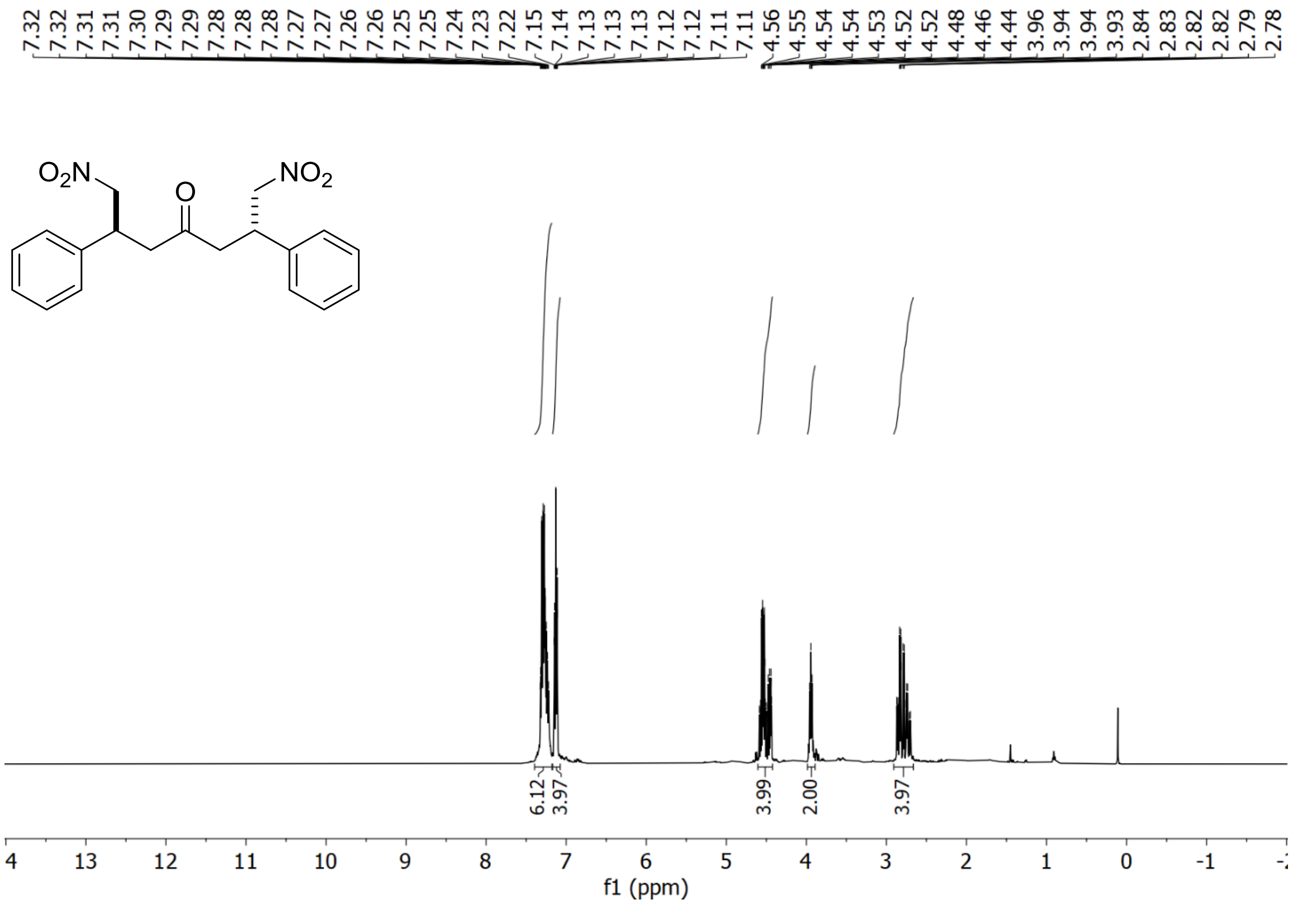

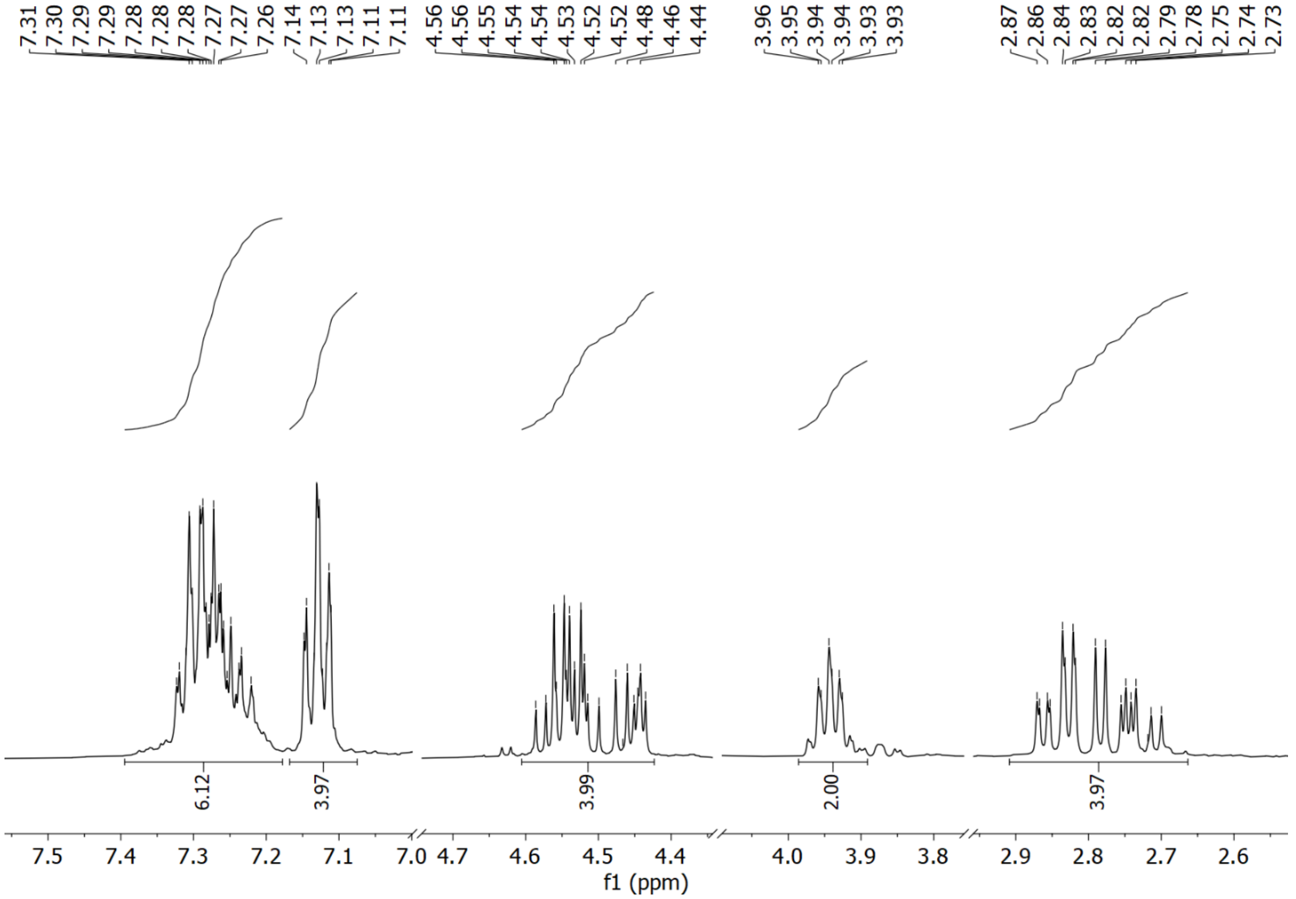

Figure S36: *^{1}H-NMR spectrum (500 MHz, $CDCl_3$) for the pure product 1,7-dinitro-2,6-diphenylheptan-4-one (5p): full scale spectrum (top) and spectrum expansions (bottom).*

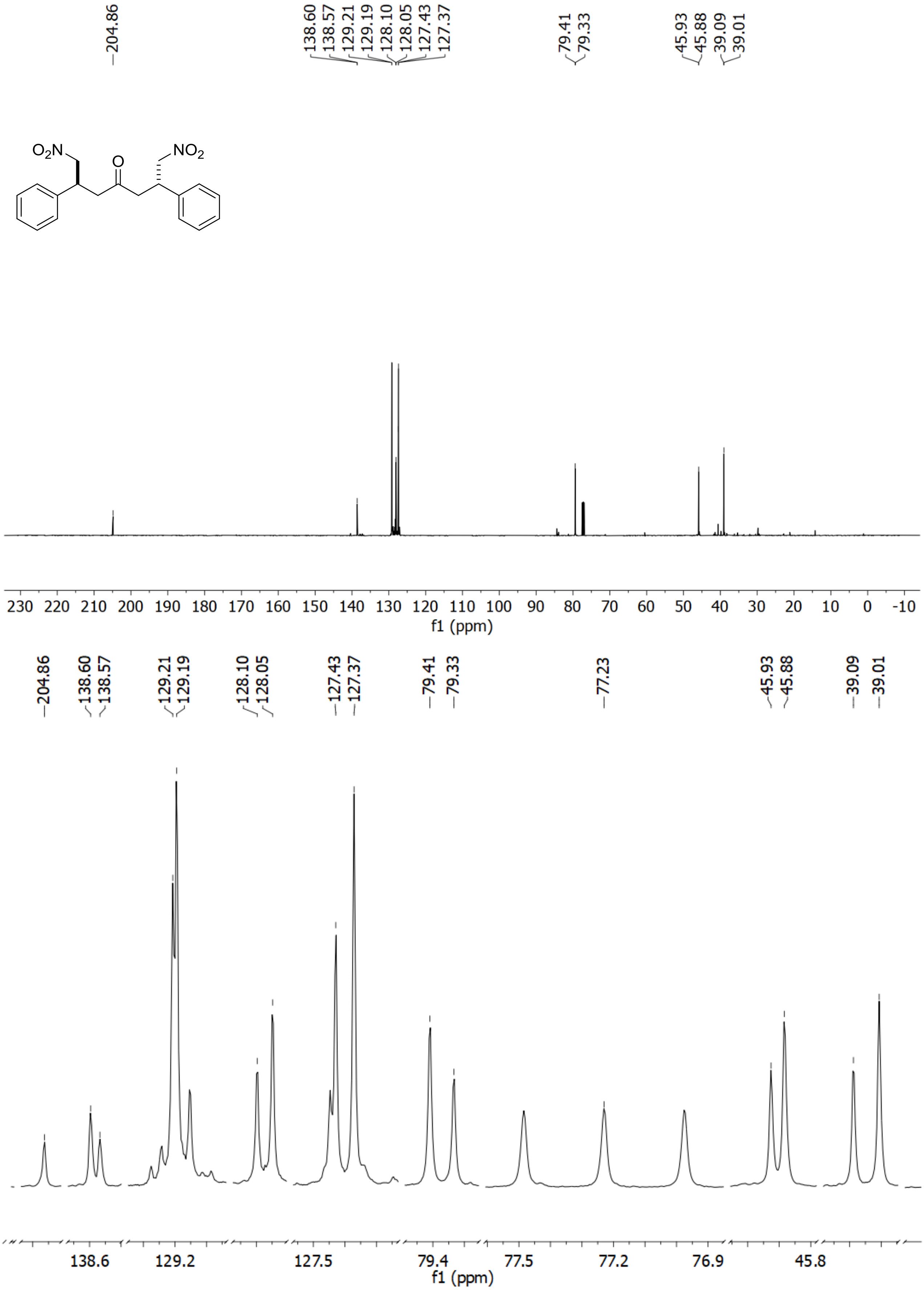

Figure S37: *^{13}C-NMR spectrum (125 MHz, $CDCl_3$) for the pure product 1,7-dinitro-2,6-diphenylheptan-4-one (5p): full scale spectrum (top) and spectrum expansions (bottom).*

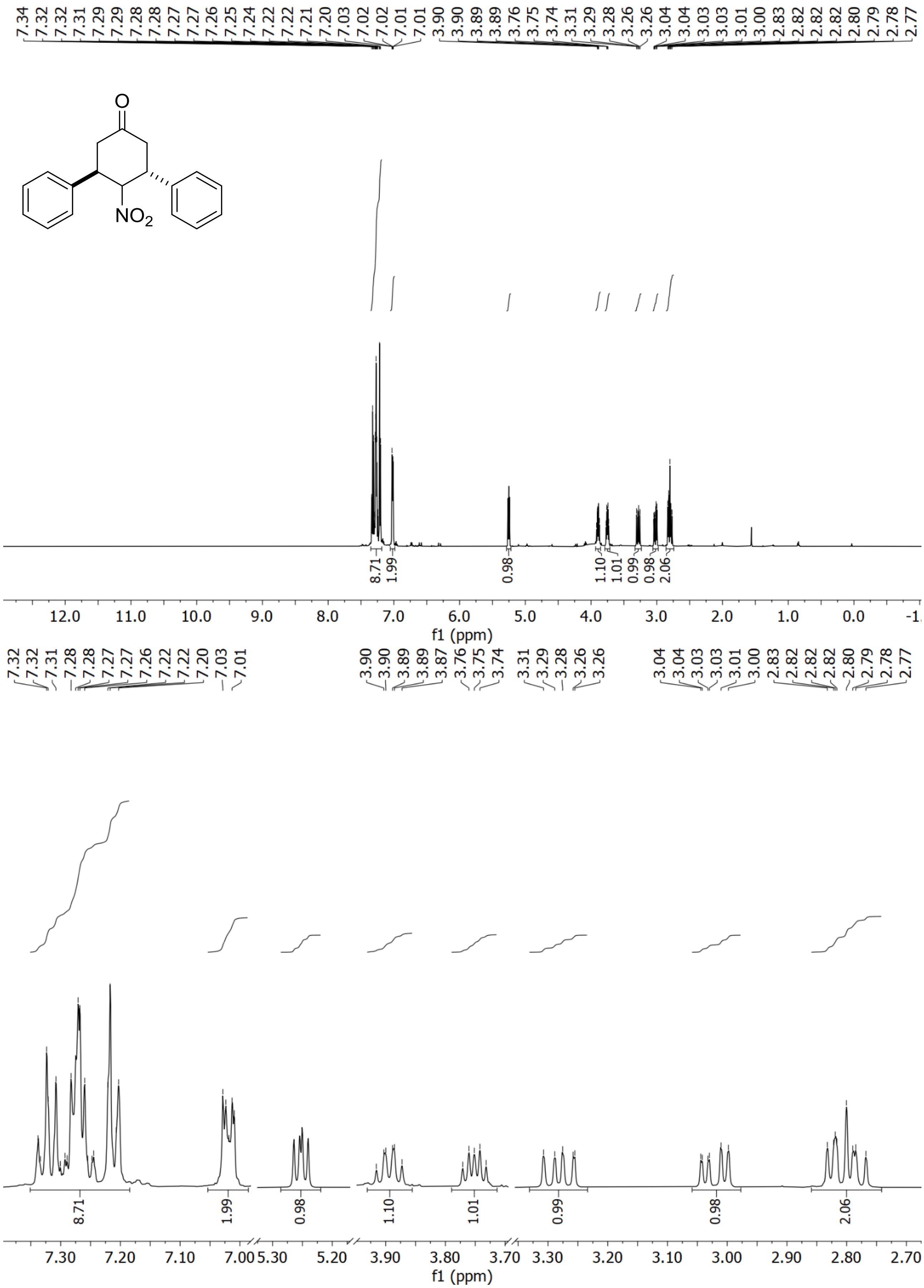

Figure S38: *^{1}H-NMR spectrum (500 MHz, $CDCl_3$) for the pure product anti-4-nitro-3,5-diphenylcyclohexan-1-one (6p): full scale spectrum (top) and spectrum expansions (bottom).*

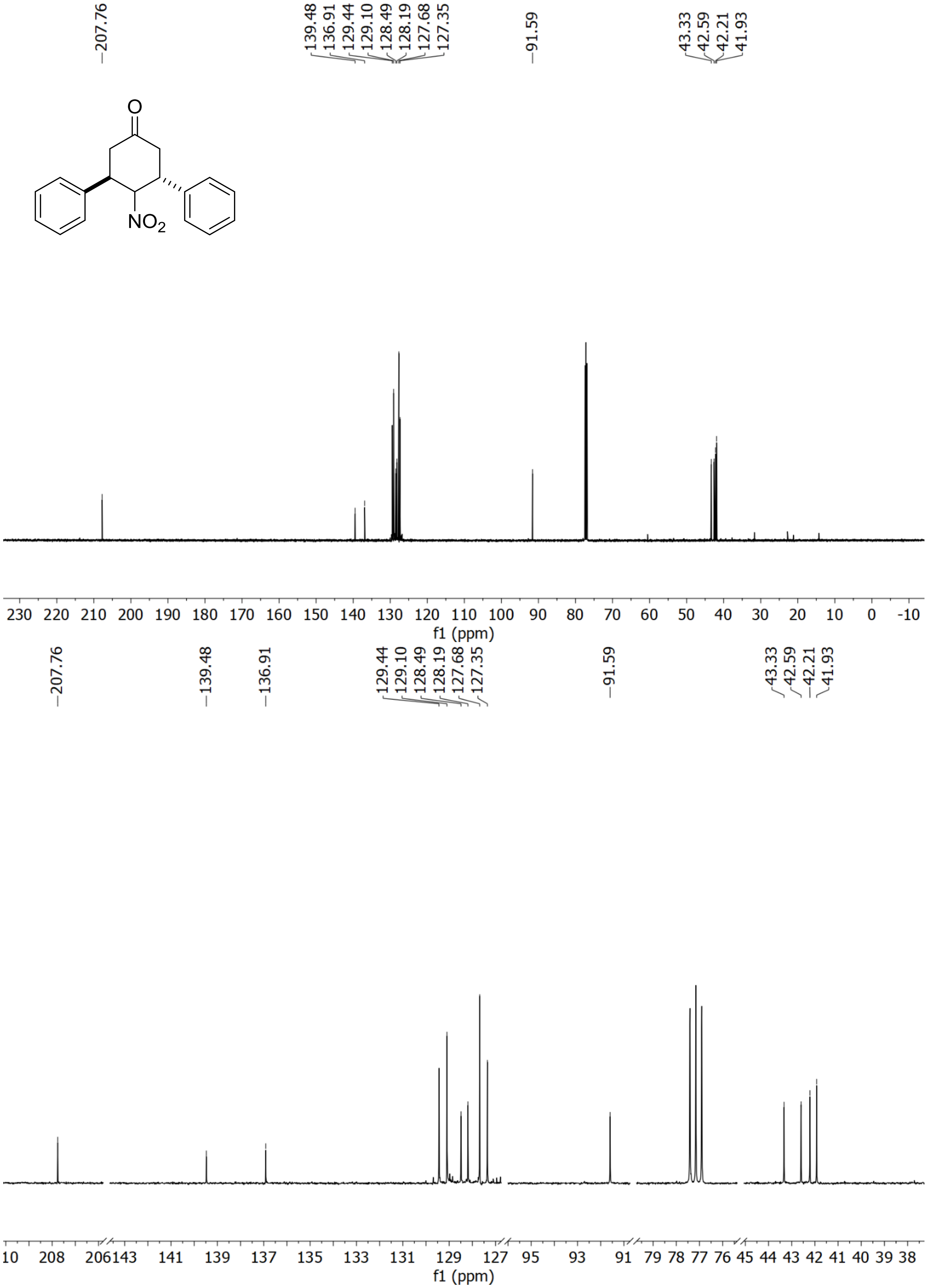

Figure S39: *^{13}C-NMR spectrum (125 MHz, $CDCl_3$) for the pure product anti-4-nitro-3,5-diphenylcyclohexan-1-one (6p): full scale spectrum (top) and spectrum expansions (bottom).*

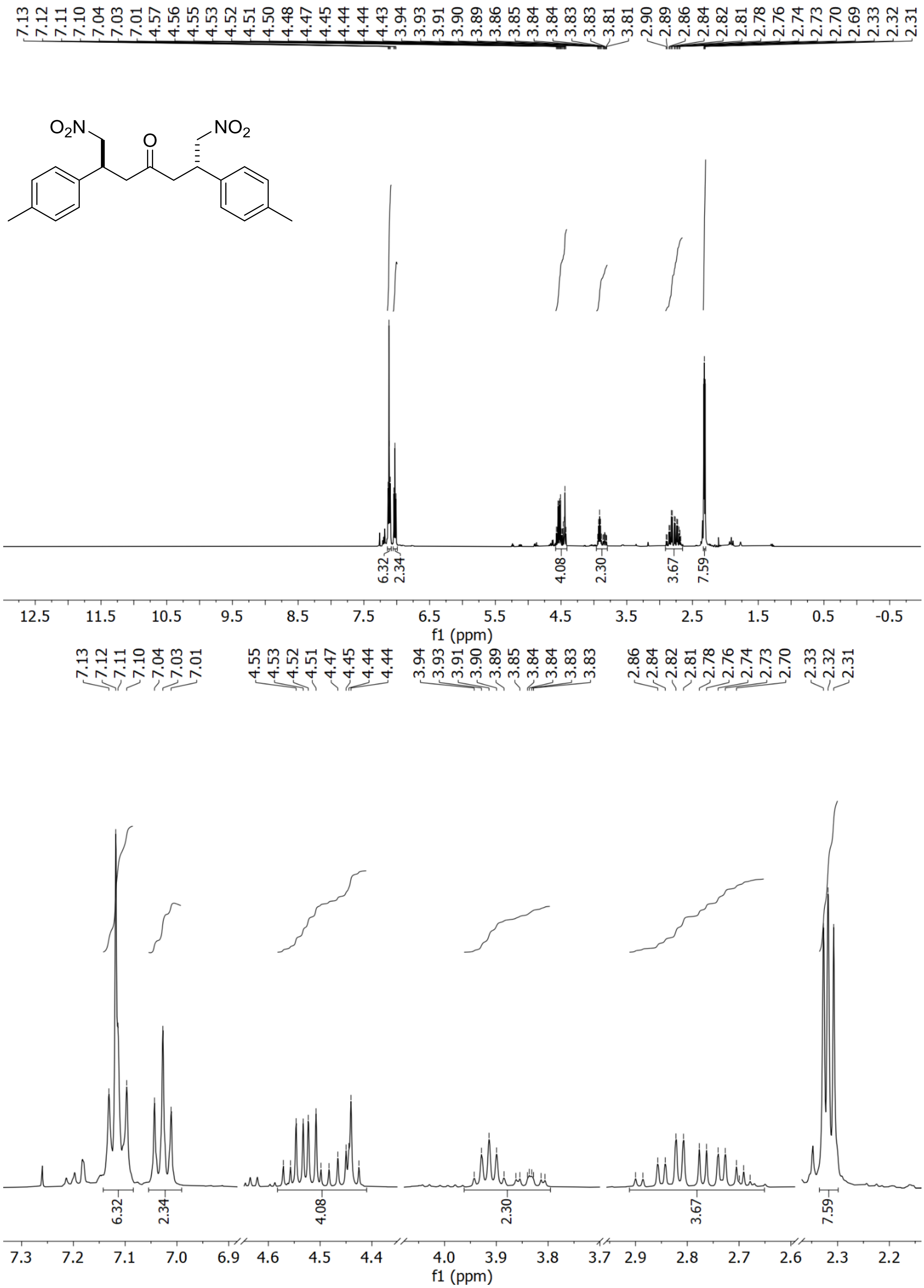

Figure S40: *^{1}H-NMR spectrum (500 MHz, $CDCl_3$) for the pure product anti-1,7-dinitro-2,6-di-p-tolylheptan-4-one (5q): full scale spectrum (top) and spectrum expansions (bottom).*

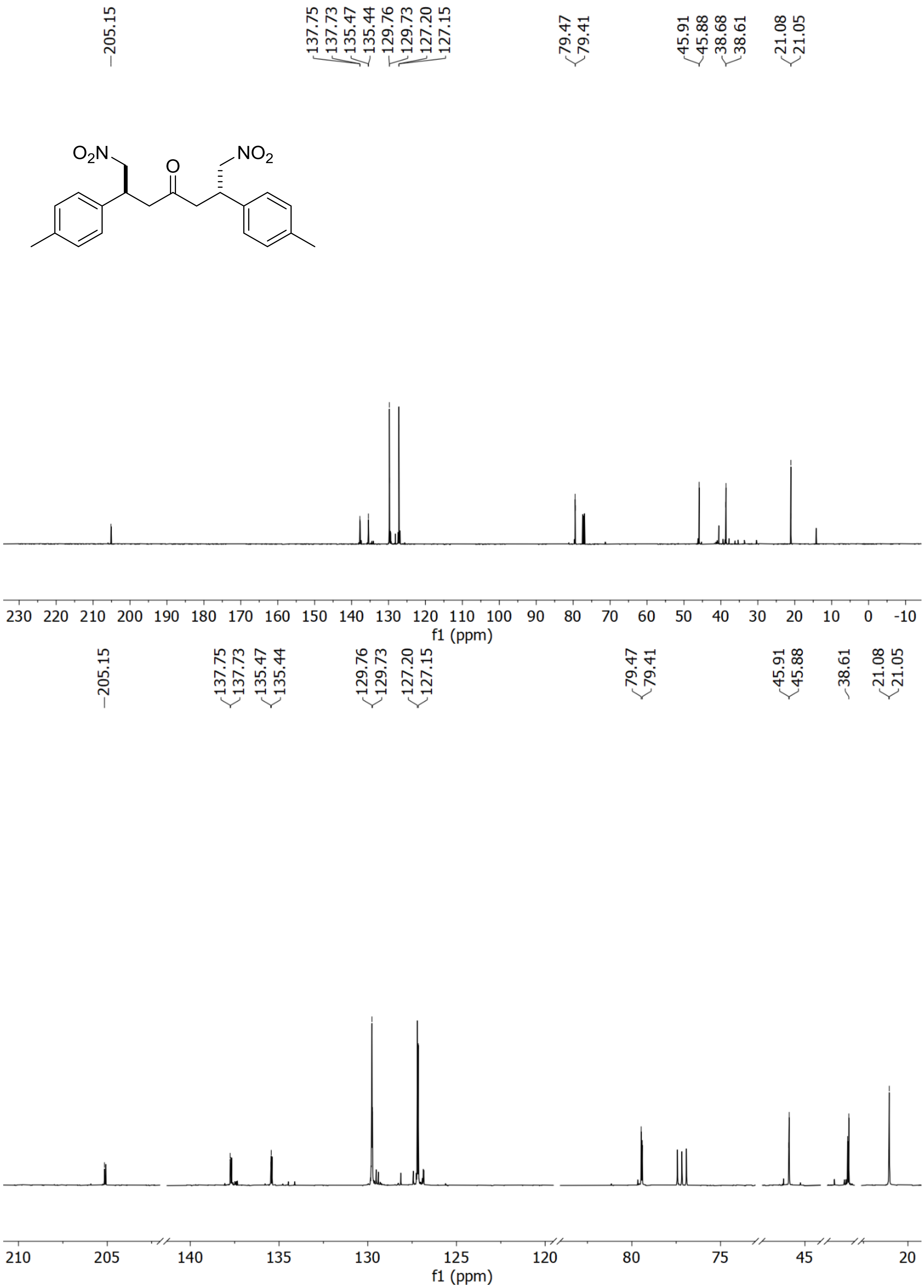

Figure S41: *^{13}C-NMR spectrum (125 MHz, $CDCl_3$) for the pure product anti-1,7-dinitro-2,6-di-p-tolylheptan-4-one (5q): full scale spectrum (top) and spectrum expansions (bottom).*

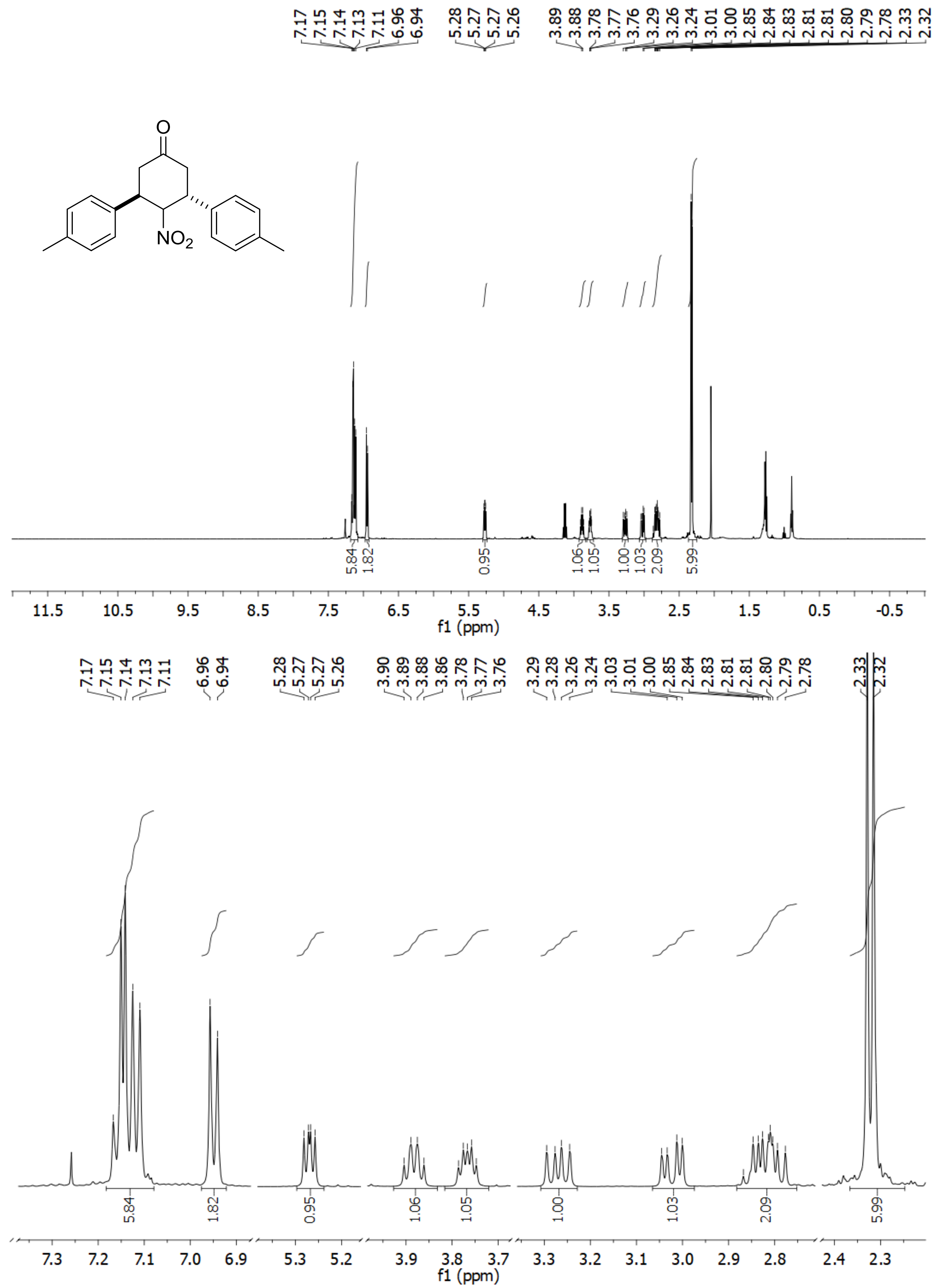

Figure S42: *^{1}H-NMR spectrum (500 MHz, $CDCl_3$) for the pure product anti-4-nitro-3,5-di-p-tolylcyclohexan-1-one (6q): full scale spectrum (top) and spectrum expansions (bottom).*

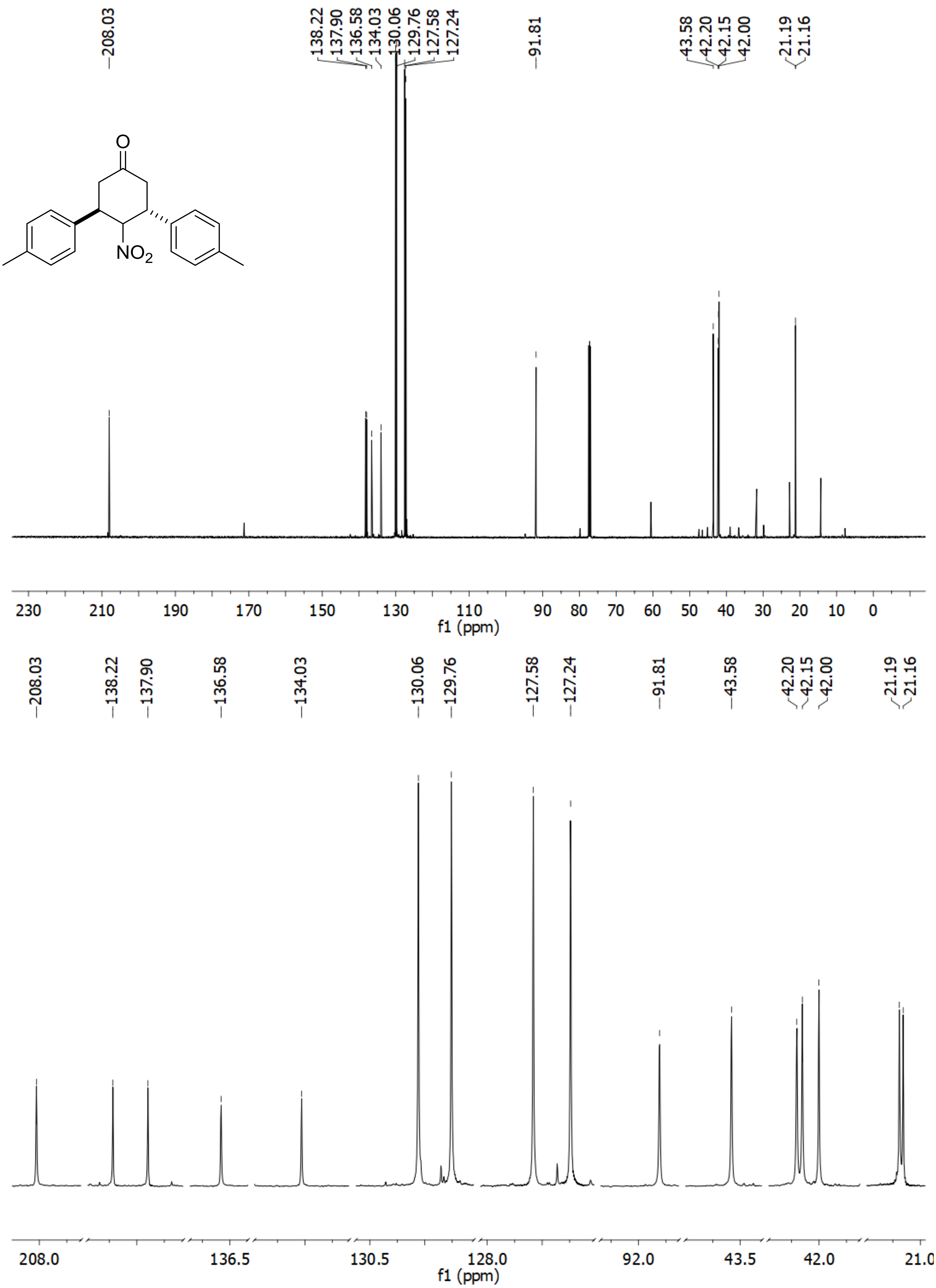

Figure S43: *^{13}C-NMR spectrum (125 MHz, $CDCl_3$) for the pure product anti-4-nitro-3,5-di-p-tolylcyclohexan-1-one (6q): full scale spectrum (top) and spectrum expansions (bottom).*

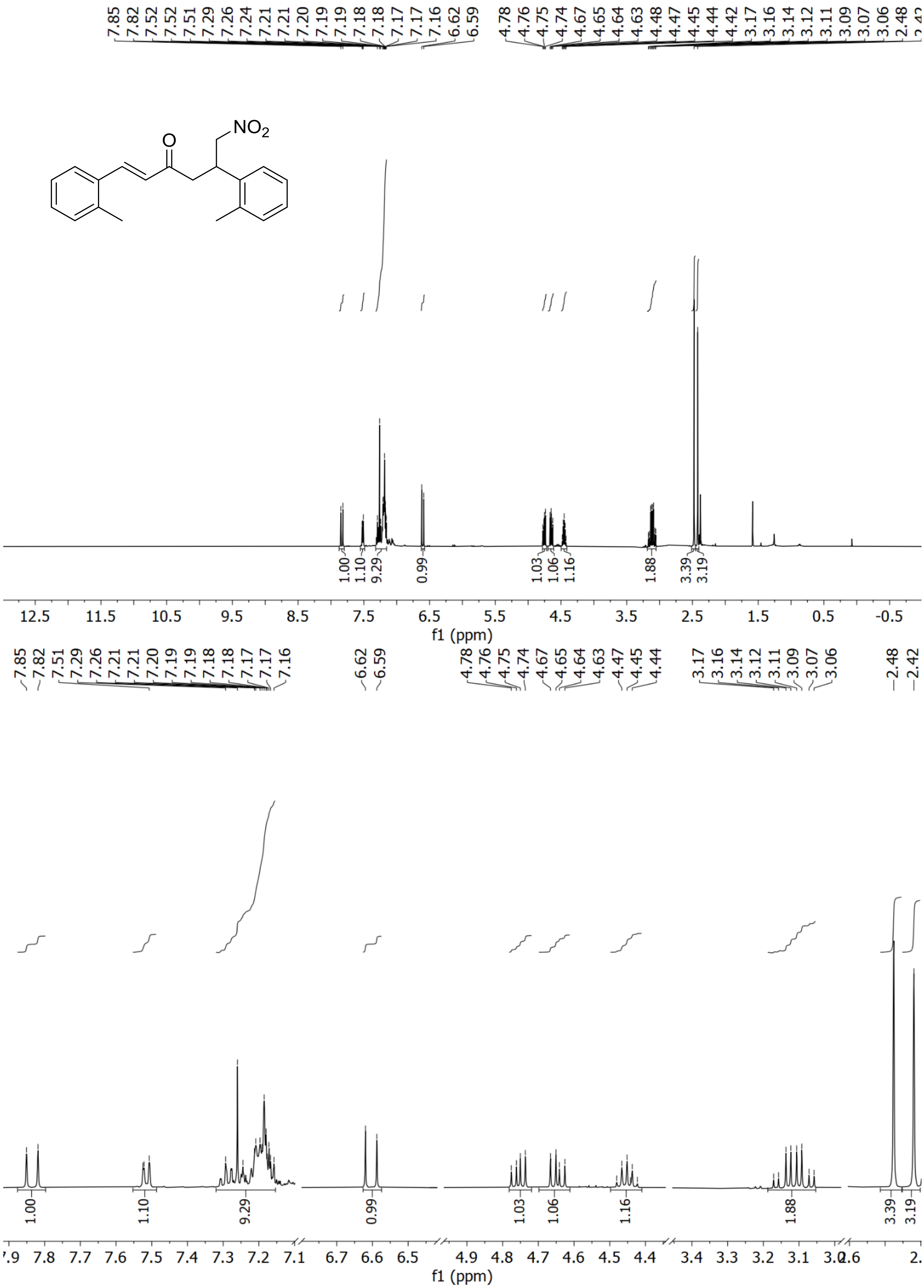

Figure S44: *^{1}H-NMR spectrum (500 MHz, $CDCl_3$) for the pure product (E)-6-nitro-1,5-di-o-tolylhex-1-en-3-one (4s): full scale spectrum (top) and spectrum expansions (bottom).*

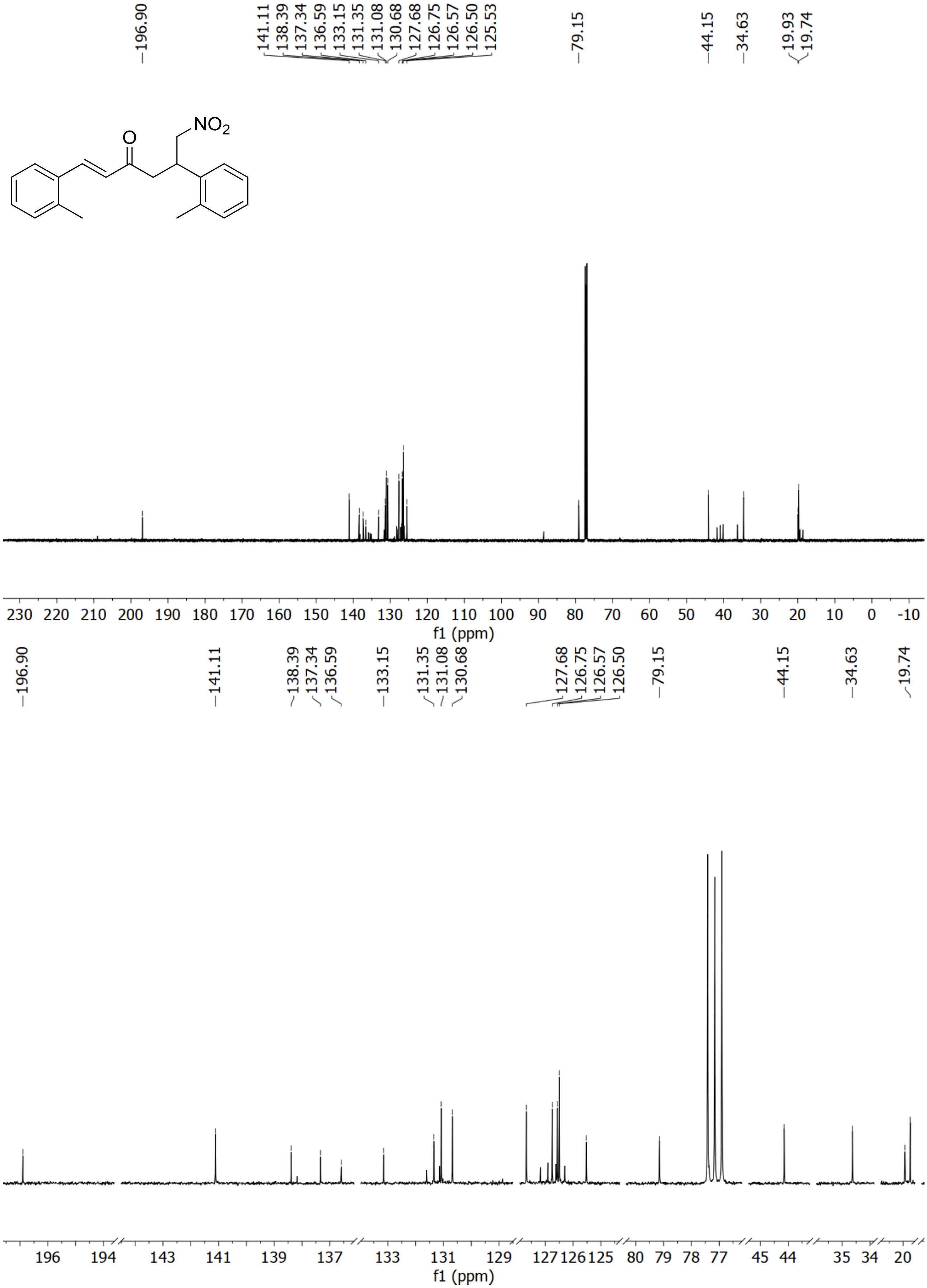

Figure S45: *^{13}C-NMR spectrum (125 MHz, $CDCl_3$) for the pure product (E)-6-nitro-1,5-di-o-tolylhex-1-en-3-one (4s): full scale spectrum (top) and spectrum expansions (bottom).*

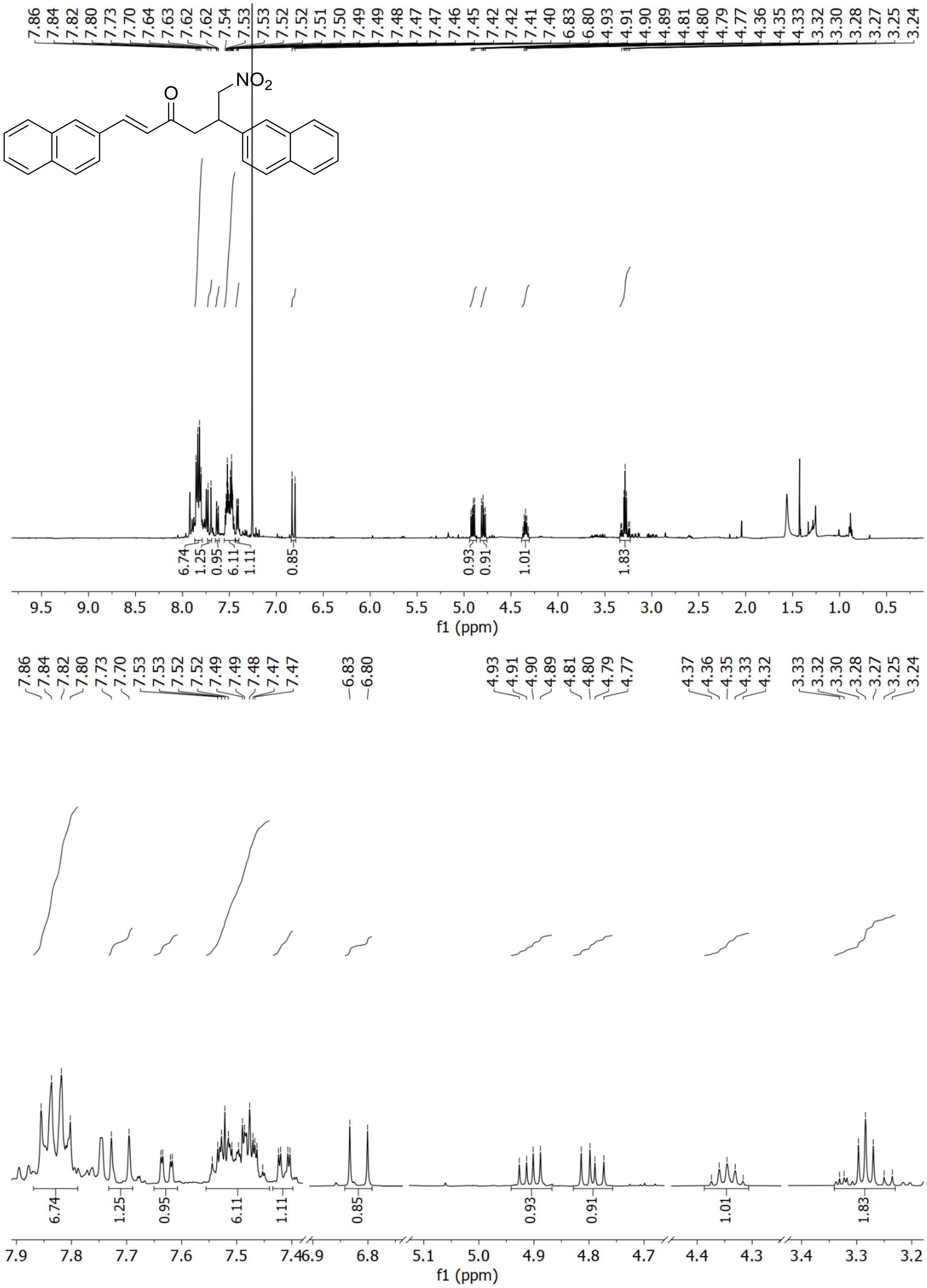

Figure S46: *^{1}H-NMR spectrum (500 MHz, CDCl$_3$) for the pure product (E)-1,5-di(naphthalen-2-yl)-6-nitrohex-1-en-3-one (4t): full scale spectrum (top) and spectrum expansions (bottom).*

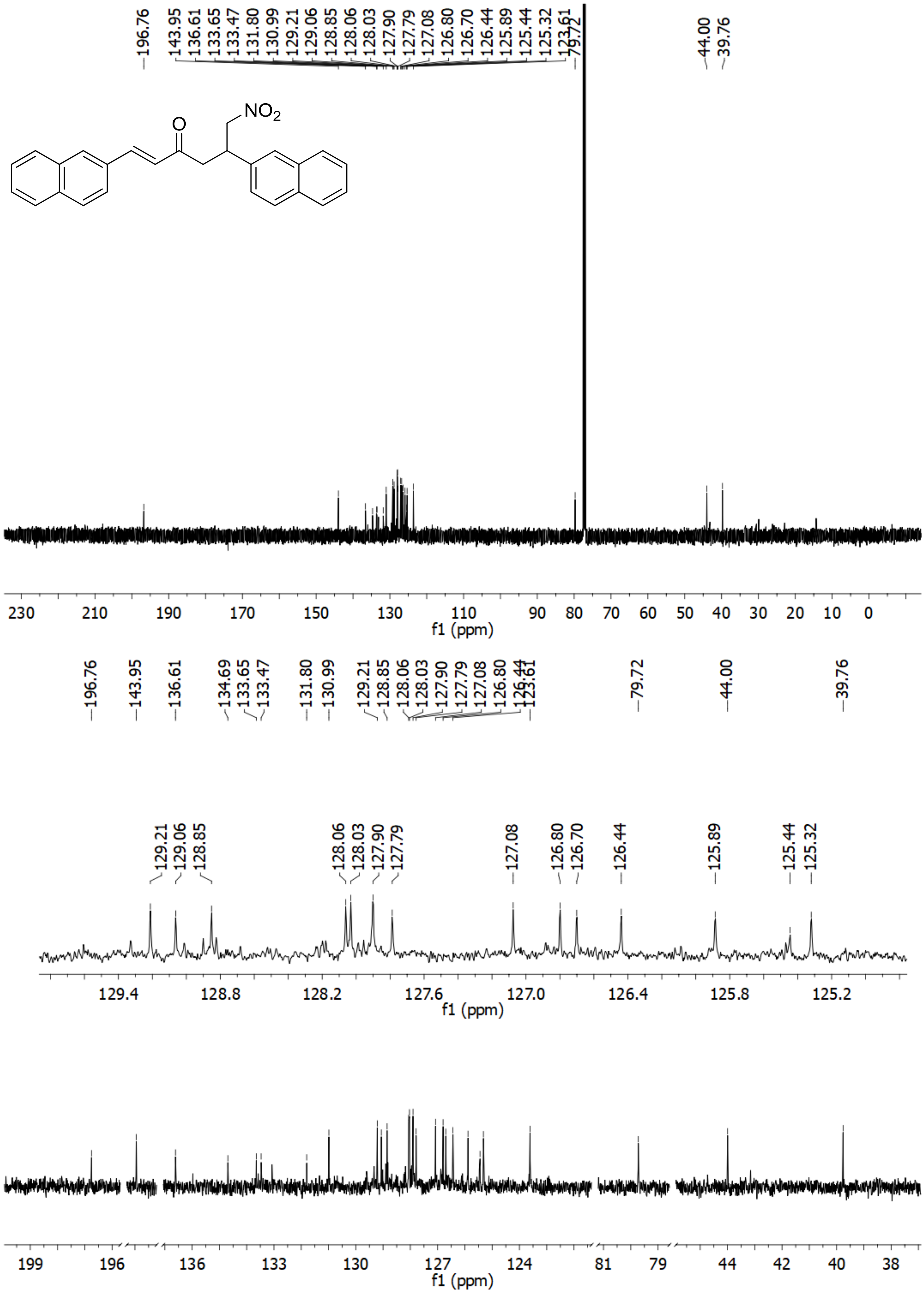

Figure S47: *^{13}C-NMR spectrum (125 MHz, $CDCl_3$) for the pure product (E)-1,5-di(naphthalen-2-yl)-6-nitrohex-1-en-3-one (4t): full scale spectrum (top) and spectrum expansions (bottom).*

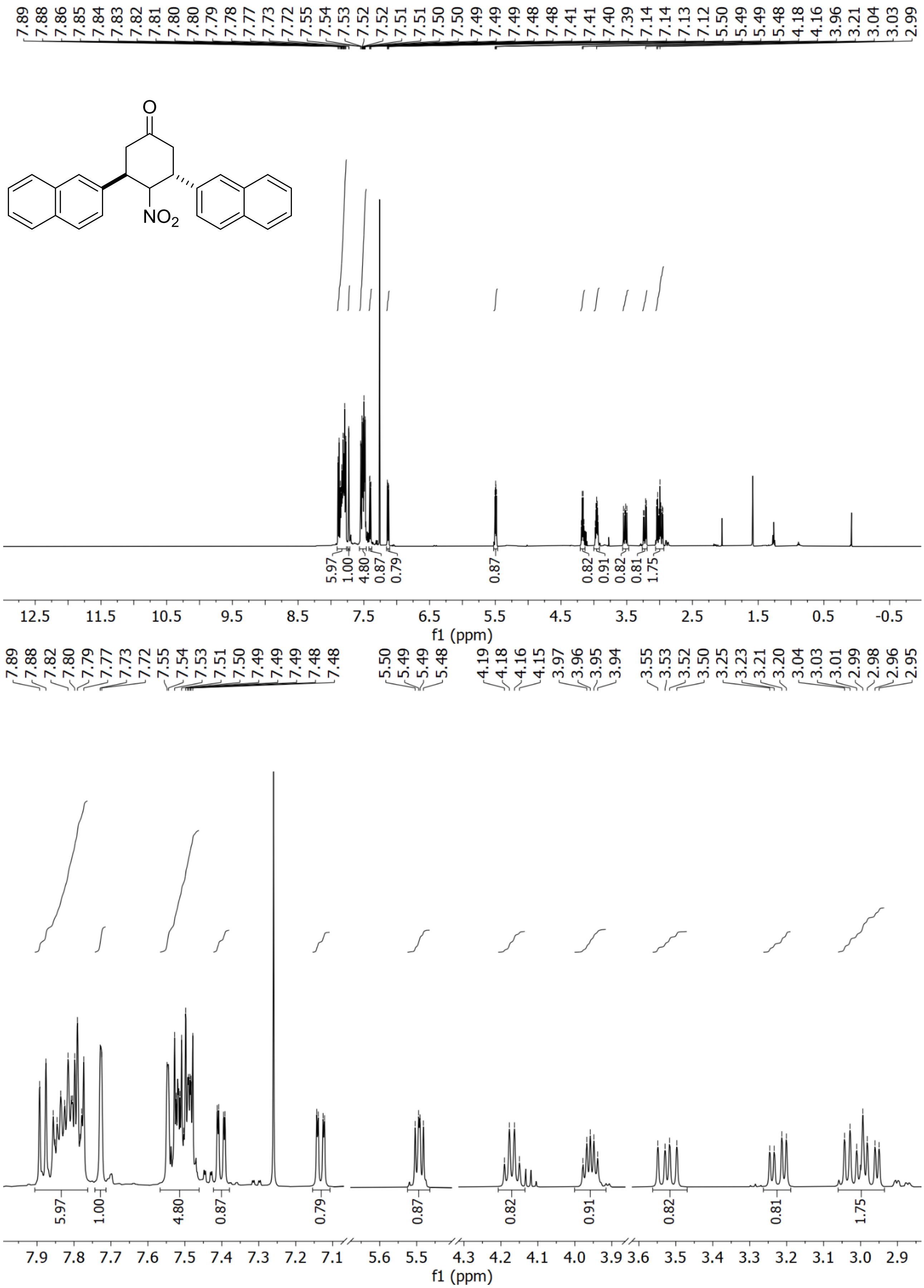

Figure S48: *^{1}H-NMR spectrum (500 MHz, $CDCl_3$) for the pure product anti-3,5-di(naphthalen-2-yl)-4-nitrocyclohexan-1-one (6t): full scale spectrum (top) and spectrum expansions (bottom).*

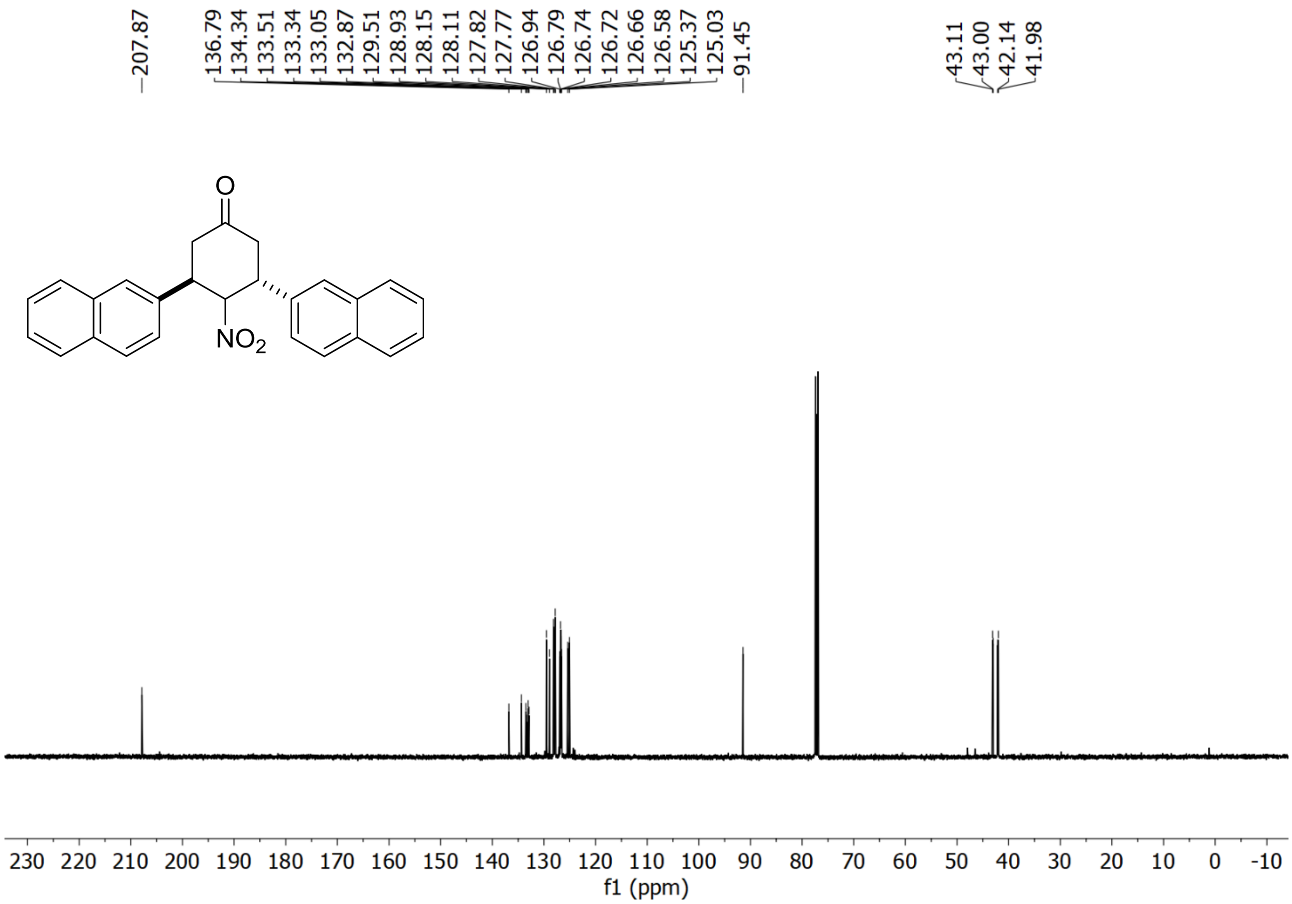

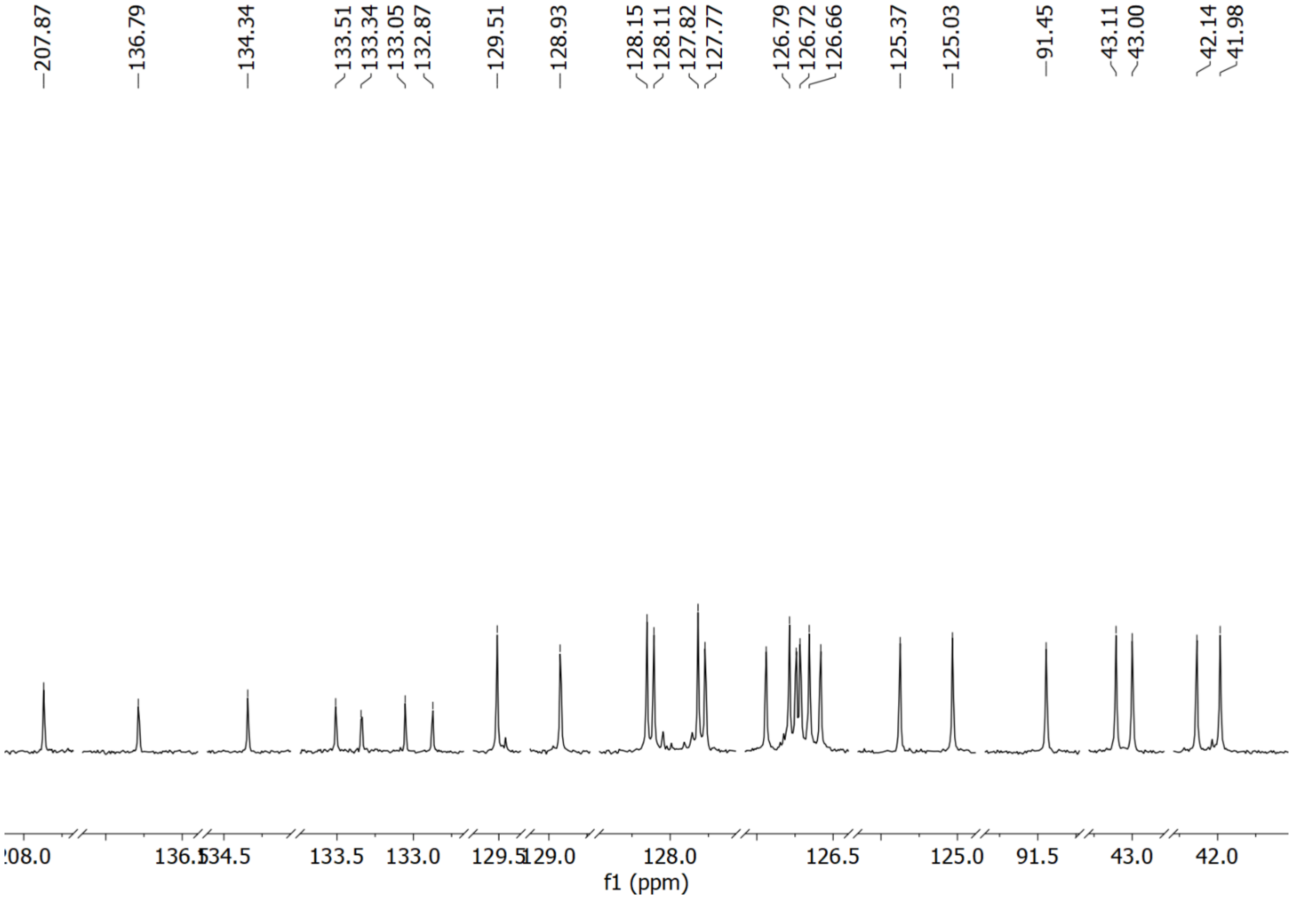

Figure S49: *^{13}C-NMR spectrum (125 MHz, $CDCl_3$) for the pure product anti-3,5-di(naphthalen-2-yl)-4-nitrocyclohexan-1-one (6t): full scale spectrum (top) and spectrum expansions (bottom).*

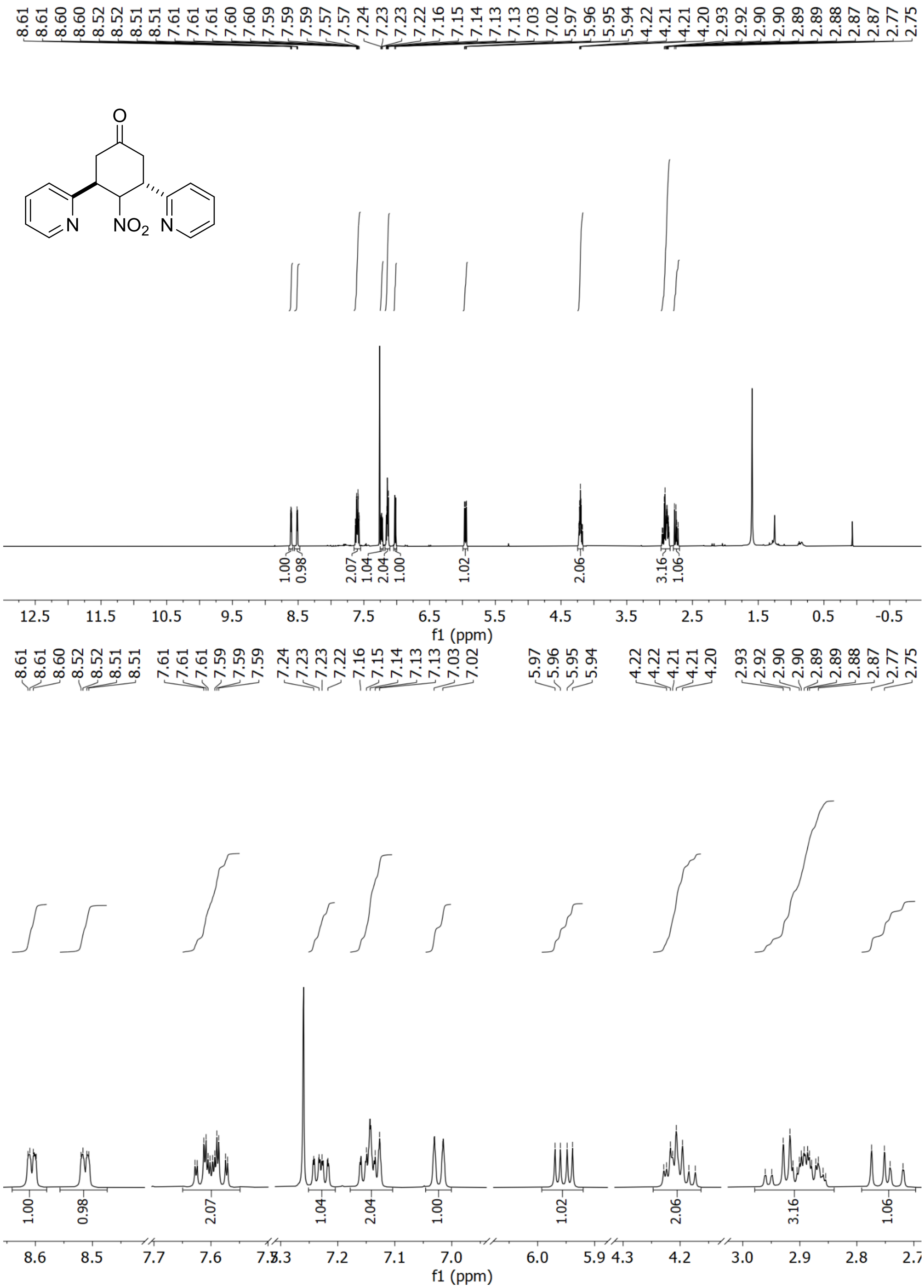

Figure S50: *^{1}H-NMR spectrum (500 MHz, $CDCl_3$) for the pure product anti-4-nitro-3,5-di(pyridin-2-yl)cyclohexan-1-one (6u): full scale spectrum (top) and spectrum expansions (bottom).*

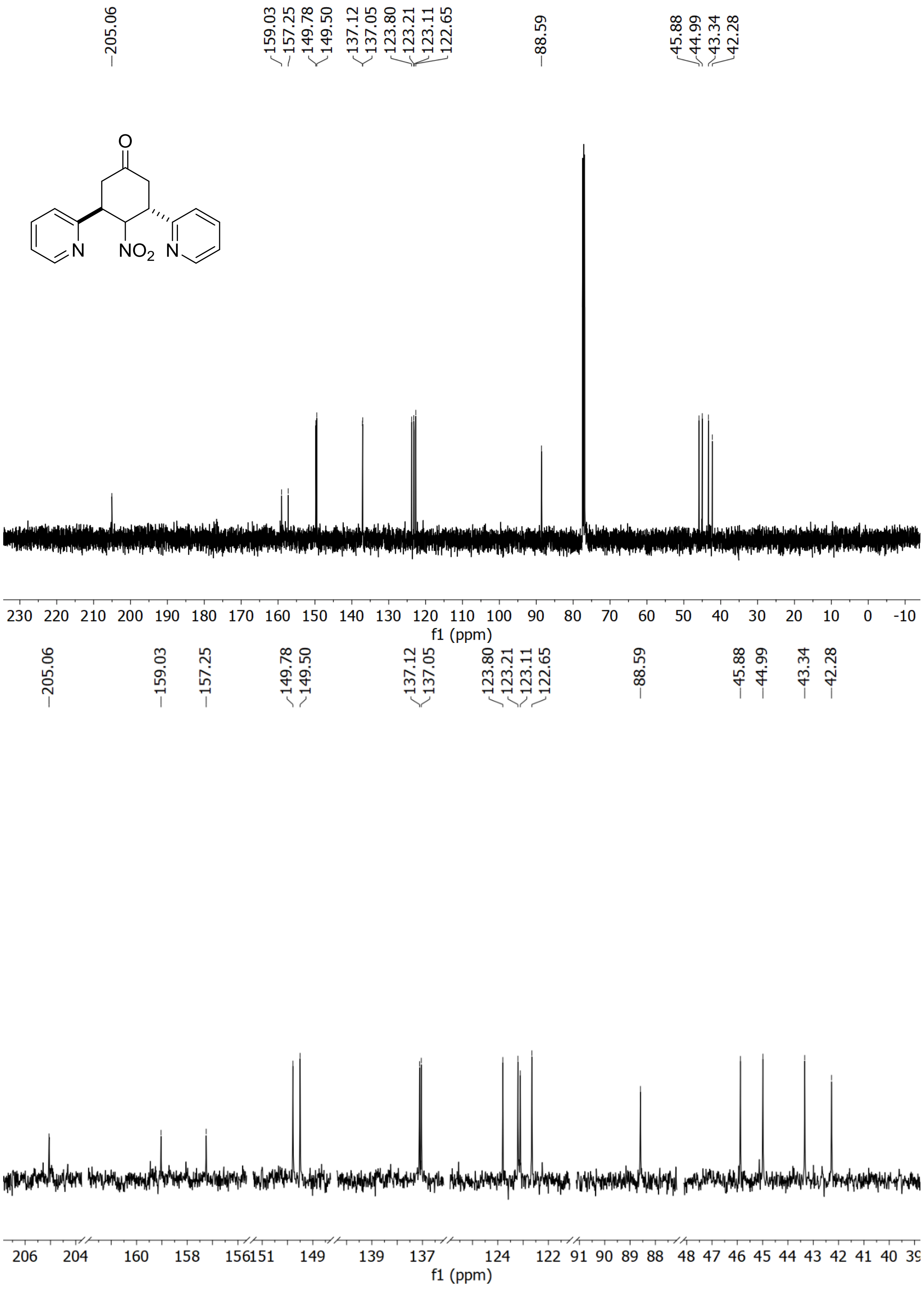

Figure S51: *^{13}C-NMR spectrum (125 MHz, $CDCl_3$) for the pure product anti-4-nitro-3,5-di(pyridin-2-yl)cyclohexan-1-one (6u): full scale spectrum (top) and spectrum expansions (bottom).*

Bibliography

[1] D. N. Rockwood, R. C. Preda, T. Yücel, X. Wang, M. L. Lovett, D. L. Kaplan, *Nat. Protoc.* **2011**, *6*, 1612-1631.

[2] M. Patel, D. K. Dubey, S. P. Singh, *Materials Science and Engineering: C* **2020**, *108*, 110414.

[3] E. Carr, *Textile Research* **1938**, *8*, 399-405.

[4] aA. Guida, M. H. Lhouty, D. Tichit, F. Figueras, P. Geneste, *Applied Catalysis A: General* **1997**, *164*, 251-264; bE. Morgan, Elsevier Current Trends, **1990**.

[5] A. Gorman, J. Killoran, C. O'Shea, T. Kenna, W. M. Gallagher, D. F. O'Shea, *Journal of the American Chemical Society* **2004**, *126*, 10619-10631.

[6] W. Yang, D.-M. Du, *Organic Letters* **2010**, *12*, 5450-5453.

[7] M. Talukdar, N. Islam, A. Mishra, A. Y. Dave, E. Begari, *Synlett* **2024**.

[8] C. Wang, C. Daddario, S. Pejić, G. Sauvé, *European Journal of Organic Chemistry* **2020**, *2020*, 714-722.

[9] M. Tsakos, C. G. Kokotos, G. Kokotos, *Advanced Synthesis & Catalysis* **2012**, *354*, 740-746.
